# Supplementary material for: Is night-time light intensity associated with cardiovascular disease risk factors among adults in early-stage urbanisation in South India? A cross-sectional study of the Andhra Pradesh Children and Parents Study
Source: BMJ Open. 2020 Nov 19;10(11):e036213. doi: 10.1136/bmjopen-2019-036213 (PMC7678398; doi:10.1136/bmjopen-2019-036213)
Supplement: Supplementary data [file bmjopen-2019-036213supp001.pdf]

SUPPLEMENTARY FILES

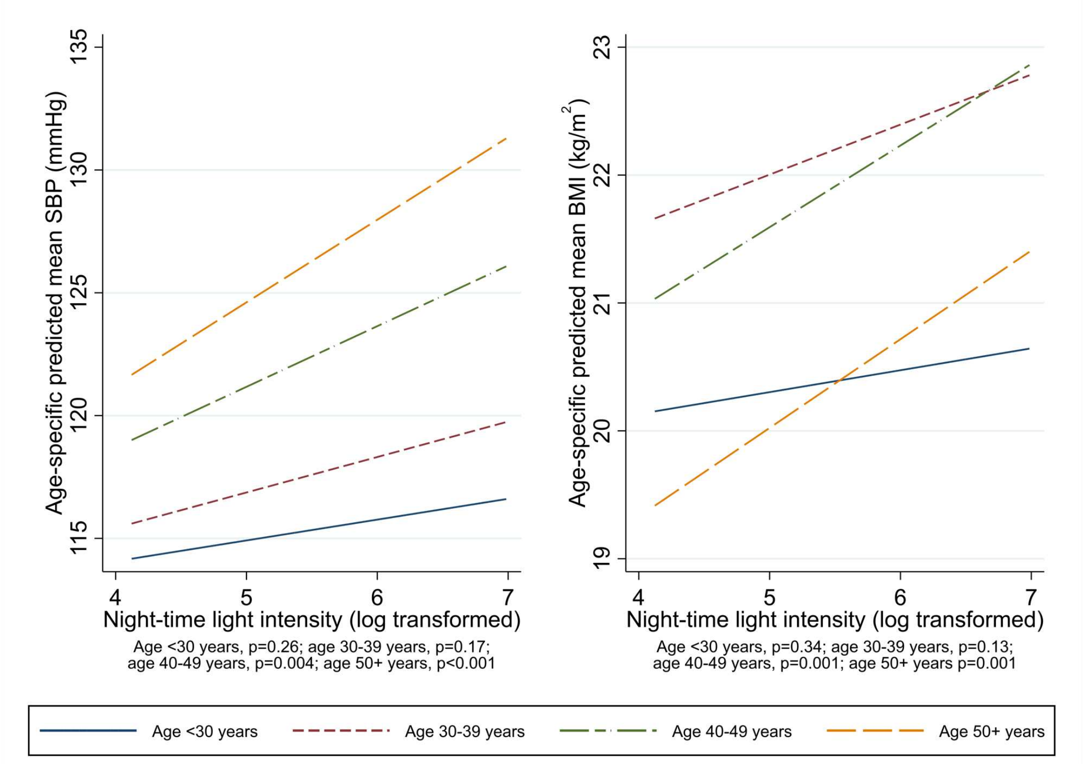

Supplementary Fig. 1 Age-specific crude associations of NTLI with mean BMI and SBP, APCAPS, 2010-12 (n=5937)

APCAPS – Andhra Pradesh Children and Parents Study; BMI – body mass index; NTLI – night-time light intensity; SBP – systolic blood pressure

The fitted lines (model predicted means) were derived from crude multilevel linear regression models with clustering by household and village, using individual-level outcome data

Participants were excluded from analysis of SBP if medicated for hypertension

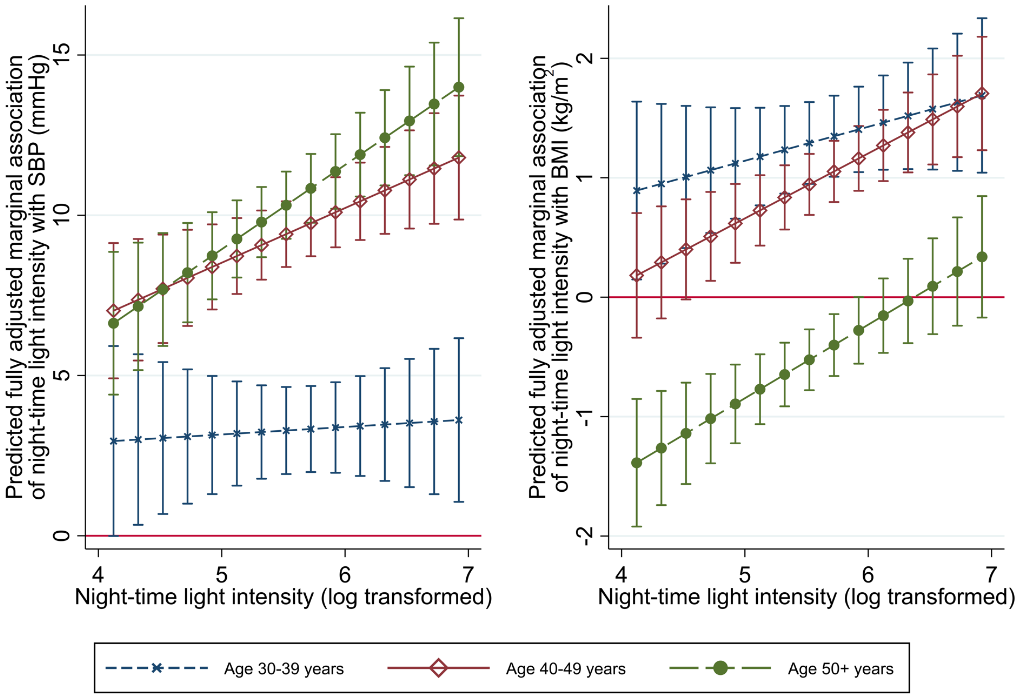

Supplementary Fig. 2 Fully adjusted marginal associations of NTLI with BMI and SBP by age groups (n=5937)

BMI – body mass index; CI – confidence interval; NTLI – night-time light intensity; SBP – systolic blood pressure

Marginal associations, including 95% CIs, were obtained from contrasting age-specific results from multilevel linear regression models with clustering by household and village, and adjusted for gender, caste, religion, marital status and survey season, using individual-level outcome data

Participants were excluded from analysis of SBP if medicated for hypertension

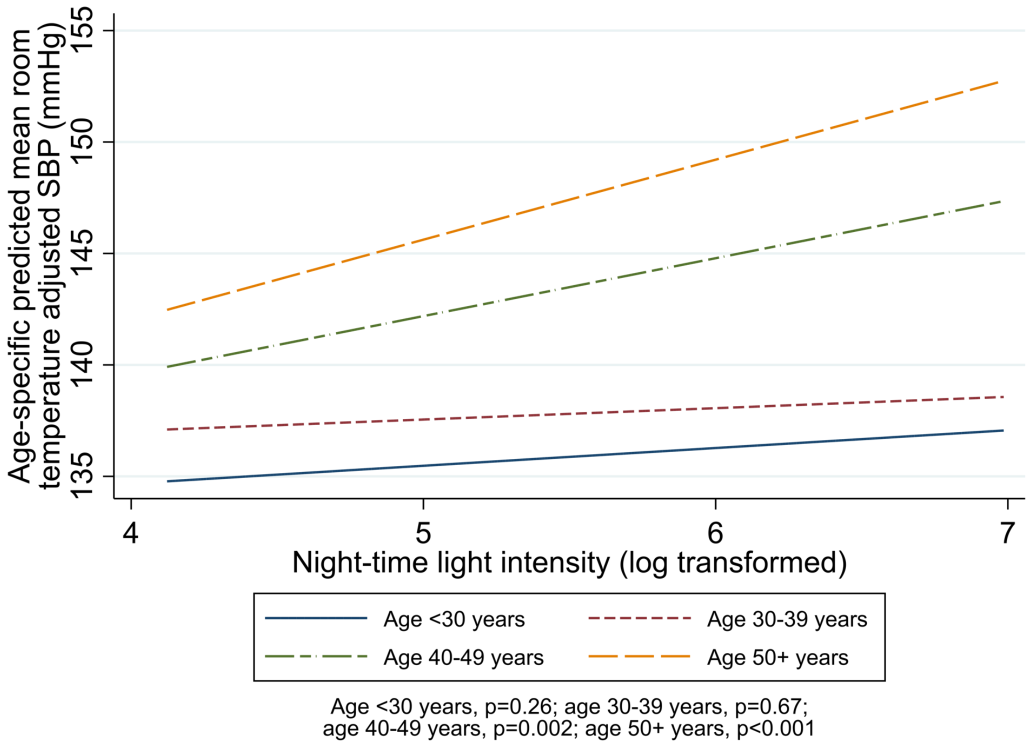

Supplementary Fig. 3 Age-specific room temperature adjusted association of NTLI and SBP among APCAPS adults, 2010-12 (n=3160)

APCAPS – Andhra Pradesh Children and Parents Study; NTLI – night-time light intensity; SBP – systolic blood pressure

The fitted lines (model predicted means) were derived from a crude multilevel linear regression models with clustering by household and village, using individual-level outcome data

Participants were excluded if medicated for hypertensions

Sorensen TB, *et al. BMJ Open* 2020; 10:e036213. doi: 10.1136/bmjopen-2019-036213

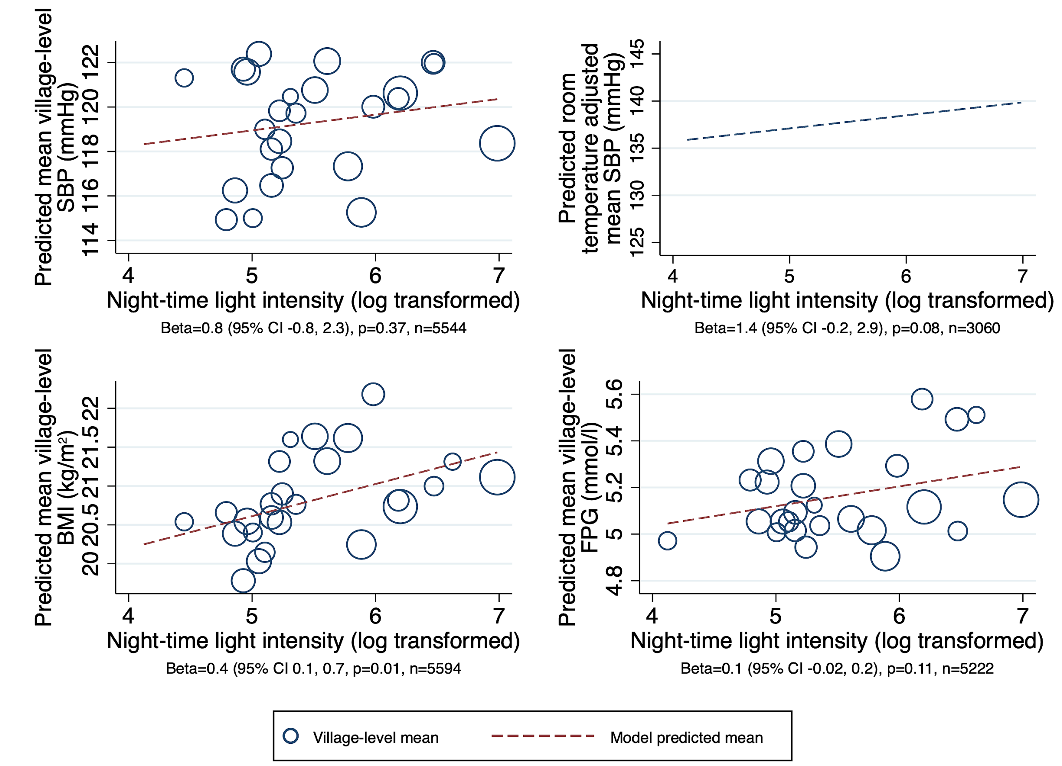

Supplementary Fig. 4 Sensitivity analysis: Crude association of NTLI with SBP, BMI and FPG among APCAPS adults, 2010-12

APCAPS – Andhra Pradesh Children and Parents Study; BMI – body mass index; FPG – fasting plasma glucose; NTLI – night-time light intensity; SBP – systolic blood pressure

Model predicted means were derived from crude and (for SBP) room temperature adjusted multilevel linear regression models with clustering by household and village, using individual-level outcome data

Marker size proportional to village size

Participants from borderline outlier villages were excluded

Sorensen TB, *et al. BMJ Open* 2020; 10:e036213. doi: 10.1136/bmjopen-2019-036213

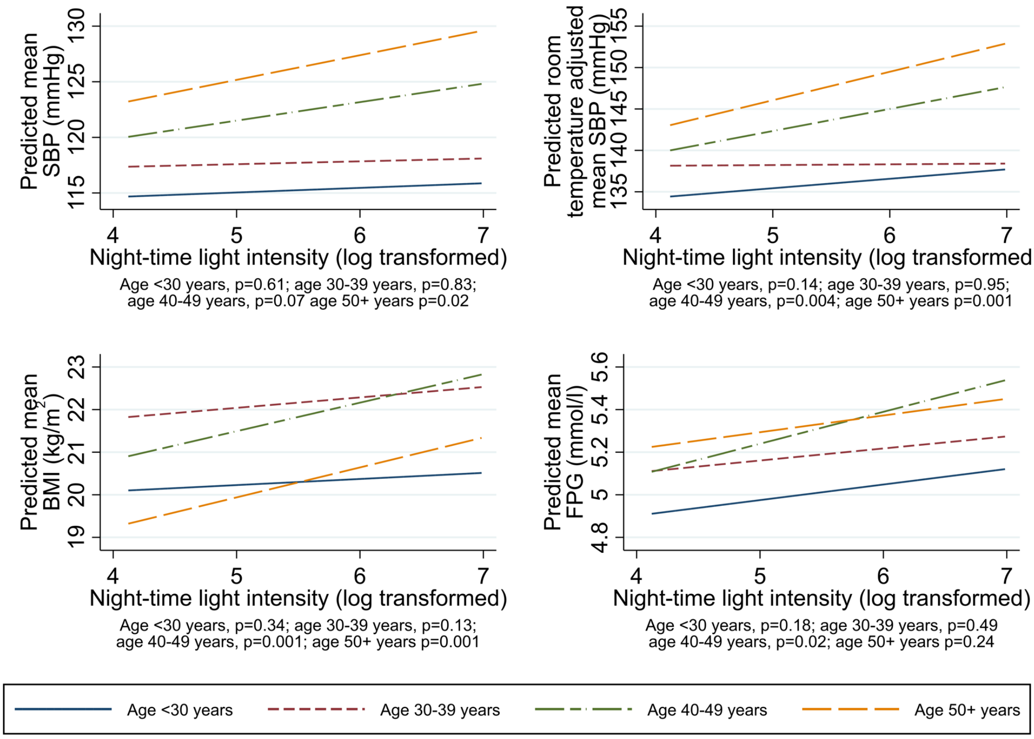

Supplementary Fig. 5 Sensitivity analysis: age-specific crude associations of NTLI with SBP, BMI and FPG

BMI – body mass index; FPG – fasting glucose; NTLI – night-time light intensity; SBP – systolic blood pressure

The fitted lines (model predicted means) were derived from a crude multilevel linear regression models with clustering by household and village, using individual-level outcome data

Participants from borderline outlier villages were excluded

Participants were excluded from analysis of SBP if medicated for hypertension and from analysis of FPG if medicated for diabetes

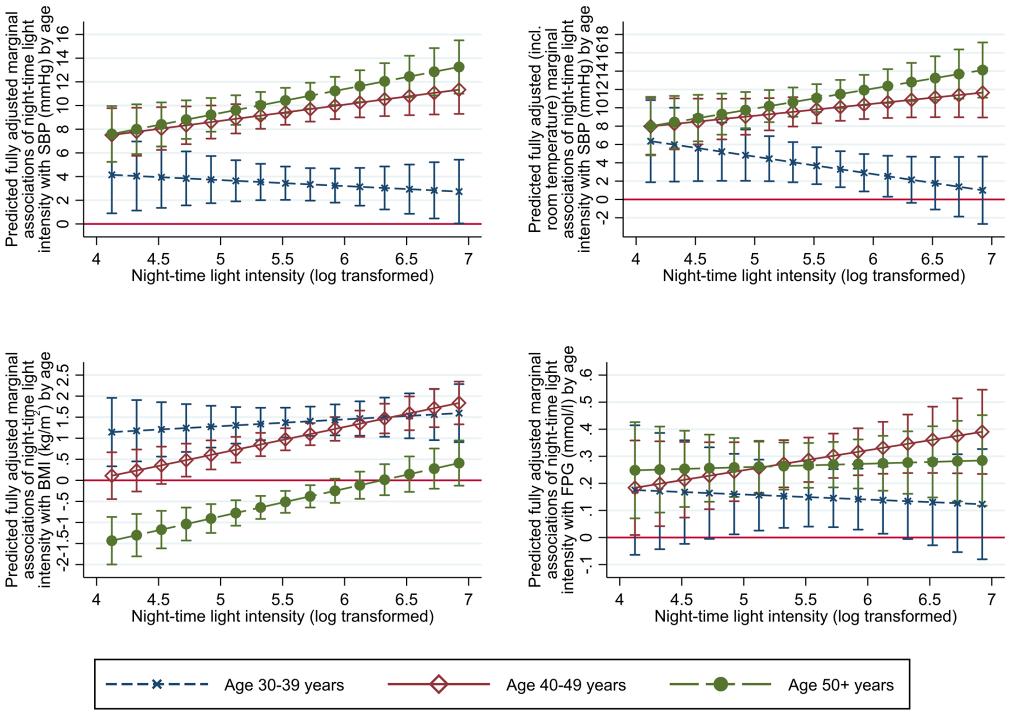

Supplementary Fig. 6 Sensitivity analysis: fully adjusted marginal associations of NTLI with SBP, BMI and FPG by age groups

BMI – body mass index; CI – Confidence Interval; FPG – fasting glucose; SBP – systolic blood pressure

Marginal associations (95% CIs) were obtained from contrasting age-specific results from multilevel linear regression models with clustering by household and village, and adjusted for gender, caste, religion, marital status and survey season, using individual level outcome data

Participants from borderline outlier villages were excluded

Participants were excluded from analysis of SBP if medicated for hypertension and from analysis of glucose if medicated for diabetes

Sorensen TB, *et al. BMJ Open* 2020; 10:e036213. doi: 10.1136/bmjopen-2019-036213

Supplementary Tab. 1 Age-specific crude and adjusted associations of NTLI with mean BMI and SBP, APCAPS, 2010-12 (n=5937)

**CVD risk factors**	**Age group**	**n (%)**	**Age-specific crude model predicted mean (95% CI) at the lowest NTLI (61.7 (4.1 on the log scale))**	**Age-specific crude model predicted mean (95% CI) at the highest NTLI (1081.1 (7.0 on the log scale))**	**p-value**	**Age-specific fully adjusted model predicted mean change with increasing NTLI[b] (adjusted for gender caste, religion, marital status and survey season)**	
						**β (95% CI)**	**p-value**
**SBP (mmHg)[a]**	<30 years	2806 (48.8)	114.2 (111.9, 116.4)	116.6 (114.2, 119.0)	0.26	0.5 (-0.7, 1.6)	0.45
	30-39 years	544 (9.5)	115.6 (112.2, 119)	119.7 (116.5, 123.0)	0.17	0.7 (-1.2, 2.5)	0.47
	40-49 years	1306 (22.7)	119.0 (116.4, 121.6)	126.1 (123.4, 128.8)	0.004	2.2 (0.8, 3.6)	0.003
	≥50 years	1096 (19.1)	121.7 (118.9, 124.4)	131.3 (128.4, 134.2)	<0.001	3.1 (1.6, 4.6)	<0.001
**BMI (kg/m^2)**	<30 years	2804 (47.3)	20.2 (19.6, 20.7)	20.6 (20.1, 21.2)	0.34	0.04 (-0.3, 0.4)	0.84
	30-39 years	545 (9.2)	21.7 (20.8, 22.5)	22.8 (22.0, 23.6)	0.13	0.3 (-0.2, 0.8)	0.22
	40-49 years	1358 (22.9)	21.0 (20.4, 21.7)	22.9 (22.2, 23.5)	0.001	0.6 (0.2, 1.0)	0.01
	≥50 years	1217 (20.5)	19.4 (18.8, 20.0)	21.4 (20.7, 22.1)	0.001	0.7 (0.2, 1.1)	0.002

APCAPS – Andhra Pradesh Children and Parents Study; BMI – body mass index; CVD – cardiovascular disease; NTLI – night-time light intensity; SBP – systolic blood pressure; β – beta coefficient

Model predicted means (95% CIs), β-coefficients (95% CIs) and p-values were obtained from multilevel linear regression models with clustering by household and village, using individual-level outcome data

[a]Participants were excluded if medicated for hypertension

[b]Mean change per integer increase in log transformed NTLI

Supplementary Tab. 2 Room temperature adjusted association of NTLI and SBP among APCAPS adults, 2010-12 (n=3160)

					**Model predicted age, gender and room temperature adjusted mean change (95% CI) with increasing NTLI[b]**			**Model predicted fully adjusted mean change with increasing NTLI[b] (adjusted for gender caste, religion, marital status, survey season and room temperature)**		
	**n**	**Model predicted room temperature adjusted mean (95% CI) at the lowest NTLI (61.7 (4.1 on the log scale))**	**Model predicted room temperature adjusted mean (95% CI) at the highest NTLI (1081.1 (7.0 on the log scale))**	**p-value**	**n**	**β (95% CI)**	**p-value**	**n**	**β (95% CI)**	**p-value**
**SBP (mmHg)[a]**	3160	136.4 (129.0, 143.8)	140.7 (133.5, 148.0)	<0.001	3159	1.9 (0.7, 3.0)	0.002	3122	1.8 (0.6, 3.0)	0.002

APCAPS – Andhra Pradesh Children and Parents Study; CI – confidence interval; n – sample size; NTLI – night-time light intensity; SBP – systolic blood pressure; β – beta-coefficient

Model predicted means (95% CIs), β-coefficients (95% CIs) and p-values were obtained from multilevel linear regression with clustering by household and village, using individual-level outcome data

[a]Participants were excluded if medicated for hypertension

[b]Mean change per integer increase in log transformed NTLI

Supplementary Tab. 3 Age-specific room temperature adjusted association of NTLI and SBP among APCAPS adults, 2010-12 (n=3160)

SBP (mmHg)[a]	n (%)	Model predicted room temperature adjusted mean (95% CI) at the lowest NTLI (61.7 (4.1 on the log scale))	Model predicted room temperature adjusted mean (95% CI) at the highest NTLI (1081.1 (7.0 on the log scale))	p-value	Model predicted fully adjusted mean change with increasing NTLI[b] (adjusted for gender caste, religion, marital status, survey season and room temperature at clinical assessment)	
					β (95% CI)	p-value
<30 years	1347 (42.6)	134.8 (128.1, 141.4)	137.1 (130.4, 143.7)	0.26	0.8 (-0.5, 2.1)	0.24
30-39 years	305 (9.7)	137.1 (129.6, 144.6)	138.6 (131.3, 145.8)	0.67	-0.2 (-2.5, 2.0)	0.83
40-49 years	838 (26.5)	139.9 (133.0, 146.8)	147.3 (140.6, 154.1)	0.002	2.4 (0.8, 4.0)	0.003
≥50 years	670 (21.2)	142.5 (135.6, 149.4)	152.7 (145.8, 159.7)	<0.001	3.5 (1.8, 5.3)	<0.001

APCAPS – Andhra Pradesh Children and Parents Study; CI – confidence interval; n – sample size; NTLI – night-time light intensity; SBP – systolic blood pressure; β – beta coefficient

Model predicted means (95% CIs), β-coefficients (95% CIs) and p-values were obtained from multilevel linear regression with clustering by household and village, using individual-level outcome data

p<0.001 for effect modification by age group

[a]Participants were excluded if medicated for hypertension

[b]Mean change per integer increase in log transformed NTLI

Supplementary Tab. 4 Sensitivity analysis: crude and adjusted associations of NTLI with SBP, BMI and FPG excluding borderline outlier villages

	Model predicted crude mean change with increasing NTLI[c]			Model predicted age and gender adjusted mean change with increasing NTLI[c]			Model predicted fully adjusted mean change with increasing NTLI[c] (adjusted for age, gender, caste, religion, marital status and survey season)		
**CVD risk factors**	**n**	**β (95% CI)**	**p-value**	**n**	**β (95% CI)**	**p-value**	**n**	**β (95% CI)**	**p-value**
**SBP (mmHg)[a]**	5544	0.8 (-0.8, 2.3)	0.37	5539	1 .0 (-0.5, 2.5)	0.19	5306	0.8 (-0.4, 2.1)	0.18
**SBP (mmHg), room temperature adjusted[a]**	3060	1.4 (-0.2, 2.9)	0.08	3059	1.8 (0.5, 3.1)	0.01	3022	1.7 (0.4, 3.0)	0.01
**BMI (kg/m2)**	5594	0.4 (0.1, 0.7)	0.01	5590	0.4 (0.1, 0.7)	0.01	5352	0.3 (0.03, 0.7)	0.03
**Fasting glucose (mmol/l)[b]**	5222	0.1 (-0.02, 0.2)	0.11	5220	0.1 (-0.01, 0.2)	0.08	4988	0.1 (-0.06, 0.2)	0.37

BMI – body mass index; CI – confidence interval; CVD – cardiovascular disease; LDL – low-density lipoprotein; n – sample size; NTLI – night-time light intensity; SBP – systolic blood pressure; β – beta coefficient

β-coefficients (95% CIs) and p-values were obtained from multilevel linear regression with clustering by household and village, using individual-level outcome data

Participants from borderline outlier villages were excluded

Participants were excluded if medicated for hypertension[a] or diabetes[b]

[c]Mean change per integer increase in log transformed NTLI

Sorensen TB, *et al. BMJ Open* 2020; 10:e036213. doi: 10.1136/bmjopen-2019-036213

Supplementary Tab. 5 Sensitivity analysis: age-specific crude and adjusted associations of NTLI with SBP, BMI and FPG excluding borderline outlier villages

CVD risk factors		n	Age-specific model predicted mean (95% CI) at lowest NTLI	Age-specific model predicted mean (95% CI) at highest NTLI	p-value	Age-specific fully adjusted model predicted mean change with increasing NTLI[c] (adjusted for gender caste, religion, marital status and survey season)		
						n	β (95% CI)	p-value
**SBP (mmHg)[a]**	<30 years	2706	114.8 (112.8, 116.8)	115.9 (113.3, 118.4)	0.61	2615	0.2 (-1.1, 1.5)	0.79
	30-39 years	520	117.4 (114.4, 120.5)	118.1 (114.6, 121.5)	0.83	494	-0.3 (-2.4, 1.7)	0.76
	40-49 years	1252	120.6 (118.3, 122.9)	124.8 (121.9, 127.7)	0.07	1196	1.5 (0.02, 3.1)	0.05
	≥50 years	1066	123.9 (121.6, 126.3)	129.6 (126.5, 132.6)	0.02	1001	2.2 (0.5, 3.8)	0.01
**SBP (mmHg)[a] adjusted for room temperature at clinical assessment**	<30 years	1308	134.8 (127.9, 141.7)	137.7 (130.3, 145.1)	0.14	1294	1.0 (-0.5, 2.5)	0.20
	30-39 years	292	138.2 (130.6, 145.7)	138.4 (130.4, 146.4)	0.95	285	-0.8 (-3.4, 1.8)	0.54
	40-49 years	809	140.9 (133.8, 148.0)	147.6 (140.1, 155.2)	0.004	799	2.3 (0.6, 4.1)	0.01
	≥50 years	651	144.2 (137.1, 151.2)	152.9 (145.2, 160.6)	0.001	644	3.2 (1.3, 5.1)	0.001
**BMI (kg/m2)**	<30 years	2653	20.1 (19.7, 20.6)	20.5 (20.0, 21.1)	0.42	2563	0.02 (-0.3, 0.4)	0.92
	30-39 years	508	21.9 (21.2, 22.6)	22.5 (21.7, 23.3)	0.37	482	0.2 (-0.3, 0.7)	0.51
	40-49 years	1272	21.1 (20.6, 21.6)	22.8 (22.2, 23.5)	0.001	1213	0.6 (0.2, 1.0)	0.002
	≥50 years	1161	19.6 (19.0, 20.1)	21.3 (20.7, 22.0)	<0.001	1094	0.7 (0.3, 1.1)	0.001
**Fasting plasma glucose[b]**	<30 years	2537	4.9 (4.7, 5.1)	5.1 (4.9, 5.3)	0.20	2450	0.03 (-0.1, 0.2)	0.59
	30-39 years	491	5.1 (4.9, 5.4)	5.3 (5.0, 5.5)	0.49	465	0.01 (-0.2, 0.2)	0.88
	40-49 years	1170	5.1 (4.9, 5.3)	5.5 (5.3, 5.7)	0.02	1114	0.1 (-0.03, 0.2)	0.13
	≥50 years	1024	5.2 (5.0, 5.4)	5.4 (5.2, 5.7)	0.25	959	0.05 (-0.1, 0.2)	0.52

Sorensen TB, *et al. BMJ Open* 2020; 10:e036213. doi: 10.1136/bmjopen-2019-036213

BMJ Publishing Group Limited (BMJ) disclaims all liability and responsibility arising from any reliance placed on this supplemental material which has been supplied by the author(s)

BMI – body mass index; CI - confidence interval; n – sample size; NTLI – night-time light intensity; SBP – systolic blood pressure; β – beta coefficient

Model predicted means (95% CIs), β-coefficients (95% CIs) and p-values were obtained from multilevel linear regression models with clustering by household and village, using individual-level outcome data

Participants from borderline outlier villages were excluded

Participants were excluded if medicated for hypertension[a] or diabetes[b]

[c]Mean change per integer increase in log transformed NTLI

Sorensen TB, *et al. BMJ Open* 2020; 10:e036213. doi: 10.1136/bmjopen-2019-036213
