# Supplementary material for: Is night-time light intensity associated with cardiovascular disease risk factors among adults in early-stage urbanisation in South India? A cross-sectional study of the Andhra Pradesh Children and Parents Study
Source: BMJ Open. 2020 Nov 19;10(11):e036213. doi: 10.1136/bmjopen-2019-036213 (PMC7678398; doi:10.1136/bmjopen-2019-036213)
Supplement: Author's manuscript [file bmjopen-2019-036213.draft_revisions.pdf]

BMJ Open is committed to open peer review. As part of this commitment we make the peer review history of every article we publish publicly available.

When an article is published we post the peer reviewers' comments and the authors' responses online. We also post the versions of the paper that were used during peer review. These are the versions that the peer review comments apply to.

The versions of the paper that follow are the versions that were submitted during the peer review process. They are not the versions of record or the final published versions. They should not be cited or distributed as the published version of this manuscript.

BMJ Open is an open access journal and the full, final, typeset and author-corrected version of record of the manuscript is available on our site with no access controls, subscription charges or pay-per-view fees (http://bmjopen.bmj.com).

If you have any questions on BMJ Open's open peer review process please email info.bmjopen@bmj.com

BMJ Open

NIGHT-TIME LIGHT INTENSITY CONSISTENTLY ASSOCIATED WITH CARDIOVASCULAR DISEASE RISK FACTORS: A CROSS SECTIONAL STUDY OF THE ANDHRA PRADESH CHILDREN AND PARENTS STUDY, SOUTH INDIA

Journal:	*BMJ Open*
Manuscript ID	bmjopen-2019-036213
Article Type:	Original research
Date Submitted by the Author:	05-Dec-2019
Complete List of Authors:	Sorensen, Tina Bonde; London School of Hygiene and Tropical Medicine, Department of Non-communicable Disease Epidemiology<br>Wilson, Robin; University of Southampton, Geography & Environment<br>Gregson, John; London School of Hygiene and Tropical Medicine, Medical Statistics<br>Shankar, Bhavani; SOAS, Centre for Development, Environment and Policy<br>Dangour, Alan; London School of Hygiene and Tropical Medicine, Population Health<br>Kinra, Sanjay; London School of Hygiene and Tropical Medicine, Non-communicable Disease Epidemiology
Keywords:	EPIDEMIOLOGY, Cardiac Epidemiology < CARDIOLOGY, Hypertension < CARDIOLOGY

SCHOLARONE™
Manuscripts

I, the Submitting Author has the right to grant and does grant on behalf of all authors of the Work (as defined in the below author licence), an exclusive licence and/or a non-exclusive licence for contributions from authors who are: i) UK Crown employees; ii) where BMJ has agreed a CC-BY licence shall apply, and/or iii) in accordance with the terms applicable for US Federal Government officers or employees acting as part of their official duties; on a worldwide, perpetual, irrevocable, royalty-free basis to BMJ Publishing Group Ltd ("BMJ") its licensees and where the relevant Journal is co-owned by BMJ to the co-owners of the Journal, to publish the Work in this journal and any other BMJ products and to exploit all rights, as set out in our licence.

The Submitting Author accepts and understands that any supply made under these terms is made by BMJ to the Submitting Author unless you are acting as an employee on behalf of your employer or a postgraduate student of an affiliated institution which is paying any applicable article publishing charge ("APC") for Open Access articles. Where the Submitting Author wishes to make the Work available on an Open Access basis (and intends to pay the relevant APC), the terms of reuse of such Open Access shall be governed by a Creative Commons licence – details of these licences and which Creative Commons licence will apply to this Work are set out in our licence referred to above.

Other than as permitted in any relevant BMJ Author's Self Archiving Policies, I confirm this Work has not been accepted for publication elsewhere, is not being considered for publication elsewhere and does not duplicate material already published. I confirm all authors consent to publication of this Work and authorise the granting of this licence.

For peer review only

NIGHT-TIME LIGHT INTENSITY CONSISTENTLY ASSOCIATED WITH CARDIOVASCULAR DISEASE RISK FACTORS: A CROSS SECTIONAL STUDY OF THE ANDHRA PRADESH CHILDREN AND PARENTS STUDY, SOUTH INDIA

Tina B Sørensen,[1*] Robin Wilson,[2] John Gregson,[1] Bhavani Shankar,[3,4] Alan D Dangour[1,4] and Sanjay Kinra[1]

*Corresponding author. Faculty of Epidemiology and Population Health, London School of Hygiene and Tropical Medicine (LSHTM), London WC1E 7HT, United Kingdom. E-mail: tina.sorensen@lshtm.ac.uk, Telephone +44 (0) 2072994723. Orcid: https://orcid.org/0000-0003-1517-6159

[1]Faculty of Epidemiology and Population Health, London School of Hygiene and Tropical Medicine, Keppel Street, London WC1E 7HT, UK

[2]Geography & Environment, University of Southampton, University Road, Southampton SO17 1BJ, UK

[3]Centre for Development, Environment and Policy, School of Oriental and African Studies, 10 Thornhaugh Street, Russell Square, London WC1H 0XG, UK

[4]Leverhulme Centre for Integrative Research in Agriculture and Health (LCIRAH), 36 Gordon Square, London WC1H 0PD, UK

E-mails: tina.sorensen@lshtm.ac.uk, robin@rtwilson.com, john.gregson@lshtm.ac.uk, b.shankar@soas.ac.uk, alan.dangour@lshtm.ac.uk, sanjay.kinra@lshtm.ac.uk

Word count 279 words (abstract), 4088 words (text)

For peer review only

ABSTRACT

Objectives: Explore associations of night-time light intensity (NTLI), a novel proxy for continuous urbanisation levels, with mean levels of systolic blood pressure (SBP), body mass index (BMI), fasting serum low-density lipoprotein (LDL), and fasting plasma glucose (FPG), among adults at early stages of urbanisation in Telangana, South India.

Design: Cross-sectional analysis of cluster-randomised cohort data (third wave of the Andhra Pradesh Children and Parents Study).

Setting: 28 villages representing a continuum of urbanisation levels, ranging from rural settlement to medium sized town in Telangana, South India.

Participants: Data were available from 6944 participants, 6236 of whom were eligible after excluding pregnant women, participants younger than 18 years of age and participants missing data for age. Participants were excluded if they were not fasting, had implausible or missing outcome values, were medicated for hypertension or diabetes, or had triglyceride levels invalidating derived LDL. The analysis included 5924 participants for BMI, 5752 participants for SBP, 5287 participants for LDL and 5328 participants for FPG.

Results: Increasing NTLI was positively associated with mean BMI, SBP and LDL. Adjusted mean differences across the range of village-level NTLI were 1.0 kg/m^2 (95% CI 0.01, 1.9) for BMI; 4.2 mmHg (95% CI 1.0, 7.4) for SBP; 0.3 mmol/l (95% CI -0.01, 0.7) for LDL; and -0.01 mmol/l (95% CI -0.4, 0.4) for FPG. Associations of NTLI with BMI and SBP were stronger in older age groups.

Conclusion: The association of NTLI with CVD risk factors identify NTLI as a potentially important tool for exploring urbanisation-related health. Consistent associations of moderate increases in urbanisation levels with important CVD risk factors warrant prevention strategies to curb expected large public health impacts from continued, rapid urbanisation in India.

Key words: Urbanisation, night-time light intensity (NTLI), non-communicable disease (NCD), cardiovascular disease (CVD), CVD risk factor, Andhra Pradesh Children and Parents Study (APCAPS)

For peer review only

For peer review only - http://bmjopen.bmj.com/site/about/guidelines.xhtml

STRENGTHS AND LIMITATIONS OF THIS STUDY

- We used a novel, standardised proxy of continuous urbanisation levels, remote sensing data on night-time light intensity, measured at the village level
- The villages had experienced rapid asymmetric urbanisation during the decades preceding the study and represented a continuum of urbanisation levels ranging from a rural settlement to a medium sized town
- The study used a large cluster-randomised population-based sample from 28 urbanising villages at early stages of urbanisation
- Differences between excluded and included participants warranted some caution of generalising the findings to the general population
- The cross-sectional design prevented us from making causal inferences or exploring the impact of urbanisation rate on CVD risk factors

For peer review only

For peer review only - http://bmjopen.bmj.com/site/about/guidelines.xhtml

BACKGROUND

Cardiovascular diseases (CVDs), primarily coronary heart disease, are the leading causes of death (28%) and disability adjusted life years (14%) in India.[1] They occur 5-10 years earlier than in Western populations[2] and the age-standardised mortality rate (272 per 100000 persons) exceeds the global average and rates of some high-income countries.[3 4] Individuals and societies in India therefore continue to experience substantial losses of productivity and income.[1 5-7] Urbanisation is often described as a key driver of CVDs in low- and middle-income (LMICs) acting on CVD and CVD risk factors through interrelated changes in social, physical and build environments, socio-demographics, lifestyle and mental health. Some of these change are associated with harmful impacts on cardiovascular health, e.g. due to reduced physical activity and nutrition transitions, while others may be beneficial, e.g. through more and better education and access to health and social services.[8-12] A number of simple rural-urban comparisons and migration studies provide insight to the association of urbanisation level with CVD risk factors, such as overweight and obesity, hypertension, diabetes, and dyslipidaemia.[8 13-21] The broad rural-urban dichotomies are useful, however insufficient for exploring which factors of urbanisation are most important for health in LMICs.[22] In part because no universal definition exists of 'rural' and 'urban' environments, and data on neighbourhood characteristics are often not available from these settings.[22] Furthermore, residents' characteristics tend to be more similar within than between urban and rural areas. For example, urban residents may on average enjoy wider access to goods and services, higher levels of education, occupation, affluence etc., whereas the opposite may be the case in rural areas.[22 23] Such limited discordance at either end of the urbanisation continuum makes it difficult to tease out the underlying mechanisms of observed differences in CVD risk between the two extremes. Urbanisation factors may change at different rates with urbanisation,[22] however, and thus vary widely across the continuum from rural to urban areas. It may therefore be more useful and informative to study the impact of urbanisation level on CVD risk factors in transitioning populations, where the underlying mechanisms may be easier to separate. Unfortunately, it is challenging to accurately measure urbanisation as a continuum, whether using single- (e.g. population density) or multi-component classification systems (e.g. housing types and densities, economic activities, physical environment, services, etc.).[22] This is particularly true in LMICs where these types of data are often scarce. As an alternative to physical data based scales, remote sensing data have been widely used to characterise urban landscapes and scale of urbanisation.[24 25] The night-time light intensity (NTLI) data, obtained by the United States' Defence Meteorological Satellite Programme's Operational Linescan System (DMSP-OLS), are suggested a valid proxy for urbanisation level and dynamics due to their strong and consistent

For peer review only - http://bmjopen.bmj.com/site/about/guidelines.xhtml

relationships with demographic and economic indicators of urbanisation such as urban build up area, population density, economic activity, and energy consumption at global, regional and local levels.[24-27] The raw satellite data is processed into annual averages of light intensity from persistent sources associated with human settlement, i.e. excluding transient light sources such as lightning, fire; aurora, solar and lunar light. The processed data are aggregated into 30 arc seconds grids[28] and published as unitless digital number (DNs) ranging from 0 (no light) to 63 (light saturation).[24 27] The NTLI data, which have near global coverage and a freely available historical data archive dating back to 1992,[29] offer a unique opportunity to study the association of urbanisation, measured by a single standard measure world-wide, with CVD risk. This study aimed to explore associations of urbanisation level (measured by a continuum of NTLI) with mean levels of four leading risk factors for CVDs, systolic blood pressure (SBP), body mass index (BMI), fasting serum low-density lipoprotein (LDL), and fasting plasma glucose (FPG), in adults from 28 villages in rapidly urbanising Telangana, South India. Improved understanding of the association of urbanisation level and CVD risk factors in transitioning areas will help inform policy for disease prevention and control. Such policies are imperative in India, where economic growth and urbanisation continues, to avoid further adverse economic and social impacts of (premature) CVD morbidity and mortality.

MATERIALS AND METHODS

Study design, setting and participants

Data from the third survey wave of the Andhra Pradesh Children and Parents Study (APCAPS)[30] were used for the current cross-sectional study. The APCAPS is a prospective, intergenerational cohort study set in 29 villages (currently 28, as two villages have merged over time), which are located 30-80 km from Hyderabad in the Ranga Reddy district of Telangana, India. The APCAPS was established in 2003-05 as a long term follow-up of the cluster-randomised Hyderabad Nutrition Trial (HNT) (1987-90), in which all pregnant women and young children received (15 villages) or awaited (14 villages) a nutrition intervention.[23] In 2010-12, a third APCAPS survey wave included all available HNT index children, their parents and siblings (n=6944).[31 32]

Sample size and selection

All adults (18 years or older) from the third APCAPS survey wave, excluding pregnant women (n=80), were eligible for inclusion (n=6236). Participants who reported taking medication for outcome-related conditions were excluded to reduce the risk of biasing results, e.g. towards the null if participants with medically controlled

For peer review only - http://bmjopen.bmj.com/site/about/guidelines.xhtml

blood pressure sustained high levels of risk factors for hypertension. Participants were excluded from analyses of glucose if they reported fasting less than eight hours at the time of giving the blood (n=366) or currently taking medication (tablets or insulin) for diabetes (n=94). Participants were excluded from analysis of LDL if they reported fasting less than nine hours (n=364) or had triglyceride levels above 400 mg/dl[33] (n=92). Participants who reported taking antihypertensive medication were excluded from analyses of SBP (n=197) (Fig. 1). One village was significantly larger and had a considerably higher NTLI value than the other villages and its participants were excluded (n=297) to avoid overreliance of results from this outlier village.

Fig. 1 Sampling and participant flow

BMI – body mass index; FPG – fasting plasma glucose; HNT – Hyderabad Nutrition Trial; LDL – low-density lipoprotein; SBP – systolic blood pressure

* The Hyderabad Nutrition Trial (1987-1990) included 15 intervention and 14 control villages from the Integrated Child Development Services Scheme (stepped-wedge cluster randomised nutrition supplementation trial)

Data

Determining urbanisation levels of APCAPS villages

Aerial tracing of Bing satellite imagery, using Open Street Map software and GPS-based ground surveying during 2012-13, defined APCAPS village boundaries. The village boundary data were overlaid with the NTLI grids from 2011 (technical issues with satellite sensors prevented the use of NTLI data from 2012).[29] Twenty-four villages were intersected by two or more pixels, which in some cases had different NTLI values. Therefore, to obtain one NTLI value (urbanisation level) per village, we used a super-resolution method to resample the NTLI images to a higher-resolution, before summing the pixels intersecting with the village boundary to produce an overall village level DN. This ensured giving the appropriate weighting to each of the pixel values present over each village. Finally the data were calibrated using the ridgeline sampling regression methods[29] and oversaturation of light was removed manually. Since the first survey wave in 2003-5, the 27 included APCAPS villages had changed from rural agricultural communities with similar NTLI levels to represent a continuum of NTLI levels with growing differentiation at higher levels.

For peer review only - http://bmjopen.bmj.com/site/about/guidelines.xhtml

Selection of outcomes, CVD risk factors

We pre-specified the four outcomes, BMI, SBP, LDL and FPG, from a number of CVD risk factors available in the APCAPS, due to their well-established strong relationships with CVDs and their modifiable nature.[33 34] Continuous outcomes were selected for analysis to enable exploration of linear relationships between increasing level of NTLI and the CVD risk factors.

Health and socio-demographic data

Trained field staff collected data on health and socio-demographic information in the local language, Telugu, using semi-structured questionnaires. Trained field staff and medical doctors performed the clinical assessments in local clinics and at the National Institute of Nutrition in Hyderabad. Weight was measured twice to the nearest 0.1 kg with digital weight scales (Seca Leicester 899; Chasmors Ltd, London, United Kingdom (UK)), while wearing light clothing and no shoes. Standing height was measured twice to the nearest 0.1 cm with a portable stadiometer (Seca Leicester height measure; Chasmors Ltd, London, UK). Blood pressure was measured three times at the right arm in the sitting position using a digital device and appropriate sized cuff (Omron hem 7300; Matsusaka Co., Japan).

Biochemical assays

Venous fasting (eight to 12 hours) blood samples were collected, separated within 30 minutes, and stored locally at -20°C. Biochemical assays were performed using the Cobas311 auto-analyser at the Genetics and Biochemistry Laboratory at The South Asia Network for Chronic Diseases of the Public Health Foundation of India, New Delhi. Plasma glucose was assessed on the day of sampling by enzymatic Glucose Oxidase/Peroxidase-4-Aminophenazone-pPenol (GOD-PAP) method (Randox Laboratories; London, UK). Serum total cholesterol and triglycerides were assessed by enzymatic methods using Cholesterol Oxidase-Peroxidase-4-Aminophenazone-Phenol (CHOD-PAP) and Glycerol Phosphate Oxidase-Peroxidase-4-Aminophenazone-Phenol (GPO-PAP) (Roche Diagnostics GmbH; Mannheim, Germany). High-density lipoprotein (used for derivation of LDL) was derived by direct method using Polyethylene Glycol (PEG) modified Cholesterol Esterase, Cholesterol Oxidase and Dextran Sulphate (Roche Diagnostics GmbH; Mannheim, Germany). Low-density lipoprotein level was derived indirectly using the Fridewald-Fredrickson formula: LDL=total cholesterol–HDL–triglycerides/5 (with all measures in mg/dl).[33 35]

For peer review only - http://bmjopen.bmj.com/site/about/guidelines.xhtml

Quality assurance

Trained field staff and medical doctors followed standardised procedures and anthropometric equipment were calibrated at the start of every clinic. Reproducibility of clinical measurements were evaluated by repeating measures on a 5% random subsample after one to three weeks. The consistency was high for all measures with intra-class correlation coefficient for anthropometric measurements >0.98; blood pressure >0.85; and biochemical assays >0.94.[36] The quality of biochemical assays were monitored by the Cardiac Biochemistry laboratory at the All India Institute of Medical Sciences, which participates in the UK National External Quality Assessment Programme and the External Quality Assessment Scheme of Randox International.

Patient and Public Involvement

No patient involved

Statistical methods

The two weight and height measurements were averaged and BMI calculated for the analyses as weight (kg)/height (m)^2. The last two measurements of SBP were averaged for the analysis. Variables were assessed for outliers, cleaned accordingly, and examined for normality. Night-time light intensity data were non-normally distributed and log-transformed for analysis. To preserve study power, we allowed sample sizes to vary by outcomes. Mixed effects linear regression models with clustering by household and village were fitted in all analyses to account for the hierarchical nature of the data and the sampling strategy. Night-time light intensity (log transformed), BMI, SBP, LDL, FPG, age, and room temperature at clinical assessment (the latter for analysis of SBP) were included as continuous variables. Gender (women and men), caste (General caste, Scheduled caste, Scheduled tribe, Other backward class, and other), religion (Muslim, Hindu, Christian, and other), current marital status (married and unmarried), season of survey and season of birth (summer (March-May), South West monsoon (June-October), and winter (November-February)) were included as categorical variables. Study characteristics were presented by thirds of ranked NTLI, referred to as low, medium, and high levels. We evaluated potential confounders for each outcome using the double-selection method.[37] One final set of covariates, identified as potential confounders for at least one exposure-outcome relationship, were used in analyses of all four outcomes. Level of urbanisation may affect CVD risk profiles of different genders and age groups differently due to e.g. gender inequality, employment patterns, and readiness to adopt new lifestyles and behavious.[23 38-40] We therefore tested for interaction by gender and age. We did not consider socio-demographic factors such as education, occupation, and socio-economic status as potential confounders or effect modifiers

For peer review only - http://bmjopen.bmj.com/site/about/guidelines.xhtml

due to their importance on the causal pathway from urbanisation level to CVD risk factors. A sensitivity analysis among HNT index children (n=1245 (21% of overall sample); intervention n=622, control n=623) did not indicate confounding or interaction by intervention status. Results of a supplementary analysis for room temperature-adjusted SBP were presented separately, due to a large proportion of missing data (Supplementary Fig. 3 and Supplementary Tab. 2-3). A sensitivity analysis was performed to evaluate whether borderline outlier villages (with higher or lower observed mean outcomes than the majority of villages) influenced regression estimates considerably. Differences between included and excluded participants were examined with Chi square, and linear, and logistic mixed effects models with clustering by village and household. All analyses were performed in STATA 14 (StataCorp, College Station, TX, USA).

For peer review only

RESULTS

A total of 6236 non-pregnant adults were eligible for inclusion in the current study (90% of the third APCAPS survey wave). After removing extremes and participants with missing outcome data for specific analyses, the final samples came to 5924 participants for BMI; 5752 participants for SBP; 5287 participants for LDL; and 5328 participants for FPG (Fig. 1). Approximately half of participants were women (Tab. 1). The overall median age was 32 years (interquartile range (IQR) 24, 48), with women on average older than men. Men and women most frequently identified with other backward caste and Hindu religion. Women were more likely to be married (76%) than men (59%). Village-level NTLI ranged from 61.7 (equivalent to 4.1 on the log scale) to 1081.1 (equivalent to 7.0 on the log scale). The distribution of socio-demographic characteristics were similar across low, medium and high level of NTLI. None of the included (non-medicated) participants reported ever being diagnosed with heart disease, including stroke.

Tab. 1 Participant characteristics of APCAPS adults from 27 villages, 2010-12 (n=5932)

	**NTLI[a], women (n=2753 (46 %))**			**NTLI[a], men (n=3179 (54 %))**		
	**Low**	**Medium**	**High**	**Low**	**Medium**	**High**
**Night-time light intensity (NTLI), median (IQR)**	138.2 (121, 149)	202.4 (185, 273)	492.8 (485, 753)	138.2 (129, 149)	202.4 (185, 273)	492.8 (396, 753)
**Age, median (IQR)**	40 (25, 46)	40 (25, 46)	38 (25, 45)	28 (23, 51)	29 (24, 50)	28 (23, 49)
**Age group, n (%)**						
<30 years	299 (38.7)	372 (38.2)	416 (41.3)	489 (54.5)	595 (52.7)	636 (55.2)

	NTLI[a], women (n=2753 (46 %))			NTLI[a], men (n=3179 (54 %))		
	Low	Medium	High	Low	Medium	High
30-39 years	85 (11.0)	106 (10.9)	134 (13.3)	57 (6.4)	74 (6.6)	91 (7.9)
40-49 years	261 (33.8)	348 (35.8)	348 (34.5)	98 (10.9)	151 (13.4)	154 (13.4)
50+ years	127 (16.5)	147 (15.1)	110 (10.9)	254 (28.3)	309 (27.4)	271 (23.5)
Total	772 (100)	973 (100)	1008 (100)	898 (100)	1129 (100)	1152 (100)
**Caste, n (%)**						
General caste	51 (6.6)	79 (9.2)	76 (7.5)	51 (5.7)	89 (8.9)	63 (5.5)
Schedule caste	277 (35.9)	375 (43.6)	292 (29.0)	331 (36.9)	428 (42.6)	335 (29.1)
Schedule tribe	9 (1.2)	3 (0.4)	5 (0.5)	11 (1.2)	3 (0.3)	7 (0.6)
Other backward	425 (55.1)	374 (43.5)	628 (62.3)	488 (54.3)	455 (45.3)	739 (64.2)
Other	9 (1.2)	29 (3.4)	7 (0.7)	17 (1.9)	30 (3.0)	8 (0.7)
Total	771 (100)	860 (100)	1008 (100)	898 (100)	1005 (100)	1152 (100)
**Religion, n (%)**						
Muslim	19 (2.5)	37 (4.3)	71 (7.0)	16 (1.8)	45 (4.5)	64 (5.6)
Hindu	742 (96.2)	786 (91.4)	919 (91.2)	875 (97.4)	931 (92.6)	1079 (93.7)
Christian	9 (1.2)	37 (4.3)	16 (1.6)	7 (0.8)	29 (2.9)	8 (0.7)
Other	1 (0.1)	0 (0)	2 (0.2)	0 (0)	0 (0)	1 (0.1)
Total	771 (100)	860 (100)	1008 (100)	898 (100)	1005 (100)	1152 (100)
**Marital status, n (%)**						
Not married	196 (25.4)	238 (24.5)	229 (22.7)	381 (42.4)	459 (40.7)	487 (42.3)
Married	576 (74.6)	734 (75.5)	779 (77.3)	517 (57.6)	669 (59.3)	665 (57.7)
Total	772 (100)	972 (100)	1008 (100)	898 (100)	1128 (100)	1152 (100)
**Hyderabad Nutrition Trial, n (%)**						
Control	643 (83.3)	826 (84.9)	835 (82.8)	667 (74.3)	852 (75.5)	865 (75.1)
Intervention	129 (16.7)	147 (15.1)	173 (17.2)	231 (25.7)	277 (24.5)	287 (24.9)
Total	772 (100)	973 (100)	1008 (100)	898 (100)	1129 (100)	1152 (100)
**Room temperature (^{0}C), mean (95% CI)**	28.7 (3.4)	30.2 (3.4)	30.1 (2.6)	28.3 (3.5)	29.8 (3.2)	29.6 (2.6)

CI – confidence interval; IQR – Inter quartile range; n – sample size; NTLI – night-time light intensity

[a]Characteristics are presented by thirds (low, medium and high level) of ranked NTLI

Model predicted mean BMI, SBP and LDL increased linearly with rising NTLI in crude and adjusted models (Fig. 2 and Tab. 2). Across the range of NTLI, the fully adjusted predicted mean increased 1.0 kg/m^2 (95% CI 0.01, 1.9, p=0.05) for BMI; and 4.2 mmHg (95% CI 1.0, 7.4, p=0.01) for SBP. The pattern was similar for LDL

For peer review only

For peer review only - http://bmjopen.bmj.com/site/about/guidelines.xhtml

with a fully adjusted predicted mean increase across the range of NTLI of 0.3 mmol/l (95% CI -0.01, 0.7), although the evidence was weak from crude (p=0.06) and fully adjusted models (p=0.06). There were no linear associations of NTLI with FPG (fully adjusted mean difference across the range of NTLI: -0.01 mmol/l (95% CI -0.4, 0.4, p=0.97).

Fig. 2 Crude associations of night-time light intensity with CVD risk factors among APCAPS adults, 2010-12 (n=5937)

APCAPS – Andhra Pradesh Children and Parents Study; BMI – body mass index; FPG – fasting plasma glucose; LDL – low-density lipoprotein; NTLI – night-time light intensity; SBP – systolic blood pressure

Model predicted means were derived from multilevel linear regression with clustering by household and village, using individual-level outcome data

Marker size proportional to village size

For peer review only

231 **Tab. 2 Crude and adjusted associations of NTLI with CVD risk factors among APCAPS adults, 2010-12 (n=5937)**

					Model predicted age and gender adjusted mean change (95% CI) with increasing NTLI[c]			Model predicted fully adjusted mean change (95% CI) with increasing NTLI[c, d]		
	n	Model predicted crude mean (95% CI) at the lowest NTLI (61.7 (4.1 on the log scale))	Model predicted crude mean (95% CI) at the highest NTLI (1081.1 (7.0 on the log scale))	p-value	n	β (95% CI)	p-value	n	β (95% CI)	p-value
SBP (mmHg)[a]	5752	117.2 (115.1, 119.4)	121.6 (119.2, 123.9)	0.04	5747	1.8 (0.4, 3.2)	0.01	5514	1.5 (0.3, 2.6)	0.01
BMI (kg/m2)	5924	20.3 (19.8, 20.8)	21.5 (21, 22.1)	0.01	5920	0.4 (0.1, 0.8)	0.01	5682	0.3 (0.03, 0.7)	0.05
Fasting serum LDL (mmol/l)	5287	2.4 (2.2, 2.6)	2.7 (2.5, 2.9)	0.06	5285	0.1 (0.01, 0.2)	0.04	5051	0.1 (-0.003, 0.2)	0.06
Fasting plasma glucose (mmol/l)[b]	5328	5.1 (5.0, 5.3)	5.2 (5.0, 5.4)	0.61	5326	0.04 (-0.1, 0.2)	0.52	5094	-0.002 (-0.1, 0.1)	0.97

232 BMI – body mass index; CI - confidence interval; LDL – low-density lipoprotein; n – sample size; NTLI – night-time light intensity; SBP – systolic blood pressure; β – beta-
233 coefficient

234 Model predicted means (95% CIs), β-coefficients (95% CIs) and p-values were obtained from multilevel linear regression models with clustering by household and village
235 (using individual-level outcome data)

236 Participants were excluded if medicated for hypertension[a] or diabetes[b]

237 [c]Mean change per whole number increase in log transformed NTLI

238 [d]Adjusted for age, gender, caste, religion, marital status and survey season

For peer review only - http://bmjopen.bmj.com/site/about/guidelines.xhtml

Model predicted mean SBP with increasing NTLI was on average 6.1 mmHg (95% CI 5.42, 6.9, p<0.001) higher among men than women, whereas levels of BMI, LDL and FPG did not differ by gender. However, there was no statistical evidence of gender modifying any of the associations between NTLI and the CVD risk factors. There was strong evidence of age modifying the associations of NTLI with BMI (p<0.001) and SBP (p<0.001), but not LDL, or FPG. Night-time light intensity was not associated with BMI or SBP in the younger age groups (up to 39 years), whereas the evidence and magnitudes of associations from both crude and fully adjusted models were stronger in older age groups (from 40 years) than predicted in the overall analysis (Supplementary Fig. 1 and Supplementary Tab. 1). Supplementary Fig. 2 illustrates at which levels of NTLI the fully adjusted models predicted the greatest probability of associations differing by age group.

Overall and age-specific results of a supplementary analysis for SBP adjusted for room temperature at clinical assessment (n=3160) were similar to the main and age-specific SBP analyses (Supplementary Tab. 2-3 and Supplementary Fig. 3).

Participants, who were excluded from the main analysis due to missing data, medication use or high triglyceride levels differed in some respects from the analysed participants. Excluded individuals were on average older, less likely to attend clinical assessment/interview in the summer, and had higher levels of the four CVD risk factors than included individuals.

Sensitivity analysis

A sensitivity analysis removing three borderline outlier villages from analysis of BMI, SBP and FPG (none identified for LDL) were consistent with the overall and age-stratified main analyses (Supplementary Fig. 4-5 and Supplementary Tab. 4-5). However, results for SBP (not adjusted for room temperature) were not statistically significant at the 95% level. Supplementary Fig. 6 illustrates at which levels of NTLI the fully adjusted sensitivity analysis predicted the greatest probability of differences in associations of NTLI and CVD risk factors by age group.

DISCUSSION

Among 6236 participants, model predicted mean BMI, SBP and serum fasting LDL, but not FPG, increased linearly with rising level NTLI after controlling for confounders. We expected younger individuals to more readily adopt new lifestyles and behaviours associated with urbanisation and CVD risk factors than older

For peer review only - http://bmjopen.bmj.com/site/about/guidelines.xhtml

individuals.[9 39] This was however not apparent from the data. The observed stronger associations of NTLI with BMI and SBP in older age groups were likely attributable to advancing age.[33] Gender did not modify associations.

Comparison with studies using night-time light intensity

Few studies have used NTLI data to explore relationships of urbanisation and CVD risk factors. A Korean cross-sectional study (n=8526)[41] and a multi-country study (n=130 counties),[42] investigated associations of DMSP NTLI with BMI. Consistent with our results on BMI, increasing level of NTLI was associated with rising levels of overweight and obesity in both studies. A cross-sectional study from India, which analysed NTLI data from NASA's Visible Infrared Imaging Radiometer Suite[43], corroborates our adjusted results for SBP, although the included set of covariates differed between our studies. We did not find any studies on relationships between NTLI and LDL or FPG.

Comparison with studies using multi-component urbanisation scores

Our findings on BMI and SBP agree with cross-sectional studies from South India[15] (n=3705) and Sri Lanka (n=4485),[18] which used multi-component scores to estimate urbanisation. Although we cannot compare our results directly to studies that use urbanisation scores (as opposed to NTLI) the direction of effects are consistent with our findings and support evidence of an association. We found no studies exploring relationships between urbanisation score and FPG, whereas findings from Sri Lanka[18] and China[44] suggest a greater likelihood of diabetes as urbanisation score increased. Restricting our analysis to non-diabetics may have contributed to the null results for FPG, particularly as excluded individuals were on average older and had higher BMI.

Comparison with rural-urban and migrant studies

Measuring urbanisation is challenging, particularly in settings where population and environment data are sparse.[22] As a result, relationships of urbanisation and CVD risk factors are often inferred from rural-urban comparisons and migrant studies. Several of these studies focus on or include Asian populations. An Indian migration study of 3537 sibling pairs suggests that mean BMI is lower among rural than migrant and urban populations. Patterns were similar for mean SBP, LDL, and FPG among men but not women.[40] In a different analysis of the same study, cumulative exposure to urban environments additionally increased mean BMI of both genders as well as SBP and fasting glucose of men, while LDL remained unaffected.[13] Systolic blood pressure (as the only outcome) was on average higher among men than women in our sample, however gender did not modify associations. A cross-sectional study from Southern India suggests that mean BMI, SBP, and the

For peer review only - http://bmjopen.bmj.com/site/about/guidelines.xhtml

prevalence of diabetes were lower among peri-urban residents of both genders than among town and city dwellers.[45] Urban versus rural residence was also associated with higher prevalence of diabetes[44] and mean LDL in China[46] after adjusting for important confounders. Two large studies from 56[8] and 36[47] countries (n=878000 and 148579 respectively) across Africa, the Middle East, Asia, Americas, the Caribbean and USA suggest that overweight (BMI ≥25 kg/m^2) is considerably more common among urban than rural women, particularly in lower income countries.[8] In a subsample from India (n=7608), the prevalence of overweight was almost five fold among urban (26.4 %) compared to rural women (5.6 %).[47]

Strengths and limitations

A major contribution of our study to the existing evidence was exploring CVD risk factors in a transitioning population, i.e. one that has experienced rapid and uneven urbanisation over time and thus represented a continuum of urbanisation levels at the time of data collection. The village-level NTLI has been validated as a standardised and comparable proxy of urbanisation level, reflecting extent of build-up area, population density, economic activity, and energy consumption world-wide.[24-27] It may further be a more objective measure than the survey based data used for multiple-component urbanisation scales, which are at risk of both interviewer and reporting biases. The NTLI data have gained popularity for exploring associations of urbanisation level and artificial light at night with human health, particularly cancer,[48-56] overweight and obesity.[41 42 57] The NTLI time series has further been suggested a valid proxy for urbanisation dynamics over time,[24 27] especially in rapidly developing countries with high urban growth rates such as China, India, and Brazil.[27] Recent efforts to calibrate the NTLI time series has shown great promise for studying urbanisation dynamics in these settings[29] and offer a unique resource for health research worldwide. We calibrated the NTLI data using a robust and reliable semiautomatic method to reduce systematic bias from satellite discrepancies and enable more efficient and precise comparisons of results between studies from different regions and time points.[29] However, the semiautomatic method could introduce new bias from manual processing.[29] The overall agreement of our result with existing evidence (using different measures of urbanisation level) supports the utility of NTLI to predict urbanisation-related changes in CVD risk factors. The large sample size and use of hierarchical statistical methods should reduce overestimation of the magnitude of associations from risk factor clustering by sampling units (villages and households). The comparisons of excluded and included participants did however warranted some caution of generalising the results to the general population. Trained field and clinical staff followed standardised protocols and collected information in the local language. A comprehensive list of factors were measured and explored as potential confounders and effect modifiers in our study, however, some residual

For peer review only - http://bmjopen.bmj.com/site/about/guidelines.xhtml

325 confounding from unmeasured factors may remain. Data for a number of covariates were self-reported, and some recall or reporting bias cannot be rule out. The cross-sectional design of the present study further prevented us from inferring causality of the results as well as from exploring the relative contribution of different urbanisation elements to CVD risk.

329 **Possible mechanisms**

330 There are a number of plausible pathways through which urbanisation may deteriorate cardiovascular health. Physical and build environments change with urbanisation and in turn influence the level of exposure to noise and air pollution, green spaces, infrastructure.[22 43 58 59] Urbanisation is also associated with changes in occupation patterns and socio-economic status[15 58]; social norms, cohesion, and support; as well as personal tastes and preferences.[22 43 59] Together with increased availability, access and advertising of commodities, these transitions promote lifestyle changes that are strongly associated with CVD risk, e.g. 'nutrition transition' towards diets high in fat, sugar and salt as well as processed and convenience foods; physical inactivity during work, leisure time, transport and household chores; tobacco use and alcohol consumption.[9 18 22 58 60] Cumulative exposure to urban environments additionally appears to increase CVD risk.[13 17] Recent studies further suggest that increased exposure to artificial light at night observed with increasing urbanisation level supresses melatonin production, disrupts circadian rhythm, and leads to physiological and behavioural changes associated with CVD.[42] A number of studies focusing on artificial light at night (other than NTLI) without accounting for urbanisation support this mechanism.[49 61-66] At the same time, greater level of urbanisation is also associated with number of changes that may improve CVD health. Greater level of urbanisation is for example associated with greater levels and higher quality of education; improved opportunities for paid work, particularly for women; higher socio-economic status and living conditions; better access to health, social services and safe food, which have great potential to improve cardiovascular health.[67-72] Although these pathways have been studies extensively, no study has to our knowledge attempted to quantify their relative harmful and beneficial contributions.

349 **Implications**

350 Our findings indicate that the harmful impacts of increasing urbanisation level on SBP and BMI, well-established leading modifiable risk factors for CVDs, outweigh any beneficial impacts even with moderate increases in urbanisation level. These findings suggest an opportunity for policy makers and urban planners to help curb expected increases in urbanisation-related CVDs by addressing underlying and immediate risk factors

For peer review only - http://bmjopen.bmj.com/site/about/guidelines.xhtml

for elevated SBP and BMI during early stages of urbanisation. We will however need to understand better and quantify the implicated pathways in different settings in order to identify cost-effective prevention strategies.

CONCLUSION

We found that the NTLI data, a novel measure of urbanisation, is a useful tool to predict changes in important urbanisation-related CVD risk factors where objective data on urbanisation factors are not readily available. The observed consistent associations of moderate increases in urbanisation level (measured by NTLI) with CVD risk factors provide an important early warning for the potential progression of the CVD burden in South India with continued urbanisation. To curb expected large public health impacts from continued and rapid urbanisation in India, further research is warranted to identify the most important mechanisms. To enable targeting of cost-effective prevention strategies, we suggest that future studies focus on transitioning populations and use causal methods to better understand potentially important pathways.

For peer review only

ABBREVIATIONS

APCAPS	Andhra Pradesh Children and Parents Study
BMI	body mass index
CI	confidence interval
CVD	cardiovascular disease
DMSP-OLS	Defence Meteorological Satellite Programme's Operational Linescan System
DN	digital number
FPG	fasting plasma glucose
HNT	Hyderabad Nutrition Trial
IQR	inter quartile range
LDL	low-density lipoprotein
LMIC	low- and middle-income country
LSHTM	London School of Hygiene and Tropical Medicine

For peer review only - http://bmjopen.bmj.com/site/about/guidelines.xhtml

n	sample size
NTLI	night-time light intensity
SBP	systolic blood pressure
UK	United Kingdom
β	Beta-coefficient

DECLARATIONS

Ethics

Ethics approvals were obtained from Public Health Foundation of India, New Delhi, India; National Institute of Nutrition, Hyderabad, India; and the London School of Hygiene and Tropical Medicine (LSHTM), London, UK. Approvals were obtained from all village heads and their committees. Study participants provided written informed consent or a witnessed thumbprint if illiterate prior to study start.

Consent for publication

Study participants provided written informed consent, or a witnessed thumbprint if illiterate, for the use and publication of their anonymised data in accordance with the study objectives.

Availability of data and materials

The dataset analysed during the current study is available from the APCAPS (http://apcaps.lshtm.ac.uk/)[30] upon reasonable request.

Competing interests

The authors declare that they have no competing interests

Role of the funding source

This work was supported by Wellcome Trust (www.wellcome.ac.uk/) Grant number 083707 and the Bloomsbury PhD Studentship (http://www.bloomsbury.ac.uk/) through LSHTM, London, UK

Author contributions

All authors contributed substantially to study conception and manuscript revision; and approved the final manuscript for publication. TBS and JG cleaned and managed the Andhra Pradesh Children and Parents Study

For peer review only

For peer review only - http://bmjopen.bmj.com/site/about/guidelines.xhtml

(APCAPS) data in collaboration with the APCAPS team. RW extracted, calibrated and managed the night-time light intensity data; JG advised on statistical methods. TBS conducted the literature search, data analysis and interpretation, and drafted the manuscript; SK is the director of the APCAPS.

Acknowledgements

We would like to thank the APCAPS team and support staff, in particular (in alphabetical order by surname) Srivalli Addanki, Christopher Baker, Santhi Bhogadi, Naveen Chittaluri, Ruby Gupta, **Bharati Kulkarni**, Poppy Mallinson, Sandeep Masapeta, Subhash Masapeta, and Chitra Sharma.

For peer review only

REFERENCES

1. India State-level Disease Burden Initiative Collaborators. Nations within a nation: variations in epidemiological transition across the states of India, 1990-2016 in the Global Burden of Disease Study. *Lancet* 2017 doi: 10.1016/s0140-6736(17)32804-0 [published Online First: 2017/11/19]
2. Prabhakaran D, Jeemon P, Roy A. Cardiovascular Diseases in India: Current Epidemiology and Future Directions. *Circulation* 2016;133(16):1605-20. doi: 10.1161/circulationaha.114.008729 [published Online First: 2016/05/05]
3. Patel V, Chatterji S, Chisholm D, et al. Chronic diseases and injuries in India. *Lancet* 2011;377(9763):413-28. doi: 10.1016/s0140-6736(10)61188-9 [published Online First: 2011/01/14]
4. Yusuf S, Rangarajan S, Teo K, et al. Cardiovascular risk and events in 17 low-, middle-, and high-income countries. *The New England journal of medicine* 2014;371(9):818-27. doi: 10.1056/NEJMoa1311890 [published Online First: 2014/08/28]
5. Gupta R, Joshi P, Mohan V, et al. Epidemiology and causation of coronary heart disease and stroke in India. *Heart (British Cardiac Society)* 2008;94(1):16-26. doi: 10.1136/hrt.2007.132951 [published Online First: 2007/12/18]
6. Sharma M, Ganguly NK. Premature coronary artery disease in Indians and its associated risk factors. *Vascular health and risk management* 2005;1(3):217-25. [published Online First: 2007/02/27]
7. Prabhakaran D, Singh K. Premature coronary heart disease risk factors & reducing the CHD burden in India. *The Indian journal of medical research* 2011;134:8-9. [published Online First: 2011/08/03]
8. Goryakin Y, Suhrcke M. Economic development, urbanization, technological change and overweight: what do we learn from 244 Demographic and Health Surveys? *Econ Hum Biol* 2014;14:109-27. doi: 10.1016/j.ehb.2013.11.003 [published Online First: 2014/01/25]
9. Popkin BM, Adair LS, Ng SW. Global nutrition transition and the pandemic of obesity in developing countries. *Nutr Rev* 2012;70(1):3-21. doi: 10.1111/j.1753-4887.2011.00456.x [published Online First: 2012/01/10]
10. Allender S, Foster C, Hutchinson L, et al. Quantification of urbanization in relation to chronic diseases in developing countries: a systematic review. *Journal of urban health : bulletin of the New York Academy of Medicine* 2008;85(6):938-51. doi: 10.1007/s11524-008-9325-4 [published Online First: 2008/10/22]

For peer review only - http://bmjopen.bmj.com/site/about/guidelines.xhtml

11. Vlahov D, Galea S. Urbanization, urbanicity, and health. *Journal of urban health : bulletin of the New York Academy of Medicine* 2002;79(4 Suppl 1):S1-s12. [published Online First: 2002/12/11]

12. Moore M, Gould P, Keary BS. Global urbanization and impact on health. *International journal of hygiene and environmental health* 2003;206(4):269-78. doi: https://doi.org/10.1078/1438-4639-00223

13. Kinra S, Andersen E, Ben-Shlomo Y, et al. Association between urban life-years and cardiometabolic risk: the Indian migration study. *American journal of epidemiology* 2011;174(2):154-64. doi: 10.1093/aje/kwr053 [published Online First: 2011/05/31]

14. Bowen L, Ebrahim S, De Stavola B, et al. Dietary intake and rural-urban migration in India: a cross-sectional study. *PLoS One* 2011;6(6):e14822. doi: 10.1371/journal.pone.0014822 [published Online First: 2011/07/07]

15. Allender S, Lacey B, Webster P, et al. Level of urbanization and noncommunicable disease risk factors in Tamil Nadu, India. *Bull World Health Organ* 2010;88(4):297-304. doi: 10.2471/blt.09.065847 [published Online First: 2010/05/01]

16. Subramanian SV, Smith GD. Patterns, distribution, and determinants of under- and overnutrition: a population-based study of women in India. *The American journal of clinical nutrition* 2006;84(3):633-40. [published Online First: 2006/09/09]

17. Sobngwi E, Mbanya JC, Unwin NC, et al. Exposure over the life course to an urban environment and its relation with obesity, diabetes, and hypertension in rural and urban Cameroon. *International journal of epidemiology* 2004;33(4):769-76. doi: 10.1093/ije/dyh044 [published Online First: 2004/05/29]

18. Allender S, Wickramasinghe K, Goldacre M, et al. Quantifying urbanization as a risk factor for noncommunicable disease. *Journal of urban health : bulletin of the New York Academy of Medicine* 2011;88(5):906-18. doi: 10.1007/s11524-011-9586-1 [published Online First: 2011/06/04]

19. Torun B, Stein AD, Schroeder D, et al. Rural-to-urban migration and cardiovascular disease risk factors in young Guatemalan adults. *International journal of epidemiology* 2002;31(1):218-26. [published Online First: 2002/03/27]

20. Poulter NR, Khaw KT, Hopwood BE, et al. The Kenyan Luo migration study: observations on the initiation of a rise in blood pressure. *BMJ (Clinical research ed)* 1990;300(6730):967-72. [published Online First: 1990/04/14]

21. Al-Moosa S, Allin S, Jemiai N, et al. Diabetes and urbanization in the Omani population: an analysis of national survey data. *Population health metrics* 2006;4:5. doi: 10.1186/1478-7954-4-5 [published Online First: 2006/04/26]

22. Dahly DL, Adair LS. Quantifying the urban environment: a scale measure of urbanicity outperforms the urban-rural dichotomy. *Social science & medicine (1982)* 2007;64(7):1407-19. doi: 10.1016/j.socscimed.2006.11.019

23. Knowledge Network on Urban Settings. Our cities, our health, our future. Kobe City, Japan: World Health Organization, 2008.

24. Yi K, Tani H, Li Q, et al. Mapping and evaluating the urbanization process in Northeast China using DMSP/OLS nighttime light data. *Sensors (Basel, Switzerland)* 2014;14(2):3207-26. doi: 10.3390/s140203207 [published Online First: 2014/02/21]

25. Ma T, Zhou C, Pei T, et al. Quantitative estimation of urbanization dynamics using time series of DMSP/OLS nighttime light data: A comparative case study from China's cities. *Remote Sensing of Environment* 2012;124(Supplement C):99-107. doi: https://doi.org/10.1016/j.rse.2012.04.018

26. Imhoff M, Lawrence W, Stutzer D, et al. A technique for using composite DMSP/OLS “City Lights” satellite data to map urban area. *Remote Sensing of Environment* 1997;61(3):361-70. doi: https://doi.org/10.1016/S0034-4257(97)00046-1

For peer review only - http://bmjopen.bmj.com/site/about/guidelines.xhtml

27. Zhang Q, Seto KC. Mapping urbanization dynamics at regional and global scales using multi-temporal DMSP/OLS nighttime light data. *Remote Sensing of Environment* 2011;115(9):2320-29. doi: https://doi.org/10.1016/j.rse.2011.04.032
28. Elvidge CD, Imhoff ML, Baugh KE, et al. Nighttime lights of the world: 1994-95 *ISPRS Journal of Photogrammetry and Remote Sensing* 2001;56:81-99.
29. Zhang Q, Pandey B, Seto KC. A Robust Method to Generate a Consistent Time Series From DMSP/OLS Nighttime Light Data. *IEEE Transactions on geoscience and remote sensisng* 2016 doi: 10.1109/TGRS.2016.2572724
30. Andhra Pradesh Children and Parents Study (APCAPS). Andhra Pradesh Children and Parents Study: London School of Hygiene and Tropical Medicine; 2018 [Available from: http://apcaps.lshtm.ac.uk/ accessed 12 July 2018.
31. Kinra S, Radha Krishna K, Kuper H, et al. Cohort Profile: Andhra Pradesh Children and Parents Study (APCAPS). *International journal of epidemiology* 2013 doi: 10.1093/ije/dyt128 [published Online First: 2013/09/11]
32. Kinra S, Rameshwar Sarma KV, Ghafoorunissa, et al. Effect of integration of supplemental nutrition with public health programmes in pregnancy and early childhood on cardiovascular risk in rural Indian adolescents: long term follow-up of Hyderabad nutrition trial. *BMJ (Clinical research ed)* 2008;337:a605. doi: 10.1136/bmj.a605 [published Online First: 2008/07/29]
33. National Cholesterol Education Program, National Heart L, and Blood Institute,, National Institutes of Health. Third Report of the National Cholesterol Education Program (NCEP) Expert Panel on Detection, Evaluation, and Treatment of High Blood Cholesterol in Adults (Adult Treatment Panel III). NIH Publication, 2002.
34. GBD 2015 Risk Factors Collaborators. Global, regional, and national comparative risk assessment of 79 behavioural, environmental and occupational, and metabolic risks or clusters of risks, 1990-2015: a systematic analysis for the Global Burden of Disease Study 2015. *Lancet* 2016;388(10053):1659-724. doi: 10.1016/s0140-6736(16)31679-8 [published Online First: 2016/10/14]
35. Friedewald WT, Levy RI, Fredrickson DS. Estimation of the concentration of low-density lipoprotein cholesterol in plasma, without use of the preparative ultracentrifuge. *Clinical chemistry* 1972;18(6):499-502. [published Online First: 1972/06/01]
36. Kinra S, Johnson M, Kulkarni B, et al. Socio-economic position and cardiovascular risk in rural indian adolescents: evidence from the Andhra Pradesh children and parents study (APCAPS). *Public health* 2014;128(9):852-9. doi: 10.1016/j.puhe.2014.06.005
37. Belloni A, Chernozhukov V, Hansen C. Inference on Treatment Effects after Selection among High-Dimensional Controls. *The Review of Economic Studies* 2014;81(2):608-50. doi: 10.1093/restud/rdt044
38. Tacoli C. Urbanization and emerging population issues working paper 7. Urbanization, gender and urban poverty: paid work and unpaid carework in the city: United Nations Population Fund, International Institute for Environment and Development,, 2012:48.
39. Poobalan A, Aucott L. Obesity Among Young Adults in Developing Countries: A Systematic Overview. *Current Obesity Reports* 2016;5:2-13. doi: 10.1007/s13679-016-0187-x
40. Ebrahim S, Kinra S, Bowen L, et al. The effect of rural-to-urban migration on obesity and diabetes in India: a cross-sectional study. *PLoS medicine* 2010;7(4):e1000268. doi: 10.1371/journal.pmed.1000268 [published Online First: 2010/05/04]
41. Koo YS, Song JY, Joo EY, et al. Outdoor artificial light at night, obesity, and sleep health: Cross-sectional analysis in the KoGES study. *Chronobiology international* 2016;33(3):301-14. doi: 10.3109/07420528.2016.1143480 [published Online First: 2016/03/08]
42. Rybnikova NA, Haim A, Portnov BA. Does artificial light-at-night exposure contribute to the worldwide obesity pandemic? *International journal of obesity (2005)* 2016;40(5):815-23. doi: 10.1038/ijo.2015.255 [published Online First: 2016/01/23]

For peer review only

43. Lane KJ, Stokes EC, Seto KC, et al. Associations between Greenness, Impervious Surface Area, and Nighttime Lights on Biomarkers of Vascular Aging in Chennai, India. *Environmental health perspectives* 2017;125(8):087003. doi: 10.1289/ehp541 [published Online First: 2017/09/09]
44. Attard SM, Herring AH, Mayer-Davis EJ, et al. Multilevel examination of diabetes in modernising China: what elements of urbanisation are most associated with diabetes? *Diabetologia* 2012;55(12):3182-92. doi: 10.1007/s00125-012-2697-8 [published Online First: 2012/08/28]
45. Ramachandran A, Mary S, Yamuna A, et al. High prevalence of diabetes and cardiovascular risk factors associated with urbanization in India. *Diabetes care* 2008;31(5):893-8. doi: 10.2337/dc07-1207 [published Online First: 2008/03/04]
46. He J, Klag MJ, Wu Z, et al. Effect of migration and related environmental changes on serum lipid levels in southwestern Chinese men. *American journal of epidemiology* 1996;144(9):839-48. [published Online First: 1996/11/01]
47. Mendez MA, Monteiro CA, Popkin BM. Overweight exceeds underweight among women in most developing countries. *The American journal of clinical nutrition* 2005;81(3):714-21. [published Online First: 2005/03/10]
48. Kloog I, Haim A, Stevens RG, et al. Global co-distribution of light at night (LAN) and cancers of prostate, colon, and lung in men. *Chronobiology international* 2009;26(1):108-25. doi: 10.1080/07420520802694020 [published Online First: 2009/01/15]
49. Cho Y, Ryu SH, Lee BR, et al. Effects of artificial light at night on human health: A literature review of observational and experimental studies applied to exposure assessment. *Chronobiology international* 2015;32(9):1294-310. doi: 10.3109/07420528.2015.1073158 [published Online First: 2015/09/17]
50. Rybnikova NA, Haim A, Portnov BA. Is prostate cancer incidence worldwide linked to artificial light at night exposures? Review of earlier findings and analysis of current trends. *Archives of environmental & occupational health* 2017;72(2):111-22. doi: 10.1080/19338244.2016.1169980 [published Online First: 2016/04/01]
51. Kloog I, Haim A, Stevens RG, et al. Light at night co-distributes with incident breast but not lung cancer in the female population of Israel. *Chronobiology international* 2008;25(1):65-81. doi: 10.1080/07420520801921572 [published Online First: 2008/02/23]
52. Hurley S, Goldberg D, Nelson D, et al. Light at night and breast cancer risk among California teachers. *Epidemiology (Cambridge, Mass)* 2014;25(5):697-706. doi: 10.1097/ede.0000000000000137 [published Online First: 2014/07/26]
53. James P, Bertrand KA, Hart JE, et al. Outdoor Light at Night and Breast Cancer Incidence in the Nurses' Health Study II. *Environmental health perspectives* 2017;125(8):087010. doi: 10.1289/ehp935 [published Online First: 2017/09/09]
54. Kloog I, Stevens RG, Haim A, et al. Nighttime light level co-distributes with breast cancer incidence worldwide. *Cancer causes & control : CCC* 2010;21(12):2059-68. doi: 10.1007/s10552-010-9624-4 [published Online First: 2010/08/04]
55. Bauer SE, Wagner SE, Burch J, et al. A case-referent study: light at night and breast cancer risk in Georgia. *International Journal of Health Geographics* 2013;12:23. doi: 10.1186/1476-072x-12-23 [published Online First: 2013/04/19]
56. Rybnikova N, Haim A, Portnov BA. Artificial Light at Night (ALAN) and breast cancer incidence worldwide: A revisit of earlier findings with analysis of current trends. *Chronobiology international* 2015;32(6):757-73. doi: 10.3109/07420528.2015.1043369 [published Online First: 2015/06/24]
57. Touitou Y, Reinberg A, Touitou D. Association between light at night, melatonin secretion, sleep deprivation, and the internal clock: Health impacts and mechanisms of circadian disruption. *Life sciences* 2017;173:94-106. doi: 10.1016/j.lfs.2017.02.008 [published Online First: 2017/02/20]
58. Tacoli C. Urbainzation, gender and poverty. New York, USA: United Nations Population Fund, International Institute for Environment and Development, 2012.

For peer review only - http://bmjopen.bmj.com/site/about/guidelines.xhtml

59. de Vries S, van Dillen SM, Groenewegen PP, et al. Streetscape greenery and health: stress, social cohesion and physical activity as mediators. *Soc Sci Med* 2013;94:26-33. doi: 10.1016/j.socscimed.2013.06.030 [published Online First: 2013/08/13]
60. Shetty PS. Nutrition transition in India. *Public health nutrition* 2002;5(1A):175-82. doi: 10.1079/phn2001291 [published Online First: 2002/05/25]
61. Hurley S, Nelson DO, Garcia E, et al. A cross-sectional analysis of light at night, neighborhood sociodemographics and urinary 6-sulfatoxymelatonin concentrations: implications for the conduct of health studies. *International Journal of Health Geographics* 2013;12:39. doi: 10.1186/1476-072x-12-39
62. McFadden E, Jones ME, Schoemaker MJ, et al. The relationship between obesity and exposure to light at night: cross-sectional analyses of over 100,000 women in the Breakthrough Generations Study. *American journal of epidemiology* 2014;180(3):245-50. doi: 10.1093/aje/kwu117 [published Online First: 2014/05/31]
63. Gangwisch JE. Invited commentary: nighttime light exposure as a risk factor for obesity through disruption of circadian and circannual rhythms. *American journal of epidemiology* 2014;180(3):251-3. doi: 10.1093/aje/kwu119 [published Online First: 2014/05/31]
64. Stevens RG. Light-at-night, circadian disruption and breast cancer: assessment of existing evidence. *International journal of epidemiology* 2009;38(4):963-70. doi: 10.1093/ije/dyp178 [published Online First: 2009/04/22]
65. Obayashi K, Saeki K, Iwamoto J, et al. Association between light exposure at night and nighttime blood pressure in the elderly independent of nocturnal urinary melatonin excretion. *Chronobiology international* 2014;31(6):779-86. doi: 10.3109/07420528.2014.900501 [published Online First: 2014/03/29]
66. Obayashi K, Saeki K, Iwamoto J, et al. Effect of exposure to evening light on sleep initiation in the elderly: a longitudinal analysis for repeated measurements in home settings. *Chronobiology international* 2014;31(4):461-7. doi: 10.3109/07420528.2013.840647 [published Online First: 2013/10/24]
67. Ezzati M, Webster CJ, Doyle YG, et al. Cities for global health. 2018;363:k3794. doi: 10.1136/bmj.k3794 %J BMJ
68. World Health Organization. Urbanization and health 2019 [Available from: https://www.who.int/bulletin/volumes/88/4/10-010410/en/ accessed 16 July 2019.
69. Vlahov D, Freudenberg N, Proietti F, et al. A conceptual framework for organizing determinants of urban health. Thematic paper 1 for Knowledge Network on Urban Settings WHO Centre for Health Development, 2008.
70. Satterthwaite D, McGranahan G, Tacoli C. Urbanization and its implications for food and farming. *Philosophical transactions of the Royal Society of London Series B, Biological sciences* 2010;365(1554):2809-20. doi: 10.1098/rstb.2010.0136 [published Online First: 2010/08/18]
71. Estache A, Garsous G. The scope for an impact of infrastructure investments on jobs in developing countries International Finance Corporation, 2012.
72. Eckert S, Kohler S. Urbanization and health in developing countries: a systematic review. *World health & population* 2014;15(1):7-20. [published Online First: 2014/04/08]

FIGURE TITLES AND LEGENDS

Fig. 3 Sampling and participant flow of the current study

BMI – body mass index; FPG – fasting glucose; HNT – Hyderabad Nutrition Trial; LDL – low-density lipoprotein; SBP – systolic blood pressure

For peer review only - http://bmjopen.bmj.com/site/about/guidelines.xhtml

*The Hyderabad Nutrition Trial (1987-1990) included 15 intervention and 14 control villages from the Integrated Child Development Services Scheme (stepped-wedge cluster randomised nutrition supplementation trial)

Fig. 4 Crude associations of night-time light intensity with CVD risk factors among APCAPS adults, 2010-12 (n=5937)

APCAPS – Andhra Pradesh Children and Parents Study; BMI – body mass index; FPG – fasting plasma glucose; LDL – low-density lipoprotein; NTLI – night-time light intensity; SBP – systolic blood pressure

Model predicted means were derived from multilevel linear regression with clustering by household and village, using individual-level outcome data

Marker size proportional to village size

For peer review only - http://bmjopen.bmj.com/site/about/guidelines.xhtml

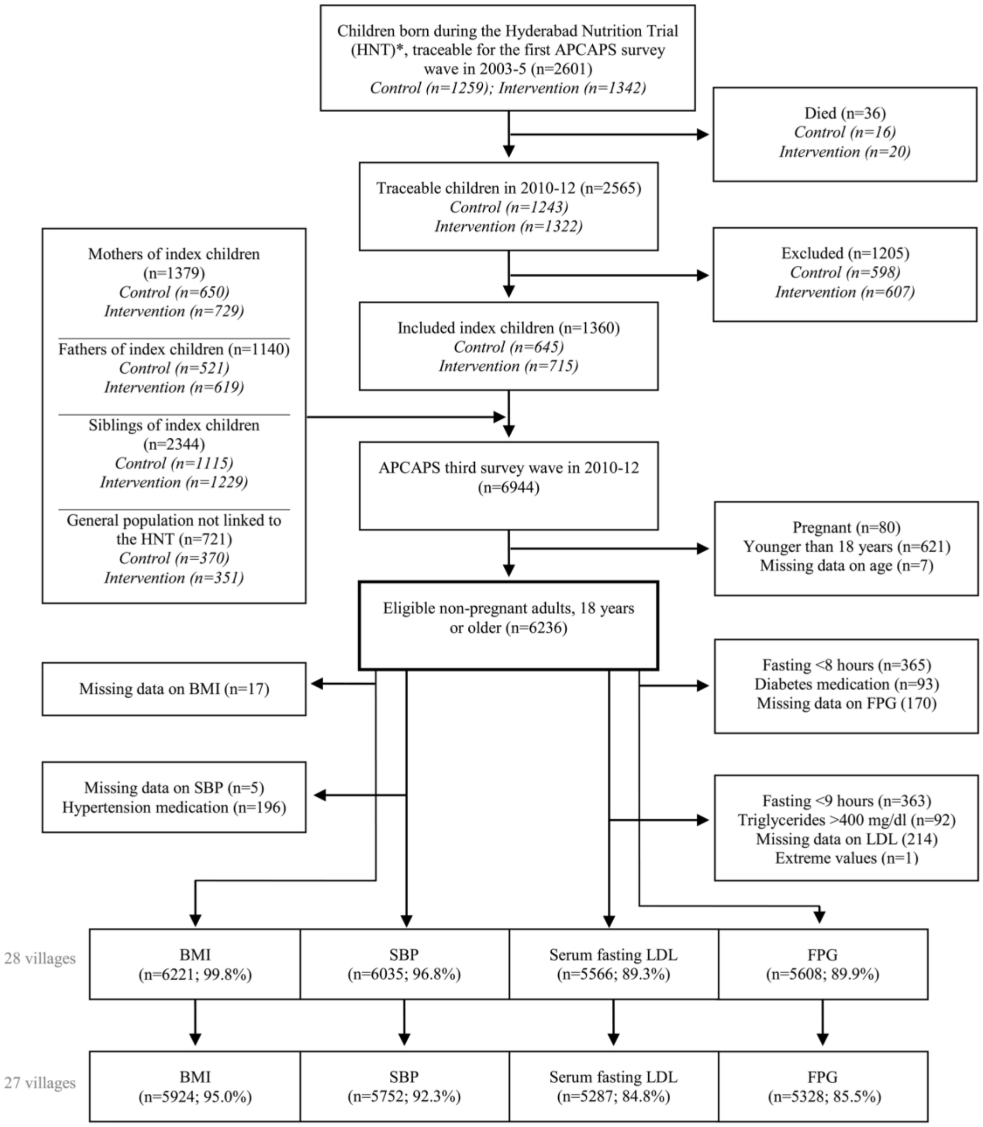

Fig. 1 Participant flow of the Andhra Pradesh Children and Parents Study
BMI – body mass index; FPG – fasting plasma glucose; HNT – Hyderabad Nutrition Trial; LDL – low-density lipoprotein; SBP – systolic blood pressure
*The Hyderabad Nutrition Trial (1987-1990) included 15 intervention and 14 control villages from the Integrated Child Development Services Scheme (a stepped-wedge cluster randomised nutrition supplementation trial)

170x193mm (300 x 300 DPI)

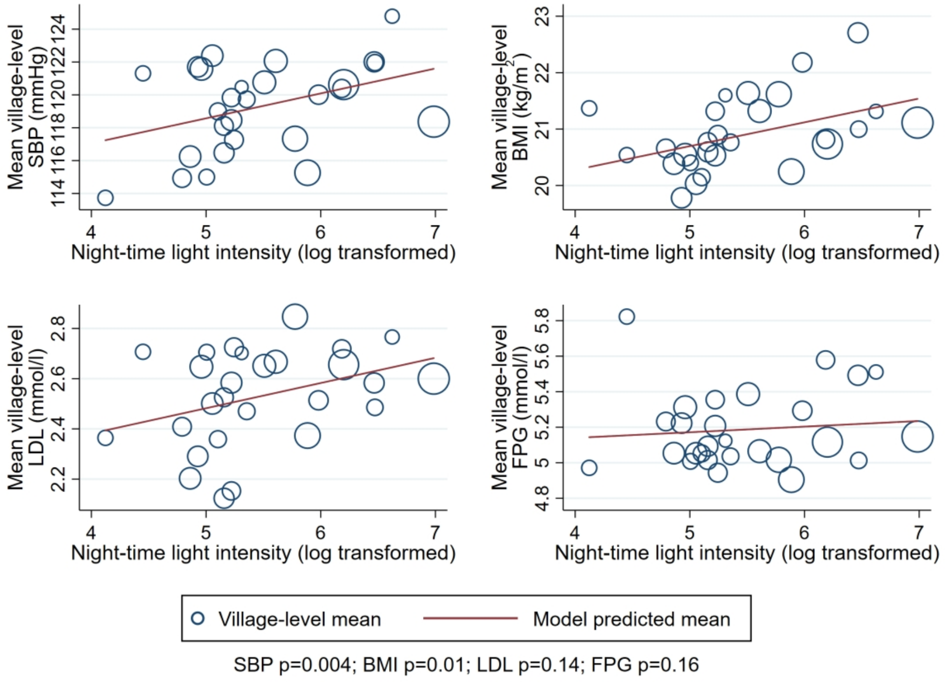

Fig. 2 Crude associations of night-time light intensity with CVD risk factors among APCAPS adults, 2010-12 (n=5937)
APCAPS – Andhra Pradesh Children and Parents Study; BMI – body mass index; CVD – Cardiovascular disease; FPG – fasting plasma glucose; LDL – low-density lipoprotein; NTLI – night-time light intensity; SBP – systolic blood pressure
Model predicted means were derived from multilevel linear regression with clustering by household and village, using individual-level outcome data
Marker size proportional to village size

881x641mm (72 x 72 DPI)

SUPPLEMENTARY FILES

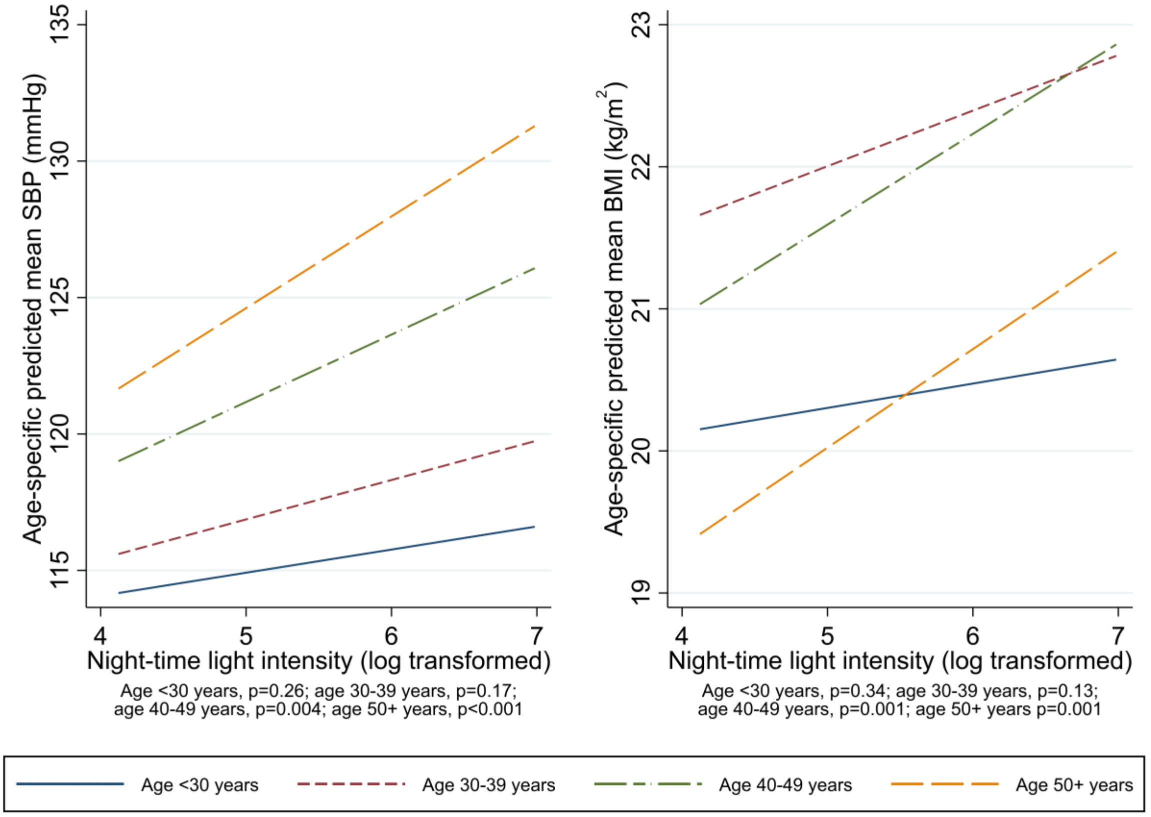

Supplementary Fig. 1 Age-specific crude associations of NTLI with mean BMI and SBP, APCAPS, 2010-12 (n=5937)

APCAPS – Andhra Pradesh Children and Parents Study; BMI – body mass index; NTLI – night-time light intensity; SBP – systolic blood pressure

The fitted lines (model predicted means) were derived from crude multilevel linear regression models with clustering by household and village, using individual-level outcome data

Participants were excluded from analysis of SBP if medicated for hypertension

For peer review only - http://bmjopen.bmj.com/site/about/guidelines.xhtml

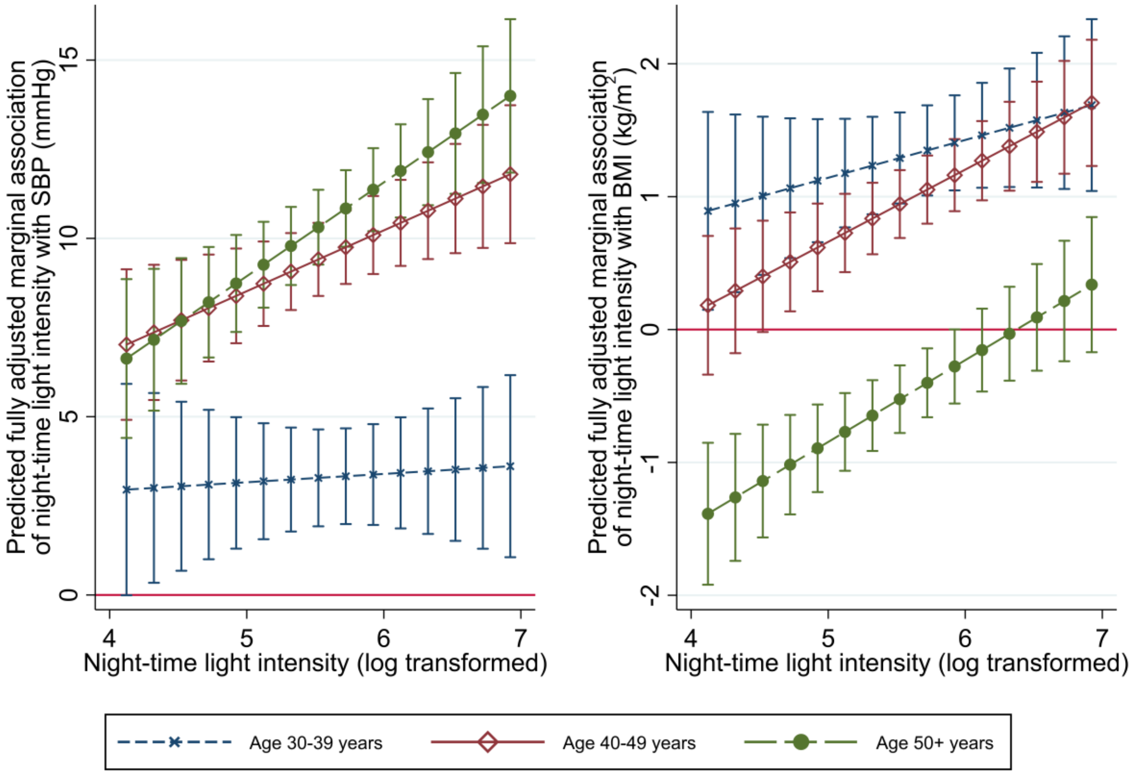

Supplementary Fig. 2 Fully adjusted marginal associations of NTLI with BMI and SBP by age groups (n=5937)

BMI – body mass index; CI – confidence interval; NTLI – night-time light intensity; SBP – systolic blood pressure

Marginal associations, including 95% CIs, were obtained from contrasting age-specific results from multilevel linear regression models with clustering by household and village, and adjusted for gender, caste, religion, marital status and survey season, using individual-level outcome data

Participants were excluded from analysis of SBP if medicated for hypertension

For peer review only - http://bmjopen.bmj.com/site/about/guidelines.xhtml

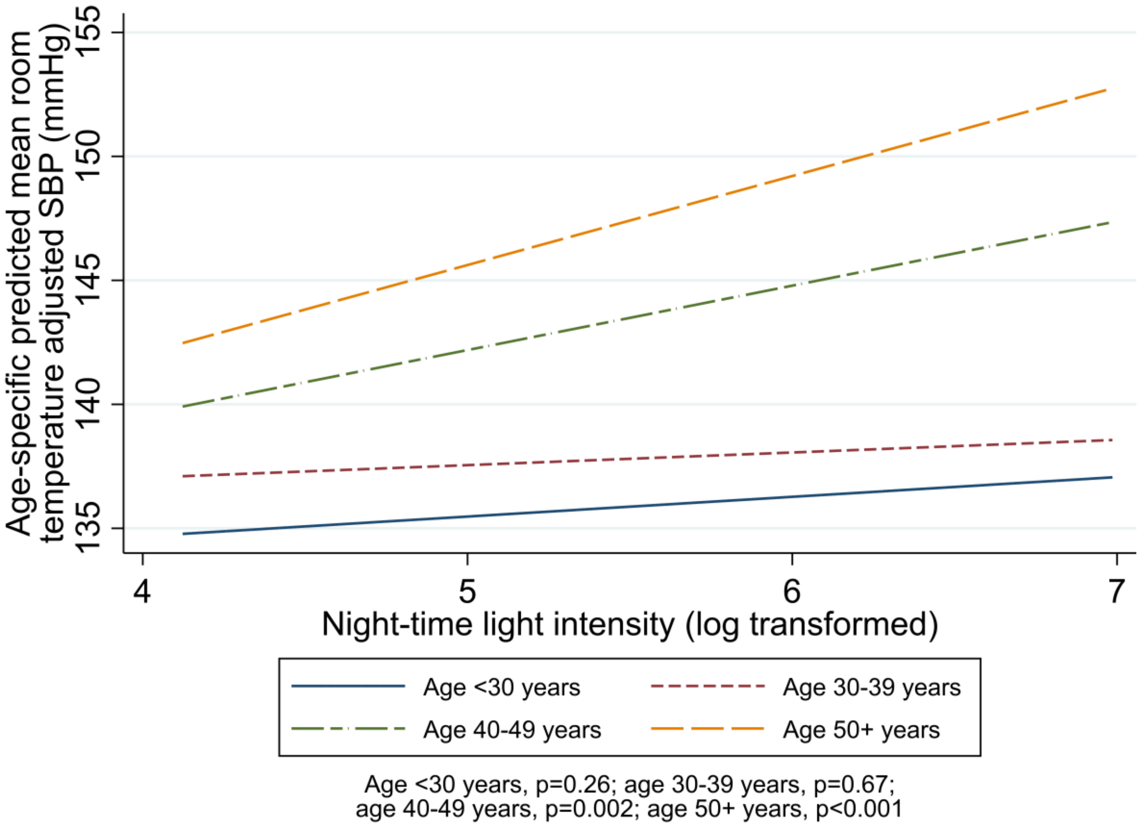

Supplementary Fig. 3 Age-specific room temperature adjusted association of NTLI and SBP among APCAPS adults, 2010-12 (n=3160)

APCAPS – Andhra Pradesh Children and Parents Study; NTLI – night-time light intensity; SBP – systolic blood pressure

The fitted lines (model predicted means) were derived from a crude multilevel linear regression models with clustering by household and village, using individual-level outcome data

Participants were excluded if medicated for hypertensions

For peer review only - http://bmjopen.bmj.com/site/about/guidelines.xhtml

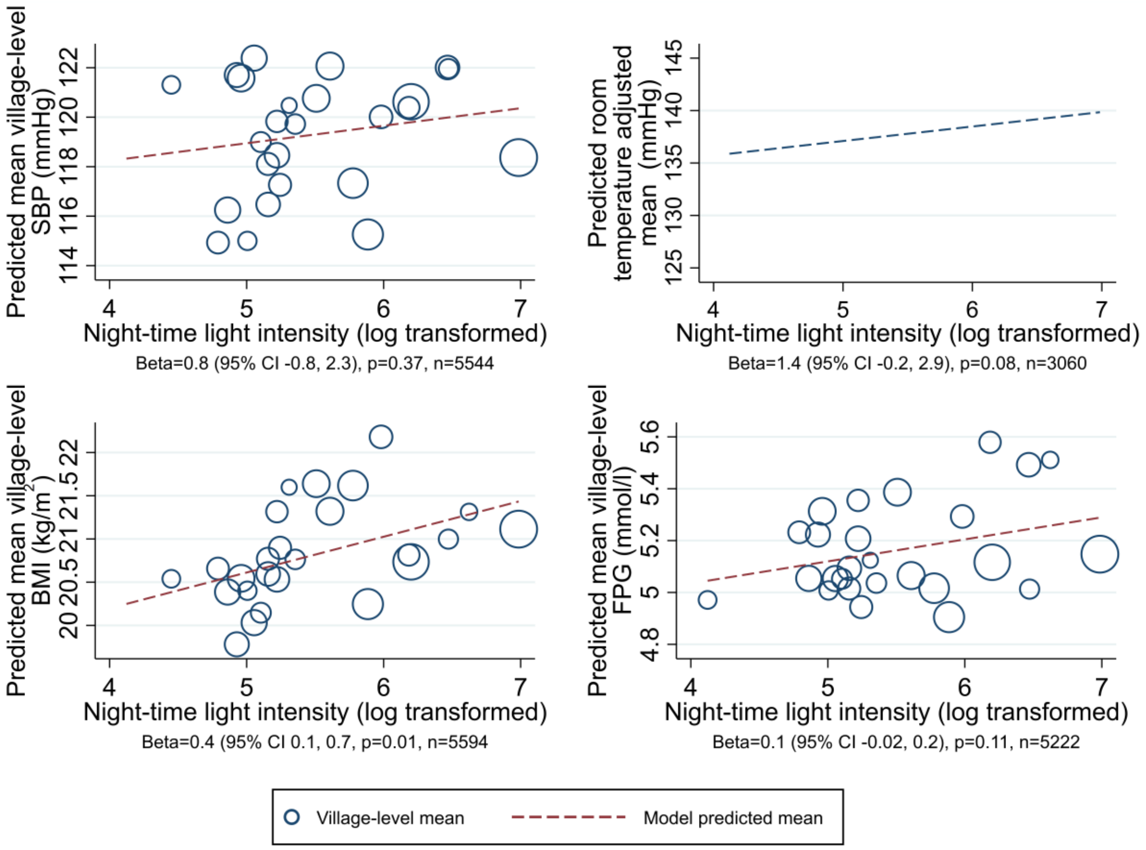

Supplementary Fig. 4 Sensitivity analysis: Crude association of NTLI with SBP, BMI and FPG among APCAPS adults, 2010-12

APCAPS – Andhra Pradesh Children and Parents Study; BMI – body mass index; FPG – fasting plasma glucose; NTLI – night-time light intensity; SBP – systolic blood pressure

Model predicted means were derived from crude and (for SBP) room temperature adjusted multilevel linear regression models with clustering by household and village, using individual-level outcome data

Marker size proportional to village size

Participants from borderline outlier villages were excluded

For peer review only

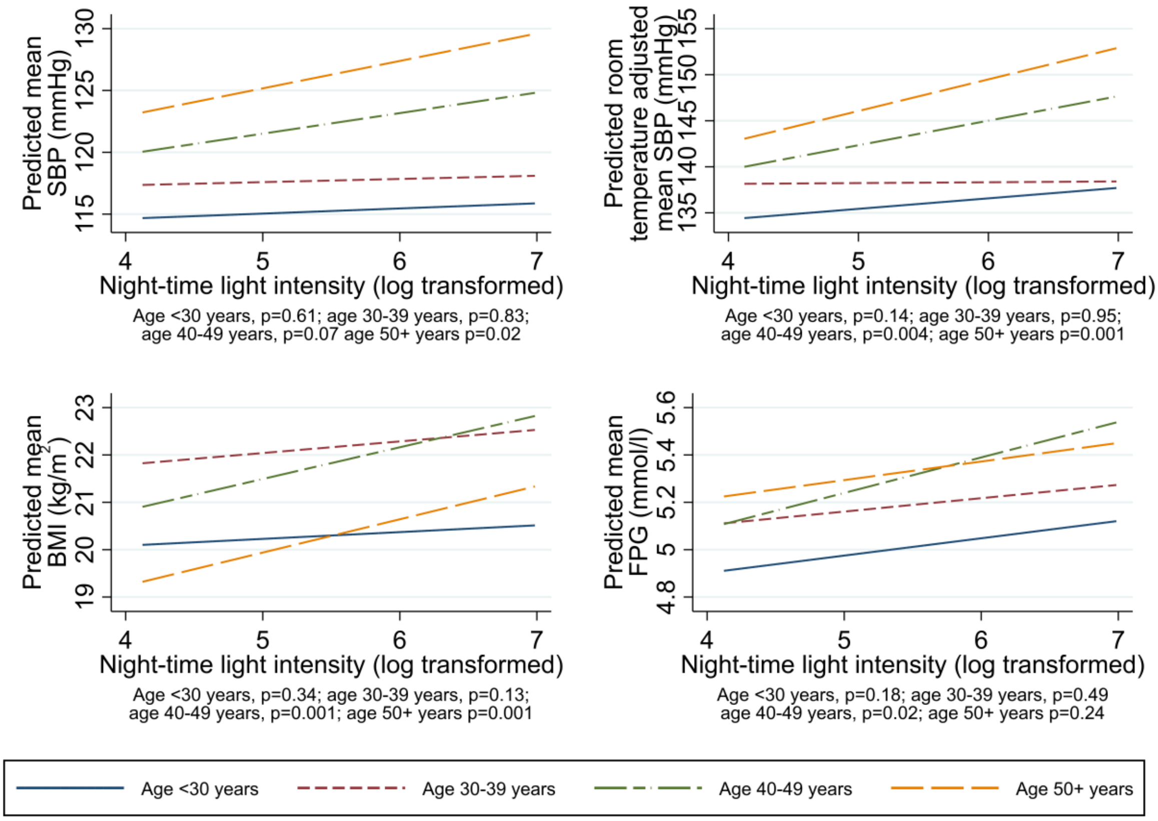

Supplementary Fig. 5 Sensitivity analysis: age-specific crude associations of NTLI with SBP, BMI and FPG

BMI – body mass index; FPG – fasting glucose; NTLI – night-time light intensity; SBP – systolic blood pressure

The fitted lines (model predicted means) were derived from a crude multilevel linear regression models with clustering by household and village, using individual-level outcome data

Participants from borderline outlier villages were excluded

Participants were excluded from analysis of SBP if medicated for hypertension and from analysis of FPG if medicated for diabetes

For peer review only - http://bmjopen.bmj.com/site/about/guidelines.xhtml

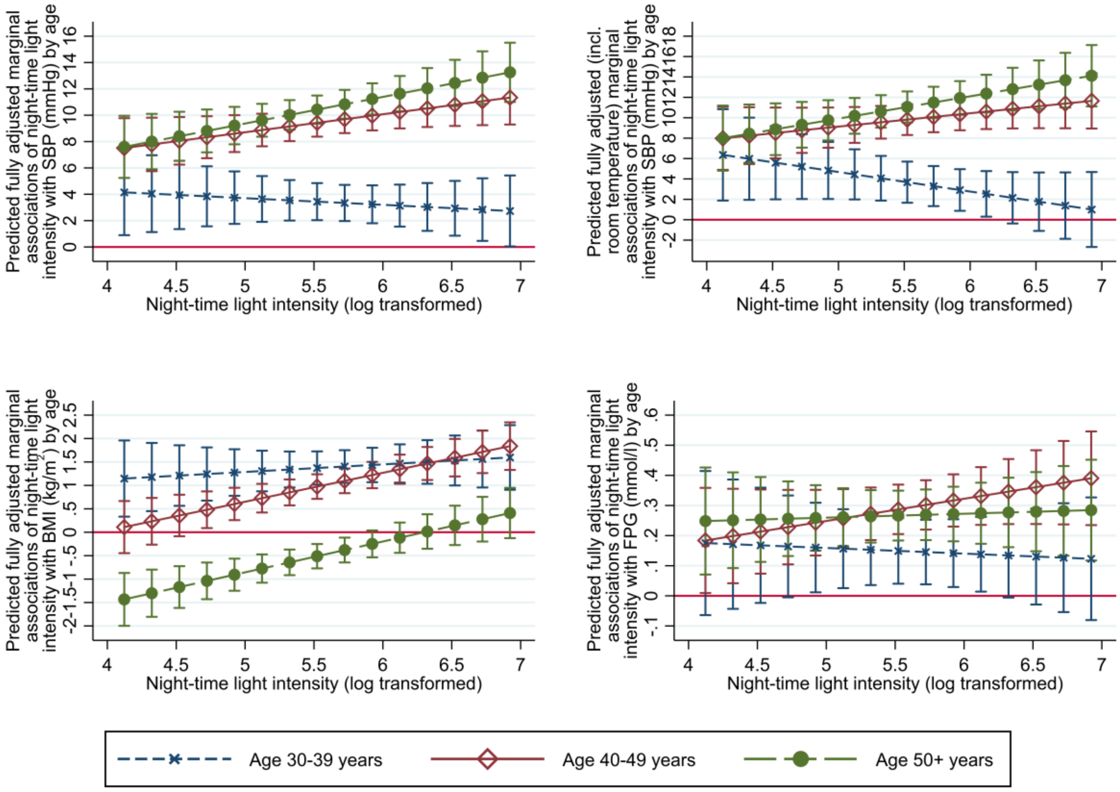

Supplementary Fig. 6 Sensitivity analysis: fully adjusted marginal associations of NTLI with SBP, BMI and FPG by age groups

BMI – body mass index; CI – Confidence Interval; FPG – fasting glucose; SBP – systolic blood pressure

Marginal associations (95% CIs) were obtained from contrasting age-specific results from multilevel linear regression models with clustering by household and village, and adjusted for gender, caste, religion, marital status and survey season, using individual level outcome data

Participants from borderline outlier villages were excluded

Participants were excluded from analysis of SBP if medicated for hypertension and from analysis of glucose if medicated for diabetes

For peer review only - http://bmjopen.bmj.com/site/about/guidelines.xhtml

Supplementary Tab. 1 Age-specific crude and adjusted associations of NTLI with mean BMI and SBP, APCAPS, 2010-12 (n=5937)

CVD risk factors	Age group	n (%)	Age-specific crude model predicted mean (95% CI) at the lowest NTLI (61.7 (4.1 on the log scale))	Age-specific crude model predicted mean (95% CI) at the highest NTLI (1081.1 (7.0 on the log scale))	p-value	Age-specific fully adjusted model predicted mean change with increasing NTLI[b] (adjusted for gender caste, religion, marital status and survey season)	
						β (95% CI)	p-value
**SBP (mmHg)[a]**	<30 years	2806 (48.8)	114.2 (111.9, 116.4)	116.6 (114.2, 119.0)	0.26	0.5 (-0.7, 1.6)	0.45
	30-39 years	544 (9.5)	115.6 (112.2, 119)	119.7 (116.5, 123.0)	0.17	0.7 (-1.2, 2.5)	0.47
	40-49 years	1306 (22.7)	119.0 (116.4, 121.6)	126.1 (123.4, 128.8)	0.004	2.2 (0.8, 3.6)	0.003
	≥50 years	1096 (19.1)	121.7 (118.9, 124.4)	131.3 (128.4, 134.2)	<0.001	3.1 (1.6, 4.6)	<0.001
**BMI (kg/m^2)**	<30 years	2804 (47.3)	20.2 (19.6, 20.7)	20.6 (20.1, 21.2)	0.34	0.04 (-0.3, 0.4)	0.84
	30-39 years	545 (9.2)	21.7 (20.8, 22.5)	22.8 (22.0, 23.6)	0.13	0.3 (-0.2, 0.8)	0.22
	40-49 years	1358 (22.9)	21.0 (20.4, 21.7)	22.9 (22.2, 23.5)	0.001	0.6 (0.2, 1.0)	0.01
	≥50 years	1217 (20.5)	19.4 (18.8, 20.0)	21.4 (20.7, 22.1)	0.001	0.7 (0.2, 1.1)	0.002

APCAPS – Andhra Pradesh Children and Parents Study; BMI – body mass index; NTLI – night-time light intensity; SBP – systolic blood pressure; β – beta coefficient

Model predicted means (95% CIs), β-coefficients (95% CIs) and p-values were obtained from multilevel linear regression models with clustering by household and village, using individual-level outcome data

[a]Participants were excluded if medicated for hypertension

[b]Mean change per whole number increase in log transformed NTLI

For peer review only

For peer review only - http://bmjopen.bmj.com/site/about/guidelines.xhtml

Supplementary Tab. 2 Room temperature adjusted association of NTLI and SBP among APCAPS adults, 2010-12 (n=3160)

					Model predicted age, gender and room temperature adjusted mean change (95% CI) with increasing NTLI[b]			Model predicted fully adjusted mean change with increasing NTLI[b] (adjusted for gender caste, religion, marital status, survey season and room temperature)		
	**n**	**Model predicted room temperature adjusted mean (95% CI) at the lowest NTLI (61.7 (4.1 on the log scale))**	**Model predicted room temperature adjusted mean (95% CI) at the highest NTLI (1081.1 (7.0 on the log scale))**	**p-value**	**n**	**β (95% CI)**	**p-value**	**n**	**β (95% CI)**	**p-value**
**SBP (mmHg)[a]**	3160	136.4 (129.0, 143.8)	140.7 (133.5, 148.0)	<0.001	3159	1.9 (0.7, 3.0)	0.002	3122	1.8 (0.6, 3.0)	0.002

APCAPS – Andhra Pradesh Children and Parents Study; CI – confidence interval; n – sample size; NTLI – night-time light intensity; SBP – systolic blood pressure; β – beta-coefficient

Model predicted means (95% CIs), β-coefficients (95% CIs) and p-values were obtained from multilevel linear regression with clustering by household and village, using individual-level outcome data

[a]Participants were excluded if medicated for hypertension

[b]Mean change per whole number increase in log transformed NTLI

For peer review only

For peer review only - http://bmjopen.bmj.com/site/about/guidelines.xhtml

Additional Tab. 3 Age-specific room temperature adjusted association of NTLI and SBP among APCAPS adults, 2010-12 (n=3160)

SBP (mmHg)[a]	n (%)	Model predicted room temperature adjusted mean (95% CI) at the lowest NTLI (61.7 (4.1 on the log scale))	Model predicted room temperature adjusted mean (95% CI) at the highest NTLI (1081.1 (7.0 on the log scale))	p-value	Model predicted fully adjusted mean change with increasing NTLI[b] (adjusted for gender caste, religion, marital status, survey season and room temperature at clinical assessment)	
					β (95% CI)	p-value
<30 years	1347 (42.6)	134.8 (128.1, 141.4)	137.1 (130.4, 143.7)	0.26	0.8 (-0.5, 2.1)	0.24
30-39 years	305 (9.7)	137.1 (129.6, 144.6)	138.6 (131.3, 145.8)	0.67	-0.2 (-2.5, 2.0)	0.83
40-49 years	838 (26.5)	139.9 (133.0, 146.8)	147.3 (140.6, 154.1)	0.002	2.4 (0.8, 4.0)	0.003
≥50 years	670 (21.2)	142.5 (135.6, 149.4)	152.7 (145.8, 159.7)	<0.001	3.5 (1.8, 5.3)	<0.001

APCAPS – Andhra Pradesh Children and Parents Study; CI – confidence interval; n – sample size; NTLI – night-time light intensity; SBP – systolic blood pressure; β – beta coefficient

Model predicted means (95% CIs), β-coefficients (95% CIs) and p-values were obtained from multilevel linear regression with clustering by household and village, using individual-level outcome data

p<0.001 for effect modification by age group

[a]Participants were excluded if medicated for hypertension

[b]Mean change per whole number increase in log transformed NTLI

For peer review only

For peer review only - http://bmjopen.bmj.com/site/about/guidelines.xhtml

72 **Supplementary Tab. 5 Sensitivity analysis: age-specific crude and adjusted associations of NTLI with SBP, BMI and FPG**

CVD risk factors		n	Age-specific model predicted mean (95% CI) at lowest NTLI	Age-specific model predicted mean (95% CI) at highest NTLI	p-value	Age-specific fully adjusted model predicted mean change with increasing NTLI[c] (adjusted for gender caste, religion, marital status and survey season) n	β (95% CI)	p-value
**SBP (mmHg)[a]**	<30 years	2706	114.8 (112.8, 116.8)	115.9 (113.3, 118.4)	0.61	2615	0.2 (-1.1, 1.5)	0.79
	30-39 years	520	117.4 (114.4, 120.5)	118.1 (114.6, 121.5)	0.83	494	-0.3 (-2.4, 1.7)	0.76
	40-49 years	1252	120.6 (118.3, 122.9)	124.8 (121.9, 127.7)	0.07	1196	1.5 (0.02, 3.1)	0.05
	≥50 years	1066	123.9 (121.6, 126.3)	129.6 (126.5, 132.6)	0.02	1001	2.2 (0.5, 3.8)	0.01
**SBP (mmHg)[a] adjusted for room temperature at clinical assessment**	<30 years	1308	134.8 (127.9, 141.7)	137.7 (130.3, 145.1)	0.14	1294	1.0 (-0.5, 2.5)	0.20
	30-39 years	292	138.2 (130.6, 145.7)	138.4 (130.4, 146.4)	0.95	285	-0.8 (-3.4, 1.8)	0.54
	40-49 years	809	140.9 (133.8, 148.0)	147.6 (140.1, 155.2)	0.004	799	2.3 (0.6, 4.1)	0.01
	≥50 years	651	144.2 (137.1, 151.2)	152.9 (145.2, 160.6)	0.001	644	3.2 (1.3, 5.1)	0.001
**BMI (kg/m2)**	<30 years	2653	20.1 (19.7, 20.6)	20.5 (20.0, 21.1)	0.42	2563	0.02 (-0.3, 0.4)	0.92
	30-39 years	508	21.9 (21.2, 22.6)	22.5 (21.7, 23.3)	0.37	482	0.2 (-0.3, 0.7)	0.51
	40-49 years	1272	21.1 (20.6, 21.6)	22.8 (22.2, 23.5)	0.001	1213	0.6 (0.2, 1.0)	0.002
	≥50 years	1161	19.6 (19.0, 20.1)	21.3 (20.7, 22.0)	<0.001	1094	0.7 (0.3, 1.1)	0.001
**Fasting plasma glucose[b]**	<30 years	2537	4.9 (4.7, 5.1)	5.1 (4.9, 5.3)	0.20	2450	0.03 (-0.1, 0.2)	0.59
	30-39 years	491	5.1 (4.9, 5.4)	5.3 (5.0, 5.5)	0.49	465	0.01 (-0.2, 0.2)	0.88
	40-49 years	1170	5.1 (4.9, 5.3)	5.5 (5.3, 5.7)	0.02	1114	0.1 (-0.03, 0.2)	0.13
	≥50 years	1024	5.2 (5.0, 5.4)	5.4 (5.2, 5.7)	0.25	959	0.05 (-0.1, 0.2)	0.52

For peer review only

BMI – body mass index; CI - confidence interval; n – sample size; NTLI – night-time light intensity; SBP – systolic blood pressure; β – beta coefficient

Model predicted means (95% CIs), β-coefficients (95% CIs) and p-values were obtained from multilevel linear regression models with clustering by household and village, using individual-level outcome data

Participants from borderline outlier villages were excluded

Participants were excluded if medicated for hypertension[a] or diabetes[b]

[c]Mean change per whole number increase in log transformed NTLI

For peer review only - http://bmjopen.bmj.com/site/about/guidelines.xhtml

STROBE 2007 (v4) checklist of items to be included in reports of observational studies in epidemiology*
Checklist for cohort, case-control, and cross-sectional studies (combined)

Section/Topic	Item #	Recommendation	Reported on page #
**Title and abstract**	1	(*a*) Indicate the study's design with a commonly used term in the title or the abstract	2
		(*b*) Provide in the abstract an informative and balanced summary of what was done and what was found	2
**Introduction**			
Background/rationale	2	Explain the scientific background and rationale for the investigation being reported	4-5
Objectives	3	State specific objectives, including any pre-specified hypotheses	5
**Methods**			
Study design	4	Present key elements of study design early in the paper	5+
Setting	5	Describe the setting, locations, and relevant dates, including periods of recruitment, exposure, follow-up, and data collection	
Participants	6	(*a*) *Cohort study*—Give the eligibility criteria, and the sources and methods of selection of participants. Describe methods of follow-up<br>*Case-control study*—Give the eligibility criteria, and the sources and methods of case ascertainment and control selection. Give the rationale for the choice of cases and controls<br>*Cross-sectional study*—Give the eligibility criteria, and the sources and methods of selection of participants	5+
		(*b*) *Cohort study*—For matched studies, give matching criteria and number of exposed and unexposed<br>*Case-control study*—For matched studies, give matching criteria and the number of controls per case	n/a
Variables	7	Clearly define all outcomes, exposures, predictors, potential confounders, and effect modifiers. Give diagnostic criteria, if applicable	6-7
Data sources/ measurement	8*	For each variable of interest, give sources of data and details of methods of assessment (measurement). Describe comparability of assessment methods if there is more than one group	*6-7*
Bias	9	Describe any efforts to address potential sources of bias	8, 14-15
Study size	10	Explain how the study size was arrived at	9, flow diagram
Quantitative variables	11	Explain how quantitative variables were handled in the analyses. If applicable, describe which groupings were chosen and why	8
Statistical methods	12	(*a*) Describe all statistical methods, including those used to control for confounding	8
		(*b*) Describe any methods used to examine subgroups and interactions	8
		(*c*) Explain how missing data were addressed	9, Flow diagram
		(*d*) *Cohort study*—If applicable, explain how loss to follow-up was addressed<br>*Case-control study*—If applicable, explain how matching of cases and controls was addressed	8

For peer review only

		Cross-sectional study—If applicable, describe analytical methods taking account of sampling strategy	
		(*e*) Describe any sensitivity analyses	8
**Results**			
Participants	13*	(a) Report numbers of individuals at each stage of study—eg numbers potentially eligible, examined for eligibility, confirmed eligible, included in the study, completing follow-up, and analysed	9, flow chart
		(b) Give reasons for non-participation at each stage	Flow diagram
		(c) Consider use of a flow diagram	Flow diagram
Descriptive data	14*	(a) Give characteristics of study participants (eg demographic, clinical, social) and information on exposures and potential confounders	9-10
		(b) Indicate number of participants with missing data for each variable of interest	Flow diagram
		(c) *Cohort study*—Summarise follow-up time (eg, average and total amount)	
Outcome data	15*	*Cohort study*—Report numbers of outcome events or summary measures over time	
		Case-control study—Report numbers in each exposure category, or summary measures of exposure	
		Cross-sectional study—Report numbers of outcome events or summary measures	11
Main results	16	(*a*) Give unadjusted estimates and, if applicable, confounder-adjusted estimates and their precision (eg, 95% confidence interval). Make clear which confounders were adjusted for and why they were included	11
		(*b*) Report category boundaries when continuous variables were categorized	8
		(*c*) If relevant, consider translating estimates of relative risk into absolute risk for a meaningful time period	10-12
Other analyses	17	Report other analyses done—eg analyses of subgroups and interactions, and sensitivity analyses	12
**Discussion**			
Key results	18	Summarise key results with reference to study objectives	12-13
Limitations	19	Discuss limitations of the study, taking into account sources of potential bias or imprecision. Discuss both direction and magnitude of any potential bias	14-15
Interpretation	20	Give a cautious overall interpretation of results considering objectives, limitations, multiplicity of analyses, results from similar studies, and other relevant evidence	3, 14
Generalisability	21	Discuss the generalisability (external validity) of the study results	14
**Other information**			
Funding	22	Give the source of funding and the role of the funders for the present study and, if applicable, for the original study on which the present article is based	17

*Give information separately for cases and controls in case-control studies and, if applicable, for exposed and unexposed groups in cohort and cross-sectional studies.

Note: An Explanation and Elaboration article discusses each checklist item and gives methodological background and published examples of transparent reporting. The STROBE checklist is best used in conjunction with this article (freely available on the Web sites of PLoS Medicine at http://www.plosmedicine.org/, Annals of Internal Medicine at http://www.annals.org/, and Epidemiology at http://www.epidem.com/). Information on the STROBE Initiative is available at www.strobe-statement.org.

For peer review only

BMJ Open

IS NIGHT-TIME LIGHT INTENSITY ASSOCIATED WITH CARDIOVASCULAR DISEASE RISK FACTORS AMONG ADULTS IN EARLY STAGE URBANISATION IN SOUTH INDIA? A CROSS SECTIONAL STUDY OF THE ANDHRA PRADESH CHILDREN AND PARENTS STUDY

Journal:	*BMJ Open*
Manuscript ID	bmjopen-2019-036213.R1
Article Type:	Original research
Date Submitted by the Author:	16-Jul-2020
Complete List of Authors:	Sorensen, Tina Bonde; London School of Hygiene and Tropical Medicine, Department of Population Health<br>Wilson, Robin; University of Southampton, Geography & Environment<br>Gregson, John; London School of Hygiene and Tropical Medicine, Medical Statistics<br>Shankar, Bhavani; The University of Sheffield, Department of Geography<br>Dangour, Alan; London School of Hygiene and Tropical Medicine, Population Health<br>Kinra, Sanjay; London School of Hygiene and Tropical Medicine, Non-communicable Disease Epidemiology
**Primary Subject Heading**:	Cardiovascular medicine
Secondary Subject Heading:	Global health, Research methods, Public health, Nutrition and metabolism, Epidemiology
Keywords:	EPIDEMIOLOGY, Cardiac Epidemiology < CARDIOLOGY, Hypertension < CARDIOLOGY

SCHOLARONE™
Manuscripts

I, the Submitting Author has the right to grant and does grant on behalf of all authors of the Work (as defined in the below author licence), an exclusive licence and/or a non-exclusive licence for contributions from authors who are: i) UK Crown employees; ii) where BMJ has agreed a CC-BY licence shall apply, and/or iii) in accordance with the terms applicable for US Federal Government officers or employees acting as part of their official duties; on a worldwide, perpetual, irrevocable, royalty-free basis to BMJ Publishing Group Ltd ("BMJ") its licensees and where the relevant Journal is co-owned by BMJ to the co-owners of the Journal, to publish the Work in this journal and any other BMJ products and to exploit all rights, as set out in our licence.

The Submitting Author accepts and understands that any supply made under these terms is made by BMJ to the Submitting Author unless you are acting as an employee on behalf of your employer or a postgraduate student of an affiliated institution which is paying any applicable article publishing charge ("APC") for Open Access articles. Where the Submitting Author wishes to make the Work available on an Open Access basis (and intends to pay the relevant APC), the terms of reuse of such Open Access shall be governed by a Creative Commons licence – details of these licences and which Creative Commons licence will apply to this Work are set out in our licence referred to above.

Other than as permitted in any relevant BMJ Author's Self Archiving Policies, I confirm this Work has not been accepted for publication elsewhere, is not being considered for publication elsewhere and does not duplicate material already published. I confirm all authors consent to publication of this Work and authorise the granting of this licence.

For peer review only

IS NIGHT-TIME LIGHT INTENSITY ASSOCIATED WITH CARDIOVASCULAR DISEASE RISK FACTORS AMONG ADULTS IN EARLY STAGE URBANISATION IN SOUTH INDIA? A CROSS SECTIONAL STUDY OF THE ANDHRA PRADESH CHILDREN AND PARENTS STUDY

Tina B Sørensen,[1*] Robin Wilson,[2] John Gregson,[1] Bhavani Shankar,[3,4] Alan D Dangour[1,4] and Sanjay Kinra[1]

*Corresponding author. Faculty of Epidemiology and Population Health, London School of Hygiene and Tropical Medicine (LSHTM), London WC1E 7HT, United Kingdom. E-mail: tinabondesoerensen@gmail.com, Telephone +44 (0) 2072994723. Orcid: https://orcid.org/0000-0003-1517-6159

[1]Faculty of Epidemiology and Population Health, London School of Hygiene and Tropical Medicine, Keppel Street, London WC1E 7HT, UK

[2]Geography & Environment, University of Southampton, University Road, Southampton SO17 1BJ, UK

[3]Department of Geography, The University of Sheffield, Winter Street, Sheffield S3 7ND

[4]London Centre for Integrative Research in Agriculture and Health (LCIRAH), 36 Gordon Square, London WC1H 0PD, UK

E-mails: tinabondesoerensen@gmail.com, robin@rtwilson.com, john.gregson@lshtm.ac.uk, b.shankar@sheffield.ac.uk, alan.dangour@lshtm.ac.uk, sanjay.kinra@lshtm.ac.uk

Word count 283 words (abstract), 4938 words (text)

For peer review only

For peer review only - http://bmjopen.bmj.com/site/about/guidelines.xhtml

ABSTRACT

Objectives: To explore associations of night-time light intensity (NTLI), a novel proxy for continuous urbanisation levels, with mean systolic blood pressure (SBP), body mass index (BMI), fasting serum low-density lipoprotein (LDL), and fasting plasma glucose (FPG), among adults in early stage urbanisation in Telangana, South India.

Design: Cross-sectional analysis of the third wave of the Andhra Pradesh Children and Parents Study cohort.

Setting: 28 villages representing a continuum of urbanisation levels, ranging from rural settlement to medium sized town in Telangana, South India.

Participants: Data were available from 6944 participants, 6236 of whom were eligible after excluding pregnant women, participants younger than 18 years of age and participants missing data for age. Participants were excluded if they did not provide fasting blood samples, had implausible or missing outcome values, were medicated for hypertension or diabetes, or had triglyceride levels invalidating derived LDL. The analysis included 5924 participants for BMI, 5752 participants for SBP, 5287 participants for LDL and 5328 participants for FPG.

Results: Increasing NTLI was positively associated with mean BMI, SBP and LDL but not FPG. Adjusted mean differences across the range of village-level NTLI were 1.0 kg/m^2 (95% CI 0.01, 1.9) for BMI; 4.2 mmHg (95% CI 1.0, 7.4) for SBP; 0.3 mmol/l (95% CI -0.01, 0.7) for LDL; and -0.01 mmol/l (95% CI -0.4, 0.4) for FPG. Associations of NTLI with BMI and SBP were stronger in older age groups.

Conclusion: The association of NTLI with CVD risk factors identify NTLI as a potentially important tool for exploring urbanisation-related health. Consistent associations of moderate increases in urbanisation levels with important CVD risk factors warrant prevention strategies to curb expected large public health impacts from continued, rapid urbanisation in India.

Key words: Urbanisation, night-time light intensity (NTLI), non-communicable disease (NCD), cardiovascular disease (CVD), CVD risk factor, Andhra Pradesh Children and Parents Study (APCAPS)

For peer review only - http://bmjopen.bmj.com/site/about/guidelines.xhtml

STRENGTHS AND LIMITATIONS OF THIS STUDY

- We used a novel, standardised proxy of continuous urbanisation levels, remote sensing data on night-time light intensity, measured at the village level.
- The villages had experienced rapid uneven urbanisation during the decades preceding the study and represented a continuum of urbanisation levels ranging from a rural settlement to a medium sized town.
- The study used a large population-based sample from 28 urbanising villages at early stages of urbanisation.
- Differences between excluded and included participants warranted some caution of generalising the findings to the general population.
- The cross-sectional design prevented us from making causal inferences or exploring the impact of urbanisation rate on CVD risk factors.

For peer review only

For peer review only - http://bmjopen.bmj.com/site/about/guidelines.xhtml

BACKGROUND

Cardiovascular diseases (CVDs), primarily coronary heart disease, are the leading causes of death (28%) and disability adjusted life years (14%) in India.[1] They occur 5-10 years earlier than in Western populations[2] and the age-standardised mortality rate (272 per 100000 persons) exceeds the global average and rates of some high-income countries.[3 4] Individuals and societies in India therefore continue to experience substantial losses of productivity and income.[1 5-7]

Urbanisation and cardiovascular disease risk factors

Urbanisation is often described as a key driver of CVDs in low- and middle-income (LMICs) acting on CVDs and their risk factors through interrelated changes in social, physical and build environments, socio-demographics, lifestyle and mental health. Some of these change are associated with harmful impacts on cardiovascular health, e.g. due to reduced physical activity and nutrition transitions, while others may be beneficial, e.g. through more and better education and access to health and social services.[8-12] A number of simple rural-urban comparisons and migration studies provide insight to the association of urbanisation level with CVD risk factors, such as overweight and obesity, hypertension, diabetes, and dyslipidaemia.[8 13-21] The broad rural-urban dichotomies are useful, however insufficient for exploring which factors of urbanisation are most important for health in LMICs.[22] In part because no universal definition exists of 'rural' and 'urban' environments, and data on neighbourhood characteristics are often not available from these settings.[22] Furthermore, residents' characteristics tend to be more similar within than between urban and rural areas. For example, urban residents may on average enjoy wider access to goods and services, higher levels of education, occupation, affluence etc., whereas the opposite may be the case in rural areas.[22 23] Such limited discordance at either end of the urbanisation continuum makes it difficult to tease out the underlying mechanisms of observed differences in CVD risk between the two extremes. Urbanisation factors may change at different rates with urbanisation,[22] however, and thus vary widely across the continuum from rural to urban areas. It may therefore be more useful and informative to study the impact of urbanisation level on CVD risk factors in transitioning populations, i.e. populations representing various levels of urbanisation across the continuum, where the underlying mechanisms may be easier to separate. Unfortunately, it is challenging to accurately measure urbanisation as a continuum, whether using single- (e.g. population density) or multi-component classification systems (e.g. housing types and densities, economic activities, physical environment, services, etc.).[22] This is particularly true in LMICs where these types of data are often scarce.

For peer review only - http://bmjopen.bmj.com/site/about/guidelines.xhtml

Night-time light intensity as a measure of urbanisation level

As an alternative to physical data based scales, remote sensing data have been widely used to characterise urban landscapes and scale of urbanisation.[24 25] The night-time light intensity (NTLI) data, obtained by the United States' Defence Meteorological Satellite Programme's Operational Linescan System, are suggested a valid proxy for urbanisation level and dynamics due to their strong and consistent relationships with demographic and economic indicators of urbanisation such as urban build up area, population density, economic activity, and energy consumption at global, regional and local levels.[24-27] The NTLI data, which have near global coverage and a freely available historical data archive dating back to 1992,[28] offer a unique opportunity to study the association of urbanisation, measured by a single standard measure world-wide, with CVD risk.

Study aim to address evidence gaps

This study aimed to explore associations of urbanisation level (measured by a continuum of NTLI) with mean levels of four leading risk factors for CVDs, systolic blood pressure (SBP), body mass index (BMI), fasting serum low-density lipoprotein (LDL), and fasting plasma glucose (FPG), in adults from 28 villages in rapidly urbanising Telangana, South India. Improved understanding of the association of urbanisation level and CVD risk factors in setting transitioning from rural areas to greater levels of urbanisation will help inform policy for disease prevention and control. Such policies are imperative in India, where economic growth and urbanisation continues, to avoid further adverse economic and social impacts of (premature) CVD morbidity and mortality.

MATERIALS AND METHODS

Study design, setting and participants

The current cross-sectional study analysed data from the third and most recent survey wave of the Andhra Pradesh Children and Parents Study (APCAPS)[29] and was set in 28 villages near Hyderabad in Telangana, South India. The APCAPS birth cohort was originally established (in 2003-05) to investigate the short- and long-term effects of early life nutrition on CVDs and their risk factors, as a long-term follow-up of the Hyderabad Nutrition Trial (HNT). The HNT (1987-90) was a cluster-randomised community trial, in which all pregnant women and young children received (15 villages) or awaited (14 villages) a nutrition intervention. The APCAPS villages experienced uneven rates of urbanisation over time, and from the second survey wave the cohort was expanded to include more participants and information on CVD risk factors, to investigate diverse environmental, lifestyle and metabolic risk factors for CVDs in the birth cohort as well as among the children's

For peer review only - http://bmjopen.bmj.com/site/about/guidelines.xhtml

parents and siblings.[30 31] The third and most recent data wave (conducted in 2010-2012, n=6944[1]) was selected for cross-sectional analysis due to its large adult population (≥18 years), wide age range and varying levels of urbanisation. Details on the recruitment of APCAPS participants from the first to the third wave of the APCAPS are presented in Fig. 1. Further details on the cohort are reported elsewere.[30 31]

Fig. 1 Sampling and participant flow for the third survey wave of the APCAPS

APCAPS – Andhra Pradesh Children and Parents Study; BMI – body mass index; FPG – fasting plasma glucose; HNT – Hyderabad Nutrition Trial; LDL – low-density lipoprotein; SBP – systolic blood pressure

* The Hyderabad Nutrition Trial (1987-1990) included 15 intervention and 14 control villages from the Integrated Child Development Services Scheme (stepped-wedge cluster randomised nutrition supplementation trial)

Sample size and selection

All non-pregnant adults (18 years or older) from the third APCAPS survey wave were eligible for inclusion (n=6236/6944) (Fig.1). Participants who reported taking medication for outcome-related conditions were excluded to reduce the risk of biasing results, e.g. towards the null if participants with medically controlled blood pressure sustained high levels of risk factors for hypertension. Participants were excluded from analyses of glucose if they reported fasting for less than eight hours at the time of giving the blood (n=366) or were currently taking medication (tablets or insulin) for diabetes (n=94). Participants were excluded from analysis of LDL if they reported fasting for less than nine hours (n=364) or had triglyceride levels above 400 mg/dl[32] (n=92). Participants who reported taking antihypertensive medication were excluded from analyses of SBP (n=197) (Fig. 1). One of the 28 surveyed villages was significantly larger and had a considerably higher NTLI value than the other villages and its participants were excluded (n=297) to avoid overreliance of results from this outlier village. The final sample sizes by study outcome are presented in Fig. 1.

Data

Data collection and procedures

Trained field staff collected data on health and socio-demographic information in the local language, Telugu, using semi-structured questionnaires. Trained field staff and medical doctors performed the clinical assessments

[1] $n_{(index\ children)}=1360$; $n_{(mothers)}=1379$; $n_{(fathers)}=1140$; $n_{(siblings)}=2344$; $n_{(general\ population)}=721$

For peer review only - http://bmjopen.bmj.com/site/about/guidelines.xhtml

in local clinics and at the National Institute of Nutrition in Hyderabad. Venous fasting (eight to 12 hours) blood samples were collected, separated within 30 minutes, and stored locally at -20°C. Biochemical assays were performed using the Cobas311 auto-analyser at the Genetics and Biochemistry Laboratory at The South Asia Network for Chronic Diseases of the Public Health Foundation of India, New Delhi. To define APCAPS village boundaries, trained field staff performed aerial tracing of Bing satellite imagery, using Open Street Map software and GPS-based ground surveying during 2012-13.

The NTLI data, which is collected by the United States' Defence Meteorological Satellite Programme's Operational Linescan System, was obtained online from the National Oceanic and Atmospheric administration,[33] through the Google Earth Engine.[34] The raw satellite data is processed into annual averages of the intensity of light emitted from the earth's surface at night from persistent sources associated with human settlement, i.e. excluding transient light sources such as lightning, fire; aurora, solar and lunar light. The processed data are aggregated into 30 arc seconds grids (equivalent to a resolution of approximately 1km^2 pixels)[35] and published as unitless digital numbers ranging from 0 (no light) to 63 (light saturation).[24 27] We obtained the NTLI imagery.

Defining urbanisation level

Urbanisation is commonly described as the process and dynamics of permanent concentration of a population in urban settlements and/or the adaption of lifestyles associated with urban environments, e.g. sedentary lifestyle or nutrition trantision.[10 11 36 37] Although internal migration to urban areas is common in India, most urbanisation is reported to be driven by the natural growth and development of rural areas and their subsequent reclassification into urban settlements.[38] The term 'early stage urbanisation' represents the early transition of rural settlements towards more urbanised physical environments and ways of life in the absence of development into large towns or cities. For the purpose of this study the term 'urbanisation level' defines the level of urbanisation of a village at the time of survey in 2010-2012, as measured by NTLI (described in the following section).

Determining urbanisation levels of APCAPS villages

The data defining village boundaries were overlaid with the NTLI grids from 2011 (technical issues with satellite sensors prevented the use of NTLI data from 2012).[28] Twenty-four villages were intersected by two or more pixels, which in some cases had different NTLI values. Therefore, to obtain one NTLI value (urbanisation level) per village, we used a super-resolution method to resample the NTLI images to a higher resolution, before summing the unitless digital numbers of pixels within or intersecting with the village boundary to produce an

For peer review only - http://bmjopen.bmj.com/site/about/guidelines.xhtml

overall village level. This ensured giving the appropriate weighting to each of the pixel values present over each village. Finally the data were calibrated using the ridgeline sampling regression methods[28] and oversaturation of light was removed manually. Since the first survey wave in 2003-2005, the 28 APCAPS villages had changed from rural agricultural communities with similar NTLI levels to represent a continuum of NTLI levels with growing differentiation at higher levels.

Selection of study outcomes

For this analysis, we pre-specified the four outcomes, BMI, SBP, LDL and FPG, from a number of CVD risk factors available in the APCAPS, due to their well-established and strong associations with CVDs and their modifiable nature.[32 39] The selection of continuous outcomes also enabled exploration of linear relationships between increasing level of NTLI and the CVD risk factors.

Measurement of study outcomes

APCAPS cohort data were collected using standardised approaches. Weight was measured twice to the nearest 0.1 kg with digital weight scales (Seca Leicester 899; Chasmors Ltd, London, United Kingdom (UK)), while wearing light clothing and no shoes. Standing height was measured twice to the nearest 0.1 cm with a portable stadiometer (Seca Leicester height measure; Chasmors Ltd, London, UK). The two weight and height measurements were averaged and BMI calculated for the analyses as weight (kg)/height (m)^2. Blood pressure was measured three times at the right arm in the sitting position using a digital device and appropriate sized cuff (Omron hem 7300; Matsusaka Co., Japan). The last two measurements of SBP were averaged for the analysis. Fasting plasma glucose was assessed on the day of sampling by enzymatic Glucose Oxidase/Peroxidase-4-Aminophenazone-pPenol method (Randox Laboratories; London, UK). Fasting LDL was derived indirectly using the Fridewald-Fredrickson formula: LDL=total cholesterol–high-density lipoprotein–triglycerides/5 (all measures in mg/dl).[32 40]

Biochemical assays of variables needed to derive study outcomes

Serum total cholesterol and triglycerides were assessed by enzymatic methods using Cholesterol Oxidase-Peroxidase-4-Aminophenazone-Phenol and Glycerol Phosphate Oxidase-Peroxidase-4-Aminophenazone-Phenol (Roche Diagnostics GmbH; Mannheim, Germany). High-density lipoprotein (used for derivation of LDL) was derived by direct method using Polyethylene Glycol modified Cholesterol Esterase, Cholesterol Oxidase and Dextran Sulphate (Roche Diagnostics GmbH; Mannheim, Germany).

For peer review only - http://bmjopen.bmj.com/site/about/guidelines.xhtml

Quality assurance

Trained field staff and medical doctors followed standardised procedures and anthropometric equipment were calibrated at the start of every clinic. Reproducibility of clinical measurements were evaluated by repeating measures on a 5% random subsample after one to three weeks. The consistency was high for all measures with intra-class correlation coefficient for anthropometric measurements >0.98; blood pressure >0.85; and biochemical assays >0.94.[41] The quality of biochemical assays were monitored by the Cardiac Biochemistry laboratory at the All India Institute of Medical Sciences, which participates in the UK National External Quality Assessment Programme and the External Quality Assessment Scheme of Randox International.

Patient and Public Involvement

Study participants (patients) and the public were not involved in the design or planning of the study.

Statistical methods

Variables were assessed for outliers, cleaned accordingly, and examined for normality. Night-time light intensity data were non-normally distributed and log-transformed for analysis. To preserve study power, we allowed sample sizes to vary by outcomes. Mixed effects linear regression models with clustering by household and village were fitted in all analyses to account for the hierarchical nature of the data and the sampling strategy. Night-time light intensity (log transformed), BMI, SBP, LDL, FPG, age, and room temperature at clinical assessment (the latter for analysis of SBP) were included as continuous variables. Gender (women, men), caste (General caste, Scheduled caste, Scheduled tribe, Other backward class, other), religion (Muslim, Hindu, Christian, other), current marital status (married, unmarried), season of survey and season of birth (summer (March-May), South West monsoon (June-October), winter (November-February)) were included as categorical variables. Study characteristics were presented by thirds of ranked NTLI, referred to as low, medium, and high levels. We evaluated potential confounders for each outcome using the double-selection method.[42] One final set of covariates, identified as potential confounders for at least one exposure-outcome relationship, were used in analyses of all four outcomes. Level of urbanisation may affect CVD risk profiles of different genders and age groups differently due to e.g. gender inequality, employment patterns, and readiness to adopt new lifestyles and behavious.[23 43-45] We therefore tested for interaction by gender and age. We did not consider socio-demographic factors such as education, occupation, and socio-economic status as potential confounders or effect modifiers due to their importance on the causal pathway from urbanisation level to CVD risk factors. We did not adjust the main analysis of SBP for room temperature at clinical because a large proportion of data was missing for this

For peer review only - http://bmjopen.bmj.com/site/about/guidelines.xhtml

variable ($n_{(missing)}$=2648 [45%]). However, a supplementary analysis for room temperature-adjusted SBP were presented in the Supplementary Material.

A sensitivity analyses were performed to assess the influence of trial status among index children from the HNT. A second sensitivity analysis was performed to evaluate whether borderline outlier villages (with higher or lower observed mean outcomes than the majority of villages) influenced regression estimates considerably. Differences between included and excluded participants were examined with Chi square, and linear, and logistic mixed effects models with clustering by village and household. All analyses were performed in Stata 14 (StataCorp, College Station, TX, USA).

For peer review only

RESULTS

Study characteristics

A total of 6236 non-pregnant adults were eligible for inclusion in the current study (90% of the third APCAPS survey wave). After removing extremes and participants with missing outcome data for specific analyses, the final samples came to 5924 participants for BMI; 5752 participants for SBP; 5287 participants for LDL; and 5328 participants for FPG (Fig. 1). Approximately half of participants were women (Tab. 1). The overall median age was 32 years (interquartile range (IQR) 24, 48), with women on average older than men. Men and women most frequently identified with other backward caste and Hindu religion. Women were more likely to be married (76%) than men (59%). Village-level NTLI ranged from 61.7 (equivalent to 4.1 on the log scale) to 1081.1 (equivalent to 7.0 on the log scale). The distribution of socio-demographic characteristics were similar across low, medium and high level of NTLI. None of the included (non-medicated) participants reported ever being diagnosed with heart disease, including stroke.

Tab. 1 Participant characteristics of APCAPS adults from 27 villages, 2010-12 (n=5932)

	NTLI[a], women (n=2753 (46 %))			NTLI[a], men (n=3179 (54 %))		
	Low	Medium	High	Low	Medium	High
**Night-time light intensity (NTLI), median (IQR)**	138.2 (121, 149)	202.4 (185, 273)	492.8 (485, 753)	138.2 (129, 149)	202.4 (185, 273)	492.8 (396, 753)
**Age, median (IQR)**	40 (25, 46)	40 (25, 46)	38 (25, 45)	28 (23, 51)	29 (24, 50)	28 (23, 49)
**Age group, n (%)**						
<30 years	299 (38.7)	372 (38.2)	416 (41.3)	489 (54.5)	595 (52.7)	636 (55.2)

10

	NTLI[a], women (n=2753 (46 %))			NTLI[a], men (n=3179 (54 %))		
	Low	Medium	High	Low	Medium	High
30-39 years	85 (11.0)	106 (10.9)	134 (13.3)	57 (6.4)	74 (6.6)	91 (7.9)
40-49 years	261 (33.8)	348 (35.8)	348 (34.5)	98 (10.9)	151 (13.4)	154 (13.4)
50+ years	127 (16.5)	147 (15.1)	110 (10.9)	254 (28.3)	309 (27.4)	271 (23.5)
Total	772 (100)	973 (100)	1008 (100)	898 (100)	1129 (100)	1152 (100)
**Caste, n (%)**						
General caste	51 (6.6)	79 (9.2)	76 (7.5)	51 (5.7)	89 (8.9)	63 (5.5)
Scheduled caste	277 (35.9)	375 (43.6)	292 (29.0)	331 (36.9)	428 (42.6)	335 (29.1)
Scheduled tribe	9 (1.2)	3 (0.4)	5 (0.5)	11 (1.2)	3 (0.3)	7 (0.6)
Other backward	425 (55.1)	374 (43.5)	628 (62.3)	488 (54.3)	455 (45.3)	739 (64.2)
Other	9 (1.2)	29 (3.4)	7 (0.7)	17 (1.9)	30 (3.0)	8 (0.7)
Total	771 (100)	860 (100)	1008 (100)	898 (100)	1005 (100)	1152 (100)
**Religion, n (%)**						
Muslim	19 (2.5)	37 (4.3)	71 (7.0)	16 (1.8)	45 (4.5)	64 (5.6)
Hindu	742 (96.2)	786 (91.4)	919 (91.2)	875 (97.4)	931 (92.6)	1079 (93.7)
Christian	9 (1.2)	37 (4.3)	16 (1.6)	7 (0.8)	29 (2.9)	8 (0.7)
Other	1 (0.1)	0 (0)	2 (0.2)	0 (0)	0 (0)	1 (0.1)
Total	771 (100)	860 (100)	1008 (100)	898 (100)	1005 (100)	1152 (100)
**Marital status, n (%)**						
Not married	196 (25.4)	238 (24.5)	229 (22.7)	381 (42.4)	459 (40.7)	487 (42.3)
Married	576 (74.6)	734 (75.5)	779 (77.3)	517 (57.6)	669 (59.3)	665 (57.7)
Total	772 (100)	972 (100)	1008 (100)	898 (100)	1128 (100)	1152 (100)
**Hyderabad Nutrition Trial, n (%)**						
Control	643 (83.3)	826 (84.9)	835 (82.8)	667 (74.3)	852 (75.5)	865 (75.1)
Intervention	129 (16.7)	147 (15.1)	173 (17.2)	231 (25.7)	277 (24.5)	287 (24.9)
Total	772 (100)	973 (100)	1008 (100)	898 (100)	1129 (100)	1152 (100)
**Room temperature (^{0}C), mean (sd)**	28.7 (3.4)	30.2 (3.4)	30.1 (2.6)	28.3 (3.5)	29.8 (3.2)	29.6 (2.6)

IQR – interquartile range; n – sample size; NTLI – night-time light intensity; sd – standard deviation

[a]Characteristics are presented by thirds (low, medium and high level) of ranked NTLI

Crude and adjusted association of night-time light intensity with cardiovascular disease risk factors

Model predicted mean BMI, SBP and LDL increased with rising NTLI in crude and adjusted linear models (Fig. 2 and Tab. 2). Across the range of NTLI, the fully adjusted predicted mean increased 1.0 kg/m^2 (95% CI 0.01, 1.9, p=0.05) for BMI; and 4.2 mmHg (95% CI 1.0, 7.4, p=0.01) for SBP. The pattern was similar for LDL with

For peer review only - http://bmjopen.bmj.com/site/about/guidelines.xhtml

a fully adjusted predicted mean increase across the range of NTLI values of 0.3 mmol/l (95% CI -0.01, 0.7), although the evidence was weak from crude (p=0.06) and fully adjusted linear models (p=0.06). There were no linear association of NTLI with FPG (fully adjusted mean difference across the range of NTLI: -0.01 mmol/l (95% CI -0.4, 0.4, p=0.97).

Fig. 2 Crude associations of night-time light intensity with CVD risk factors among APCAPS adults, 2010-12 (n=5937)

APCAPS – Andhra Pradesh Children and Parents Study; BMI – body mass index; CVD – Cardiovascular disease; FPG – fasting plasma glucose; LDL – low-density lipoprotein; NTLI – night-time light intensity; SBP – systolic blood pressure

Model predicted means were derived from multilevel linear regression with clustering by household and village, using individual-level outcome data

Marker size proportional to village size

Model predicted mean SBP was on average 6.1 mmHg (95% CI 5.42, 6.9, p<0.001) higher among men than women, whereas levels of BMI, LDL and FPG did not differ by gender. However, there was no statistical evidence of gender modifying any of the associations between NTLI and the CVD risk factors. There was no evidence of gender modification of the associations of NTLI with CVD risk factors. There was strong evidence that age modified the associations of NTLI with BMI (p<0.001) and SBP (p<0.001), but not LDL, or FPG. Night-time light intensity was not associated with BMI or SBP in the younger age groups (up to 39 years), whereas the evidence and magnitudes of associations from both crude and fully adjusted models were stronger in older age groups (from 40 years) than predicted in the overall analysis (Supplementary Fig. 1 and Supplementary Tab. 1). Supplementary Fig. 2 illustrates at which levels of NTLI the fully adjusted models predicted the greatest probability of associations differing by age group. Overall and age-specific results of a supplementary analysis for SBP adjusted for room temperature at clinical assessment (n=3160) were similar to the main and age-specific SBP analyses (Supplementary Tab. 2-3 and Supplementary Fig. 3).

For peer review only - http://bmjopen.bmj.com/site/about/guidelines.xhtml

Tab. 2 Crude and adjusted associations of NTLI with CVD risk factors among APCAPS adults, 2010-12 (n=5937)

		Model predicted crude mean (95% CI) at the lowest NTLI (61.7 (4.1 on the log scale))	Model predicted crude mean (95% CI) at the highest NTLI (1081.1 (7.0 on the log scale))		Model predicted age and gender adjusted mean change (95% CI) with increasing NTLI[c]			Model predicted fully adjusted mean change (95% CI) with increasing NTLI[c, d]		
	n			p-value	n	β (95% CI)	p-value	n	β (95% CI)	p-value
Systolic blood pressure (mmHg)[a]	5752	117.2 (115.1, 119.4)	121.6 (119.2, 123.9)	0.04	5747	1.8 (0.4, 3.2)	0.01	5514	1.5 (0.3, 2.6)	0.01
Body mass index (kg/m2)	5924	20.3 (19.8, 20.8)	21.5 (21.0, 22.1)	0.01	5920	0.4 (0.1, 0.8)	0.01	5682	0.3 (0.03, 0.7)	0.05
Fasting serum LDL (mmol/l)	5287	2.4 (2.2, 2.6)	2.7 (2.5, 2.9)	0.06	5285	0.1 (0.01, 0.2)	0.04	5051	0.1 (-0.003, 0.2)	0.06
Fasting plasma glucose (mmol/l)[b]	5328	5.1 (5.0, 5.3)	5.2 (5.0, 5.4)	0.61	5326	0.04 (-0.1, 0.2)	0.52	5094	-0.002 (-0.1, 0.1)	0.97

APCAPS – Andhra Pradesh Children and Parents Study; CI - confidence interval; CVD – cardiovascular disease; LDL – low-density lipoprotein; n – sample size; NTLI – night-time light intensity; β – beta-coefficient

Model predicted means (95% CIs), β-coefficients (95% CIs) and p-values were obtained from multilevel linear regression models with clustering by household and village (using individual-level outcome data)

Participants were excluded if medicated for hypertension[a] or diabetes[b]

[c]Mean change per integer increase in log transformed NTLI

[d]Adjusted for age, gender, caste, religion, marital status and survey season

For peer review only

For peer review only - http://bmjopen.bmj.com/site/about/guidelines.xhtml

Comparing included and excluded participants

Participants, who were excluded from the main analysis due to missing data, medication use, or high triglyceride levels differed in some respects from the analysed participants. Excluded individuals were on average older, less likely to attend clinical assessment/interview in the summer, and had higher levels of the four CVD risk factors than included individuals.

Sensitivity analyses

A sensitivity analysis among HNT index children (n=1245 (21% of overall sample); intervention n=622, control n=623) did not indicate confounding or interaction by intervention status. In a second sensitivity analysis we removed three borderline outlier villages identified in analyses of BMI, SBP and FPG. The results of this sensitivity analysis were consistent with the overall and age-stratified main analyses for BMI, SBP and FPG (Supplementary Fig. 4-5 and Supplementary Tab. 4-5). However, results for SBP (not adjusted for room temperature) were not statistically significant at the 95% level. Supplementary Fig. 6 illustrates at which levels of NTLI the fully adjusted sensitivity analysis predicted the greatest probability of differences in associations of NTLI and CVD risk factors by age group.

DISCUSSION

Among 6236 participants, NTLI was associated with increased model predicted mean BMI, SBP and serum fasting LDL, but not FPG after controlling for confounders. We expected younger individuals to more readily adopt new lifestyles and behaviours associated with urbanisation and CVD risk factors than older individuals.[9 44] This was however not apparent from the data. The observed stronger associations of NTLI with BMI and SBP in older age groups were likely attributable to advancing age.[32] Gender did not modify associations.

Comparison with studies using night-time light intensity

Few studies have used NTLI data to explore relationships of urbanisation and CVD risk factors. A cross-sectional study set in rural and urban Korea (n=8526)[46] and a multi-country study of nationally representative data (n=130 counties),[47] investigated associations of NTLI with BMI. Consistent with our results on BMI, increasing level of NTLI was associated with rising levels of overweight and obesity in both studies. A cross-sectional study from India, which analysed NTLI data from NASA's Visible Infrared Imaging Radiometer Suite,[48] corroborates our adjusted results for SBP, although the included set of covariates differed between our studies. We did not find any studies on relationships between NTLI and LDL or FPG.

For peer review only - http://bmjopen.bmj.com/site/about/guidelines.xhtml

Comparison with studies using multi-component urbanisation scores

Our findings on BMI and SBP agree with cross-sectional studies from South India[15] (n=3705) and Sri Lanka (n=4485),[18] which used multi-component scores to estimate urbanisation levels in multi province/state representative samples. Although we cannot compare our results directly to studies that use urbanisation scores (as opposed to NTLI) the direction of effects from low to medium (and high) urbanisation levels are consistent with our findings from an early stage urbanisation population, and support evidence of an association. We found no studies exploring relationships between urbanisation score and FPG, whereas findings from Sri Lanka[18] and China[49] suggest a greater likelihood of diabetes at high but not medium level of urbanisation score (ranging from rural areas to large city). It is possible that we did not observe an association of urbanisation level with FPG in our study because adverse effects of urbanisation on FPG do not manifest until a medium level of urbanisation is exceeded. However, restricting our analysis to non-diabetics may also have contributed to the null results for FPG, particularly as excluded individuals were on average older and had higher BMI.

Comparison with rural-urban and migrant studies

Measuring urbanisation is challenging, particularly in settings where population and environment data are sparse.[22] As a result, relationships of urbanisation and CVD risk factors are often inferred from rural-urban comparisons and migrant studies. Several of these studies focus on or include Asian populations. An Indian migration study of 3537 sibling pairs suggests that mean BMI is lower among rural than migrant and urban populations. Patterns were similar for mean SBP, LDL, and FPG among men but not women.[45] In a different analysis of the same study, cumulative exposure to urban environments additionally increased mean BMI of both genders as well as SBP and fasting glucose of men, while LDL remained unaffected.[13] Systolic blood pressure (as the only outcome) was on average higher among men than women in our sample, however gender did not modify associations. A cross-sectional study from Southern India suggests that mean BMI, SBP, and the prevalence of diabetes were lower among peri-urban residents of both genders than among town and city dwellers.[50] Urban versus rural residence was also associated with higher prevalence of diabetes[49] and mean LDL in China[51] after adjusting for important confounders. Two large studies from 56[8] and 36[52] countries (n=878000 and 148579 respectively) across Africa, the Middle East, Asia, Americas, the Caribbean and USA suggest that overweight (BMI $\geq$25 kg/m^2) is considerably more common among urban than rural women, particularly in lower income countries.[8] In a subsample from India (n=7608), the prevalence of overweight was almost five fold among urban (26.4 %) compared to rural women (5.6 %).[52]

For peer review only

For peer review only - http://bmjopen.bmj.com/site/about/guidelines.xhtml

Strengths and limitation

A major contribution of our study to the existing evidence was exploring CVD risk factors in a transitioning population in early stage urbanisation, i.e. 27 rural villages that have experienced rapid and uneven urbanisation over time and thus represented a continuum of urbanisation levels from rural settlement to medium size town at the time of survey in 2010-2012. The village-level NTLI has been validated as a standardised and comparable proxy of urbanisation level, reflecting extent of build-up area, population density, economic activity, and energy consumption world-wide.[24-27] It may further be a more objective measure than the survey based data used for multiple-component urbanisation scales, which are at risk of both interviewer and reporting biases. The NTLI data have gained popularity for exploring associations of urbanisation level and artificial light at night with human health, particularly cancer,[53-61] overweight and obesity.[46 47 62] The NTLI time series has further been suggested a valid proxy for urbanisation dynamics over time,[24 27] especially in rapidly developing countries with high urban growth rates such as China, India, and Brazil.[27] Recent efforts to calibrate the NTLI time series has shown great promise for studying urbanisation dynamics in these settings[28] and offer a unique resource for health research worldwide. We calibrated the NTLI data using a robust and reliable semiautomatic method to reduce systematic bias from satellite discrepancies and enable more efficient and precise comparisons of results between studies from different regions and time points.[28] However, the semiautomatic method could introduce new bias from manual processing.[28] The overall agreement of our result with existing evidence (using different measures of urbanisation level) supports the utility of NTLI to predict urbanisation-related changes in CVD risk factors. An important limitation of our study was use of a village level exposure with individual level outcomes. Future studies could reduce the potential for bias related to the ecological fallacy by allowing for intra-settlement variations in NTLI. The large sample size and use of hierarchical statistical methods should reduce overestimation of the magnitude of associations from risk factor clustering by sampling units (villages and households). The comparisons of excluded and included participants did however warranted some caution of generalising the results to the general population. Trained field and clinical staff followed standardised protocols and collected information in the local language. A comprehensive list of factors were measured and explored as potential confounders and effect modifiers in our study, however, some residual confounding from unmeasured factors may remain. Data for a number of covariates were self-reported, and some recall or reporting bias cannot be rule out. We did not adjust our results for socio-demographic factors such as socio-economic status, education and occupation because these were considered important factors on the causal pathway between urbanisation and CVD risk factors in the current population, who remained at early stages of urbanisation. Most

For peer review only - http://bmjopen.bmj.com/site/about/guidelines.xhtml

urbanisation in India has predominantly been driven by the reclassification of rural settlements as they grow and develop.[38] Although adversity in rural areas, for example poor opportunities for education and paid work, also contribute to urban growth through internal migration in India, the migration flow is mainly from rural to highly urbanised areas.[38 63] The 27 villages analysed in the current study remained at early stages of urbanisation and represented a continuum from rural settlement to medium sized town. We hypothesised that participants, who for example pursued better education or work opportunities, migrated out of the study area (to urban areas or abroad) were lost to follow-up and did not confound the results by migrating to higher urbanisation level villages within the study area. We believe that the contribution of socio-demographic factors, such as socio-economic status, education and occupation, to the association of urbanisation on CVD risk factors in populations in early stage urbanisation should be investigated using causal methods. Finally, the cross-sectional design of the present study prevented us from inferring causality of the results as well as from exploring the relative contribution of different urbanisation elements to CVD risk.

Hypothesised pathways linking urbanisation and CVD risk factors

There are a number of plausible pathways through which urbanisation may deteriorate cardiovascular health. Physical and build environments change with urbanisation and in turn influence the level of exposure to noise and air pollution, green spaces and infrastructure.[22 48 64 65] Urbanisation is also associated with changes in occupation patterns and socio-economic status[15 64]; social norms, cohesion, and support; as well as personal tastes and preferences.[22 48 65] Together with increased availability, access and advertising of commodities, these transitions promote lifestyle changes that are strongly associated with CVD risk, e.g. 'nutrition transition' towards diets high in fat, sugar and salt as well as processed and convenience foods; physical inactivity during work, leisure time, transport and household chores; tobacco use and alcohol consumption.[9 18 22 64 66] Cumulative exposure to urban environments additionally appears to increase CVD risk.[13 17] Recent studies suggest that increased exposure to artificial light at night observed with increasing urbanisation level supresses melatonin production, disrupts circadian rhythm, and leads to physiological and behavioural changes associated with CVD.[47] A number of studies focusing on artificial light at night (other than NTLI) without accounting for urbanisation support this mechanism.[54 67-72] At the same time, greater level of urbanisation is also associated with a number of changes that may improve CVD health. Greater level of urbanisation is associated with greater levels and higher quality of education; improved opportunities for paid work, particularly for women; higher socio-economic status and better living conditions; better access to health, social services and safe food, which have great potential to improve cardiovascular health.[73-78] Although these pathways have been studied

For peer review only - http://bmjopen.bmj.com/site/about/guidelines.xhtml

extensively, no study has to our knowledge attempted to quantify their relative harmful and beneficial contributions.

Implications

Our findings indicate that the harmful impacts of increasing urbanisation level on SBP and BMI, well-established leading modifiable risk factors for CVDs, outweigh any beneficial impacts even with moderate increases in urbanisation level. These findings suggest an opportunity for policy makers and urban planners to help curb expected increases in urbanisation-related CVDs by addressing underlying and immediate risk factors for elevated SBP and BMI during early stages of urbanisation. We will however need to understand better and quantify the implicated pathways in different settings in order to identify cost-effective prevention strategies.

CONCLUSION

We found that the NTLI data, a novel measure of urbanisation, is a useful tool to predict changes in important urbanisation-related CVD risk factors where objective data on urbanisation factors are not readily available. The observed consistent associations of moderate increases in urbanisation level (measured by NTLI) with CVD risk factors provide an important early warning for the potential progression of the CVD burden in South India with continued urbanisation. To curb expected large public health impacts from continued and rapid urbanisation in India, further research is warranted to identify the most important mechanisms. To enable targeting of cost-effective prevention strategies, we suggest that future studies focus on transitioning populations and use causal methods to better understand potentially important pathways.

ABBREVIATIONS

APCAPS	Andhra Pradesh Children and Parents Study
BMI	body mass index
CI	confidence interval
CVD	cardiovascular disease
FPG	fasting plasma glucose
HNT	Hyderabad Nutrition Trial

For peer review only

IQR	interquartile range
LDL	low-density lipoprotein
LMIC	low- and middle-income country
LSHTM	London School of Hygiene and Tropical Medicine
n	sample size
NTLI	night-time light intensity
SBP	systolic blood pressure
UK	United Kingdom
β	Beta-coefficient

DECLARATIONS

Ethics

Ethics approvals were obtained from Public Health Foundation of India, New Delhi, India; National Institute of Nutrition, Hyderabad, India; and the London School of Hygiene and Tropical Medicine (LSHTM), London, UK. Approvals were obtained from all village heads and their committees. Study participants provided written informed consent, or a witnessed thumbprint if illiterate, prior to study start.

Consent for publication

Study participants provided written informed consent, or a witnessed thumbprint if illiterate, for the use and publication of their anonymised data in accordance with the study objectives.

Availability of data and materials

The dataset analysed in the current study is available from the APCAPS (http://apcaps.lshtm.ac.uk/) upon reasonable request.

For peer review only

Competing interests

The authors declare that they have no competing interests.

Role of the funding source

This work was supported by Wellcome Trust (www.wellcome.ac.uk/) Grant number 083707 and the Bloomsbury PhD Studentship (http://www.bloomsbury.ac.uk/) through LSHTM, London, UK.

Author contributions

All authors (TBS, RW, JG, BS, ADD, SK) contributed substantially to study conception and manuscript revision; and approved the final manuscript for publication. TBS and JG cleaned and managed the Andhra Pradesh Children and Parents Study (APCAPS) data in collaboration with the APCAPS team. RW extracted, calibrated and managed the night-time light intensity data; JG advised on statistical methods. TBS conducted the literature search, data analysis and interpretation, and drafted the manuscript; SK is the director of the APCAPS.

Acknowledgements

We would like to thank the APCAPS team and support staff, in particular (in alphabetical order by surname) Srivalli Addanki, Christopher Baker, Santhi Bhogadi, Naveen Chittaluri, Ruby Gupta, **Bharati Kulkarni**, Poppy Mallinson, Sandeep Masapeta, Subhash Masapeta, and Chitra Sharma.

REFERENCES

1. India State-level Disease Burden Initiative Collaborators. Nations within a nation: variations in epidemiological transition across the states of India, 1990-2016 in the Global Burden of Disease Study. *Lancet* 2017 doi: 10.1016/s0140-6736(17)32804-0 [published Online First: 2017/11/19]
2. Prabhakaran D, Jeemon P, Roy A. Cardiovascular Diseases in India: Current Epidemiology and Future Directions. *Circulation* 2016;133(16):1605-20. doi: 10.1161/circulationaha.114.008729 [published Online First: 2016/05/05]
3. Patel V, Chatterji S, Chisholm D, et al. Chronic diseases and injuries in India. *Lancet* 2011;377(9763):413-28. doi: 10.1016/s0140-6736(10)61188-9 [published Online First: 2011/01/14]
4. Yusuf S, Rangarajan S, Teo K, et al. Cardiovascular risk and events in 17 low-, middle-, and high-income countries. *The New England journal of medicine* 2014;371(9):818-27. doi: 10.1056/NEJMoa1311890 [published Online First: 2014/08/28]
5. Gupta R, Joshi P, Mohan V, et al. Epidemiology and causation of coronary heart disease and stroke in India. *Heart (British Cardiac Society)* 2008;94(1):16-26. doi: 10.1136/hrt.2007.132951 [published Online First: 2007/12/18]
6. Sharma M, Ganguly NK. Premature coronary artery disease in Indians and its associated risk factors. *Vascular health and risk management* 2005;1(3):217-25. [published Online First: 2007/02/27]

For peer review only

7. Prabhakaran D, Singh K. Premature coronary heart disease risk factors & reducing the CHD burden in India. *The Indian journal of medical research* 2011;134:8-9. [published Online First: 2011/08/03]
8. Goryakin Y, Suhrcke M. Economic development, urbanization, technological change and overweight: what do we learn from 244 Demographic and Health Surveys? *Econ Hum Biol* 2014;14:109-27. doi: 10.1016/j.ehb.2013.11.003 [published Online First: 2014/01/25]
9. Popkin BM, Adair LS, Ng SW. Global nutrition transition and the pandemic of obesity in developing countries. *Nutr Rev* 2012;70(1):3-21. doi: 10.1111/j.1753-4887.2011.00456.x [published Online First: 2012/01/10]
10. Allender S, Foster C, Hutchinson L, et al. Quantification of urbanization in relation to chronic diseases in developing countries: a systematic review. *Journal of urban health : bulletin of the New York Academy of Medicine* 2008;85(6):938-51. doi: 10.1007/s11524-008-9325-4 [published Online First: 2008/10/22]
11. Vlahov D, Galea S. Urbanization, urbanicity, and health. *Journal of urban health : bulletin of the New York Academy of Medicine* 2002;79(4 Suppl 1):S1-s12. [published Online First: 2002/12/11]
12. Moore M, Gould P, Keary BS. Global urbanization and impact on health. *International journal of hygiene and environmental health* 2003;206(4):269-78. doi: https://doi.org/10.1078/1438-4639-00223
13. Kinra S, Andersen E, Ben-Shlomo Y, et al. Association between urban life-years and cardiometabolic risk: the Indian migration study. *American journal of epidemiology* 2011;174(2):154-64. doi: 10.1093/aje/kwr053 [published Online First: 2011/05/31]
14. Bowen L, Ebrahim S, De Stavola B, et al. Dietary intake and rural-urban migration in India: a cross-sectional study. *PLoS One* 2011;6(6):e14822. doi: 10.1371/journal.pone.0014822 [published Online First: 2011/07/07]
15. Allender S, Lacey B, Webster P, et al. Level of urbanization and noncommunicable disease risk factors in Tamil Nadu, India. *Bull World Health Organ* 2010;88(4):297-304. doi: 10.2471/blt.09.065847 [published Online First: 2010/05/01]
16. Subramanian SV, Smith GD. Patterns, distribution, and determinants of under- and overnutrition: a population-based study of women in India. *The American journal of clinical nutrition* 2006;84(3):633-40. [published Online First: 2006/09/09]
17. Sobngwi E, Mbanya JC, Unwin NC, et al. Exposure over the life course to an urban environment and its relation with obesity, diabetes, and hypertension in rural and urban Cameroon. *International journal of epidemiology* 2004;33(4):769-76. doi: 10.1093/ije/dyh044 [published Online First: 2004/05/29]
18. Allender S, Wickramasinghe K, Goldacre M, et al. Quantifying urbanization as a risk factor for noncommunicable disease. *Journal of urban health : bulletin of the New York Academy of Medicine* 2011;88(5):906-18. doi: 10.1007/s11524-011-9586-1 [published Online First: 2011/06/04]
19. Torun B, Stein AD, Schroeder D, et al. Rural-to-urban migration and cardiovascular disease risk factors in young Guatemalan adults. *International journal of epidemiology* 2002;31(1):218-26. [published Online First: 2002/03/27]
20. Poulter NR, Khaw KT, Hopwood BE, et al. The Kenyan Luo migration study: observations on the initiation of a rise in blood pressure. *BMJ (Clinical research ed)* 1990;300(6730):967-72. [published Online First: 1990/04/14]
21. Al-Moosa S, Allin S, Jemiai N, et al. Diabetes and urbanization in the Omani population: an analysis of national survey data. *Population health metrics* 2006;4:5. doi: 10.1186/1478-7954-4-5 [published Online First: 2006/04/26]
22. Dahly DL, Adair LS. Quantifying the urban environment: a scale measure of urbanicity outperforms the urban-rural dichotomy. *Social science & medicine (1982)* 2007;64(7):1407-19. doi: 10.1016/j.socscimed.2006.11.019

For peer review only - http://bmjopen.bmj.com/site/about/guidelines.xhtml

23. Knowledge Network on Urban Settings. Our cities, our health, our future. Kobe City, Japan: World Health Organization, 2008.
24. Yi K, Tani H, Li Q, et al. Mapping and evaluating the urbanization process in Northeast China using DMSP/OLS nighttime light data. *Sensors (Basel, Switzerland)* 2014;14(2):3207-26. doi: 10.3390/s140203207 [published Online First: 2014/02/21]
25. Ma T, Zhou C, Pei T, et al. Quantitative estimation of urbanization dynamics using time series of DMSP/OLS nighttime light data: A comparative case study from China's cities. *Remote Sensing of Environment* 2012;124(Supplement C):99-107. doi: https://doi.org/10.1016/j.rse.2012.04.018
26. Imhoff M, Lawrence W, Stutzer D, et al. A technique for using composite DMSP/OLS "City Lights" satellite data to map urban area. *Remote Sensing of Environment* 1997;61(3):361-70. doi: https://doi.org/10.1016/S0034-4257(97)00046-1
27. Zhang Q, Seto KC. Mapping urbanization dynamics at regional and global scales using multi-temporal DMSP/OLS nighttime light data. *Remote Sensing of Environment* 2011;115(9):2320-29. doi: https://doi.org/10.1016/j.rse.2011.04.032
28. Zhang Q, Pandey B, Seto KC. A Robust Method to Generate a Consistent Time Series From DMSP/OLS Nighttime Light Data. *IEEE Transactions on geoscience and remote sensisng* 2016 doi: 10.1109/TGRS.2016.2572724
29. Andhra Pradesh Children and Parents Study (APCAPS). Andhra Pradesh Children and Parents Study: London School of Hygiene and Tropical Medicine; 2018 [Available from: http://apcaps.lshtm.ac.uk/ accessed 12 July 2018.
30. Kinra S, Radha Krishna K, Kuper H, et al. Cohort Profile: Andhra Pradesh Children and Parents Study (APCAPS). *International journal of epidemiology* 2013 doi: 10.1093/ije/dyt128 [published Online First: 2013/09/11]
31. Kinra S, Rameshwar Sarma KV, Ghafoorunissa, et al. Effect of integration of supplemental nutrition with public health programmes in pregnancy and early childhood on cardiovascular risk in rural Indian adolescents: long term follow-up of Hyderabad nutrition trial. *BMJ (Clinical research ed)* 2008;337:a605. doi: 10.1136/bmj.a605 [published Online First: 2008/07/29]
32. National Cholesterol Education Program, National Heart L, and Blood Institute,, National Institutes of Health. Third Report of the National Cholesterol Education Program (NCEP) Expert Panel on Detection, Evaluation, and Treatment of High Blood Cholesterol in Adults (Adult Treatment Panel III). NIH Publication, 2002.
33. National Oceanic and Atmospheric Administration National Centers for Environmental Information. Earth Observation Group (EOG). Defense Meteorological Satellite Program (DMSP) Data Archive, Research, and Products: National oceanic and atmospheric administration; [Available from: https://www.ngdc.noaa.gov/eog/dmsp/dmsp.html accessed 13 July 2020.
34. Google Earth Engine. Earth Engine Data Catalog. DMSP OLS: Nighttime Lights Time Series Version 4, Defense Meteorological Program Operational Linescan System na [Available from: https://developers.google.com/earth-engine/datasets/catalog/NOAA_DMSP-OLS_NIGHTTIME_LIGHTS accessed 22 October 2019.
35. Elvidge CD, Imhoff ML, Baugh KE, et al. Nighttime lights of the world: 1994-95 *ISPRS Journal of Photogrammetry and Remote Sensing* 2001;56:81-99.
36. Organisation for Economic Co-operation and Development. Glossary of statistical terms: Urbanization 2003 [Available from: https://stats.oecd.org/glossary/detail.asp?ID=2819.
37. United Nations DoEaSA, Population Division,. World Urbanization Prospects: The 2018 Revision, Methodology. New York: United Nation, 2018.
38. International Organization for Migration UN Migration. World Migration Report 2020. Geneva, Switzerland, 2019.

For peer review only - http://bmjopen.bmj.com/site/about/guidelines.xhtml

39. GBD 2015 Risk Factors Collaborators. Global, regional, and national comparative risk assessment of 79 behavioural, environmental and occupational, and metabolic risks or clusters of risks, 1990-2015: a systematic analysis for the Global Burden of Disease Study 2015. *Lancet* 2016;388(10053):1659-724. doi: 10.1016/s0140-6736(16)31679-8 [published Online First: 2016/10/14]
40. Friedewald WT, Levy RI, Fredrickson DS. Estimation of the concentration of low-density lipoprotein cholesterol in plasma, without use of the preparative ultracentrifuge. *Clinical chemistry* 1972;18(6):499-502. [published Online First: 1972/06/01]
41. Kinra S, Johnson M, Kulkarni B, et al. Socio-economic position and cardiovascular risk in rural indian adolescents: evidence from the Andhra Pradesh children and parents study (APCAPS). *Public health* 2014;128(9):852-9. doi: 10.1016/j.puhe.2014.06.005
42. Belloni A, Chernozhukov V, Hansen C. Inference on Treatment Effects after Selection among High-Dimensional Controls. *The Review of Economic Studies* 2014;81(2):608-50. doi: 10.1093/restud/rdt044
43. Tacoli C. Urbanization and emerging population issues working paper 7. Urbanization, gender and urban poverty: paid work and unpaid carework in the city: United Nations Population Fund, International Institute for Environment and Development,, 2012:48.
44. Poobalan A, Aucott L. Obesity Among Young Adults in Developing Countries: A Systematic Overview. *Current Obesity Reports* 2016;5:2-13. doi: 10.1007/s13679-016-0187-x
45. Ebrahim S, Kinra S, Bowen L, et al. The effect of rural-to-urban migration on obesity and diabetes in India: a cross-sectional study. *PLoS medicine* 2010;7(4):e1000268. doi: 10.1371/journal.pmed.1000268 [published Online First: 2010/05/04]
46. Koo YS, Song JY, Joo EY, et al. Outdoor artificial light at night, obesity, and sleep health: Cross-sectional analysis in the KoGES study. *Chronobiology international* 2016;33(3):301-14. doi: 10.3109/07420528.2016.1143480 [published Online First: 2016/03/08]
47. Rybnikova NA, Haim A, Portnov BA. Does artificial light-at-night exposure contribute to the worldwide obesity pandemic? *International journal of obesity (2005)* 2016;40(5):815-23. doi: 10.1038/ijo.2015.255 [published Online First: 2016/01/23]
48. Lane KJ, Stokes EC, Seto KC, et al. Associations between Greenness, Impervious Surface Area, and Nighttime Lights on Biomarkers of Vascular Aging in Chennai, India. *Environmental health perspectives* 2017;125(8):087003. doi: 10.1289/ehp541 [published Online First: 2017/09/09]
49. Attard SM, Herring AH, Mayer-Davis EJ, et al. Multilevel examination of diabetes in modernising China: what elements of urbanisation are most associated with diabetes? *Diabetologia* 2012;55(12):3182-92. doi: 10.1007/s00125-012-2697-8 [published Online First: 2012/08/28]
50. Ramachandran A, Mary S, Yamuna A, et al. High prevalence of diabetes and cardiovascular risk factors associated with urbanization in India. *Diabetes care* 2008;31(5):893-8. doi: 10.2337/dc07-1207 [published Online First: 2008/03/04]
51. He J, Klag MJ, Wu Z, et al. Effect of migration and related environmental changes on serum lipid levels in southwestern Chinese men. *American journal of epidemiology* 1996;144(9):839-48. [published Online First: 1996/11/01]
52. Mendez MA, Monteiro CA, Popkin BM. Overweight exceeds underweight among women in most developing countries. *The American journal of clinical nutrition* 2005;81(3):714-21. [published Online First: 2005/03/10]
53. Kloog I, Haim A, Stevens RG, et al. Global co-distribution of light at night (LAN) and cancers of prostate, colon, and lung in men. *Chronobiology international* 2009;26(1):108-25. doi: 10.1080/07420520802694020 [published Online First: 2009/01/15]
54. Cho Y, Ryu SH, Lee BR, et al. Effects of artificial light at night on human health: A literature review of observational and experimental studies applied to exposure assessment. *Chronobiology international* 2015;32(9):1294-310. doi: 10.3109/07420528.2015.1073158 [published Online First: 2015/09/17]

For peer review only - http://bmjopen.bmj.com/site/about/guidelines.xhtml

55. Rybnikova NA, Haim A, Portnov BA. Is prostate cancer incidence worldwide linked to artificial light at night exposures? Review of earlier findings and analysis of current trends. *Archives of environmental & occupational health* 2017;72(2):111-22. doi: 10.1080/19338244.2016.1169980 [published Online First: 2016/04/01]
56. Kloog I, Haim A, Stevens RG, et al. Light at night co-distributes with incident breast but not lung cancer in the female population of Israel. *Chronobiology international* 2008;25(1):65-81. doi: 10.1080/07420520801921572 [published Online First: 2008/02/23]
57. Hurley S, Goldberg D, Nelson D, et al. Light at night and breast cancer risk among California teachers. *Epidemiology (Cambridge, Mass)* 2014;25(5):697-706. doi: 10.1097/ede.0000000000000137 [published Online First: 2014/07/26]
58. James P, Bertrand KA, Hart JE, et al. Outdoor Light at Night and Breast Cancer Incidence in the Nurses' Health Study II. *Environmental health perspectives* 2017;125(8):087010. doi: 10.1289/ehp935 [published Online First: 2017/09/09]
59. Kloog I, Stevens RG, Haim A, et al. Nighttime light level co-distributes with breast cancer incidence worldwide. *Cancer causes & control : CCC* 2010;21(12):2059-68. doi: 10.1007/s10552-010-9624-4 [published Online First: 2010/08/04]
60. Bauer SE, Wagner SE, Burch J, et al. A case-referent study: light at night and breast cancer risk in Georgia. *International Journal of Health Geographics* 2013;12:23. doi: 10.1186/1476-072x-12-23 [published Online First: 2013/04/19]
61. Rybnikova N, Haim A, Portnov BA. Artificial Light at Night (ALAN) and breast cancer incidence worldwide: A revisit of earlier findings with analysis of current trends. *Chronobiology international* 2015;32(6):757-73. doi: 10.3109/07420528.2015.1043369 [published Online First: 2015/06/24]
62. Touitou Y, Reinberg A, Touitou D. Association between light at night, melatonin secretion, sleep deprivation, and the internal clock: Health impacts and mechanisms of circadian disruption. *Life sciences* 2017;173:94-106. doi: 10.1016/j.lfs.2017.02.008 [published Online First: 2017/02/20]
63. Government of India. Census of India 2001. Data highlights. Migration Tables (D1, D1 (Appendix), D2 and D3 Tables), 2001.
64. Tacoli C. Urbainzation, gender and poverty. New York, USA: United Nations Population Fund, International Institute for Environment and Development, 2012.
65. de Vries S, van Dillen SM, Groenewegen PP, et al. Streetscape greenery and health: stress, social cohesion and physical activity as mediators. *Soc Sci Med* 2013;94:26-33. doi: 10.1016/j.socscimed.2013.06.030 [published Online First: 2013/08/13]
66. Shetty PS. Nutrition transition in India. *Public health nutrition* 2002;5(1A):175-82. doi: 10.1079/phn2001291 [published Online First: 2002/05/25]
67. Hurley S, Nelson DO, Garcia E, et al. A cross-sectional analysis of light at night, neighborhood sociodemographics and urinary 6-sulfatoxymelatonin concentrations: implications for the conduct of health studies. *International Journal of Health Geographics* 2013;12:39. doi: 10.1186/1476-072x-12-39
68. McFadden E, Jones ME, Schoemaker MJ, et al. The relationship between obesity and exposure to light at night: cross-sectional analyses of over 100,000 women in the Breakthrough Generations Study. *American journal of epidemiology* 2014;180(3):245-50. doi: 10.1093/aje/kwu117 [published Online First: 2014/05/31]
69. Gangwisch JE. Invited commentary: nighttime light exposure as a risk factor for obesity through disruption of circadian and circannual rhythms. *American journal of epidemiology* 2014;180(3):251-3. doi: 10.1093/aje/kwu119 [published Online First: 2014/05/31]
70. Stevens RG. Light-at-night, circadian disruption and breast cancer: assessment of existing evidence. *International journal of epidemiology* 2009;38(4):963-70. doi: 10.1093/ije/dyp178 [published Online First: 2009/04/22]

For peer review only - http://bmjopen.bmj.com/site/about/guidelines.xhtml

71. Obayashi K, Saeki K, Iwamoto J, et al. Association between light exposure at night and nighttime blood pressure in the elderly independent of nocturnal urinary melatonin excretion. *Chronobiology international* 2014;31(6):779-86. doi: 10.3109/07420528.2014.900501 [published Online First: 2014/03/29]
72. Obayashi K, Saeki K, Iwamoto J, et al. Effect of exposure to evening light on sleep initiation in the elderly: a longitudinal analysis for repeated measurements in home settings. *Chronobiology international* 2014;31(4):461-7. doi: 10.3109/07420528.2013.840647 [published Online First: 2013/10/24]
73. Ezzati M, Webster CJ, Doyle YG, et al. Cities for global health. 2018;363:k3794. doi: 10.1136/bmj.k3794 %J BMJ
74. World Health Organization. Urbanization and health 2019 [Available from: https://www.who.int/bulletin/volumes/88/4/10-010410/en/ accessed 16 July 2019.
75. Vlahov D, Freudenberg N, Proietti F, et al. A conceptual framework for organizing determinants of urban health. Thematic paper 1 for Knowledge Network on Urban Settings WHO Centre for Health Development, 2008.
76. Satterthwaite D, McGranahan G, Tacoli C. Urbanization and its implications for food and farming. *Philosophical transactions of the Royal Society of London Series B, Biological sciences* 2010;365(1554):2809-20. doi: 10.1098/rstb.2010.0136 [published Online First: 2010/08/18]
77. Estache A, Garsous G. The scope for an impact of infrastructure investments on jobs in developing countries International Finance Corporation, 2012.
78. Eckert S, Kohler S. Urbanization and health in developing countries: a systematic review. *World health & population* 2014;15(1):7-20. [published Online First: 2014/04/08]

FIGURE TITLES AND LEGENDS

Fig. 1 Sampling and participant flow for the third survey wave of the APCAPS

APCAPS – Andhra Pradesh Children and Parents Study; BMI – body mass index; FPG – fasting plasma glucose; HNT – Hyderabad Nutrition Trial; LDL – low-density lipoprotein; SBP – systolic blood pressure

* The Hyderabad Nutrition Trial (1987-1990) included 15 intervention and 14 control villages from the Integrated Child Development Services Scheme (stepped-wedge cluster randomised nutrition supplementation trial)

Fig. 2 Crude associations of night-time light intensity with CVD risk factors among APCAPS adults, 2010-12 (n=5937)

APCAPS – Andhra Pradesh Children and Parents Study; BMI – body mass index; CVD – Cardiovascular disease; FPG – fasting plasma glucose; LDL – low-density lipoprotein; NTLI – night-time light intensity; SBP – systolic blood pressure

Model predicted means were derived from multilevel linear regression with clustering by household and village, using individual-level outcome data

Marker size proportional to village size

For peer review only - http://bmjopen.bmj.com/site/about/guidelines.xhtml

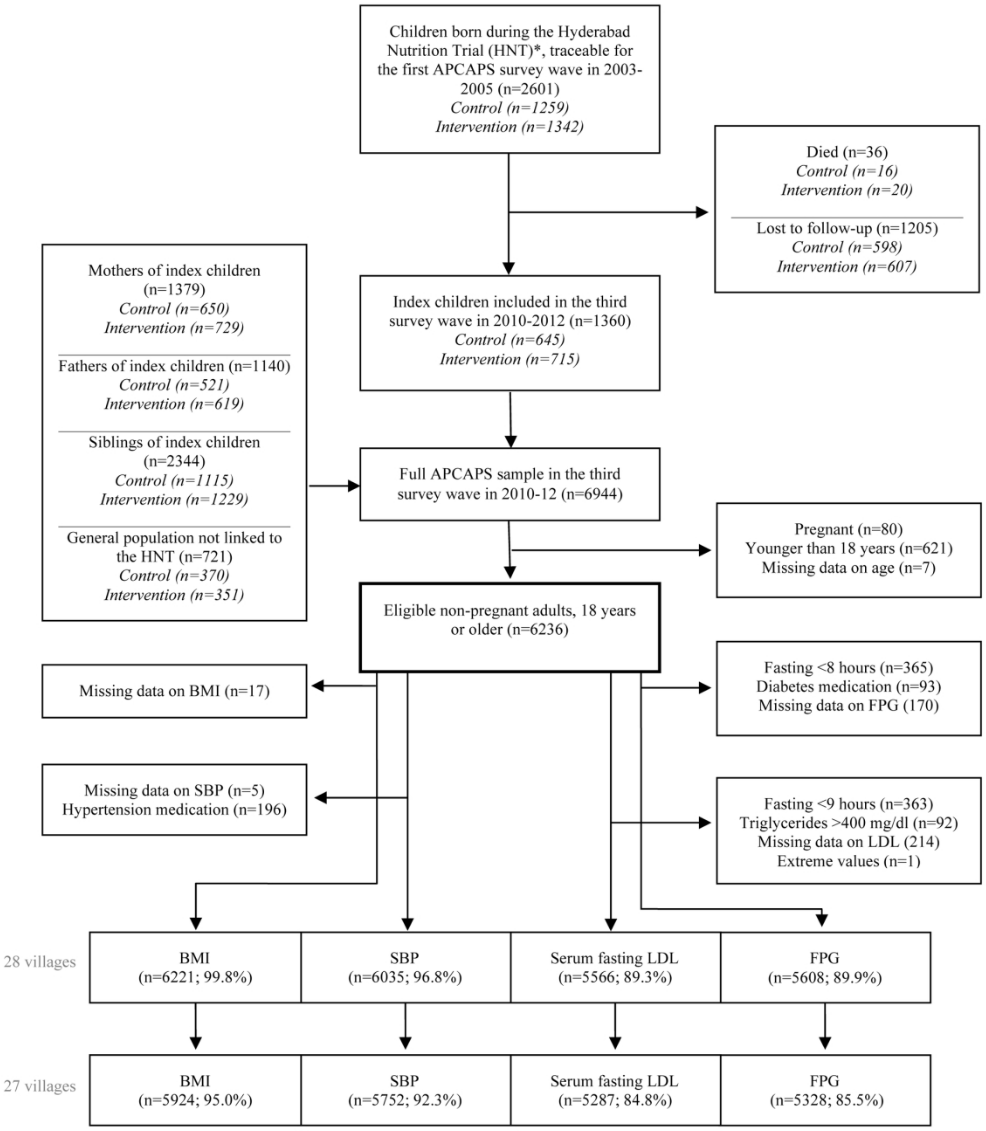

Fig. 1 Sampling and participant flow for the third survey wave of the APCAPS
APCAPS – Andhra Pradesh Children and Parents Study; BMI – body mass index; FPG – fasting plasma glucose; HNT – Hyderabad Nutrition Trial; LDL – low-density lipoprotein; SBP – systolic blood pressure
* The Hyderabad Nutrition Trial (1987-1990) included 15 intervention and 14 control villages from the Integrated Child Development Services Scheme (stepped-wedge cluster randomised nutrition supplementation trial)

170x192mm (300 x 300 DPI)

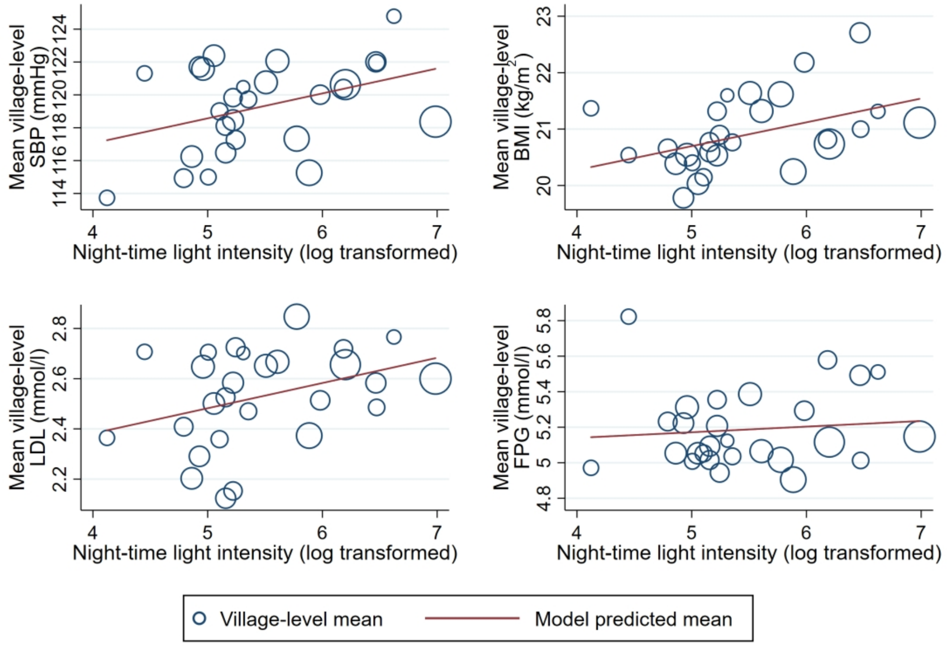

Fig. 2 Crude associations of night-time light intensity with CVD risk factors among APCAPS adults, 2010-12 (n=5937)
APCAPS – Andhra Pradesh Children and Parents Study; BMI – body mass index; CVD – Cardiovascular disease; FPG – fasting plasma glucose; LDL – low-density lipoprotein; NTLI – night-time light intensity; SBP – systolic blood pressure
Model predicted means were derived from multilevel linear regression with clustering by household and village, using individual-level outcome data
Marker size proportional to village size

881x641mm (72 x 72 DPI)

SUPPLEMENTARY FILES

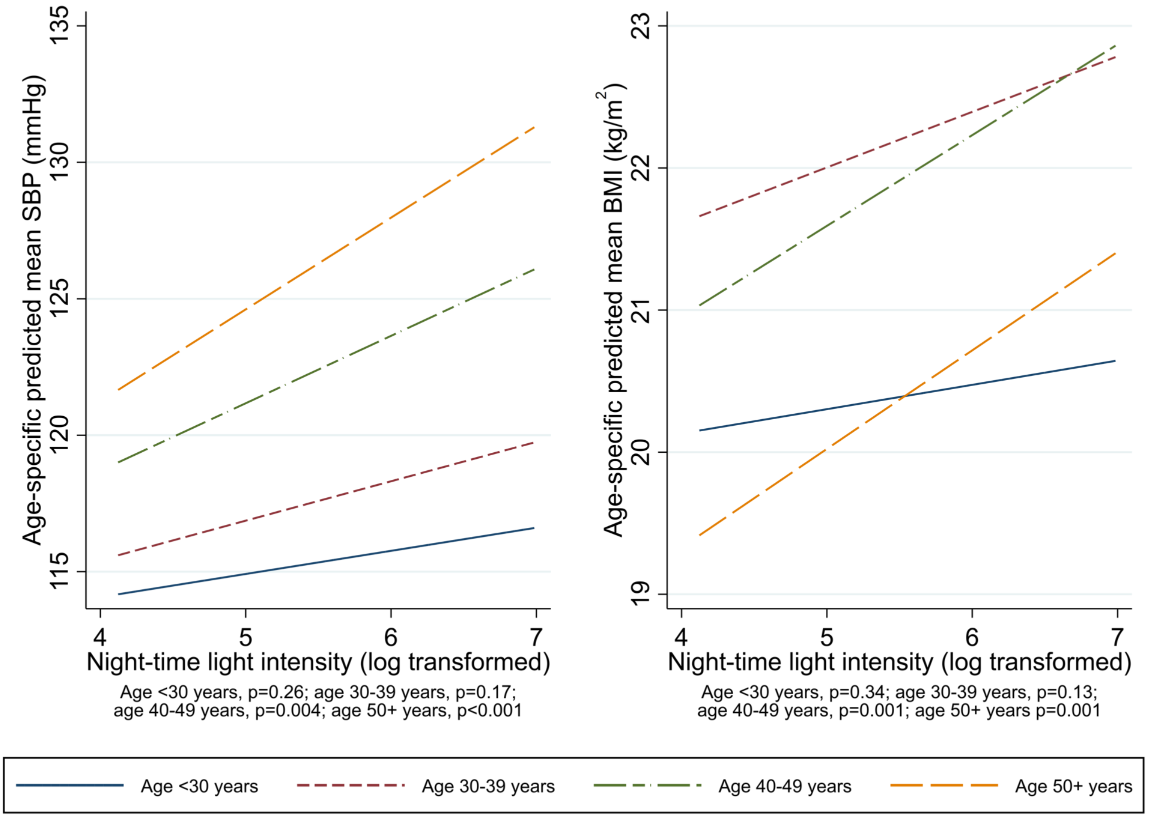

Supplementary Fig. 1 Age-specific crude associations of NTLI with mean BMI and SBP, APCAPS, 2010-12 (n=5937)

APCAPS – Andhra Pradesh Children and Parents Study; BMI – body mass index; NTLI – night-time light intensity; SBP – systolic blood pressure

The fitted lines (model predicted means) were derived from crude multilevel linear regression models with clustering by household and village, using individual-level outcome data

Participants were excluded from analysis of SBP if medicated for hypertension

For peer review only - http://bmjopen.bmj.com/site/about/guidelines.xhtml

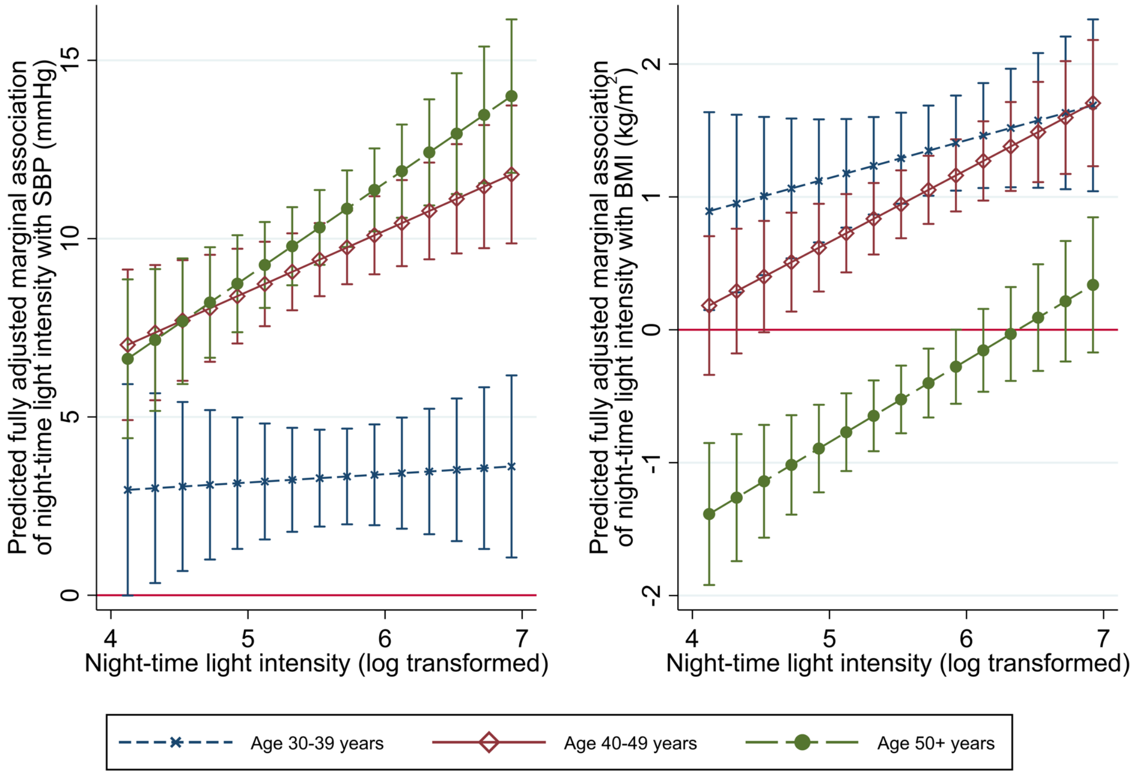

Supplementary Fig. 2 Fully adjusted marginal associations of NTLI with BMI and SBP by age groups (n=5937)

BMI – body mass index; CI – confidence interval; NTLI – night-time light intensity; SBP – systolic blood pressure

Marginal associations, including 95% CIs, were obtained from contrasting age-specific results from multilevel linear regression models with clustering by household and village, and adjusted for gender, caste, religion, marital status and survey season, using individual-level outcome data

Participants were excluded from analysis of SBP if medicated for hypertension

For peer review only - http://bmjopen.bmj.com/site/about/guidelines.xhtml

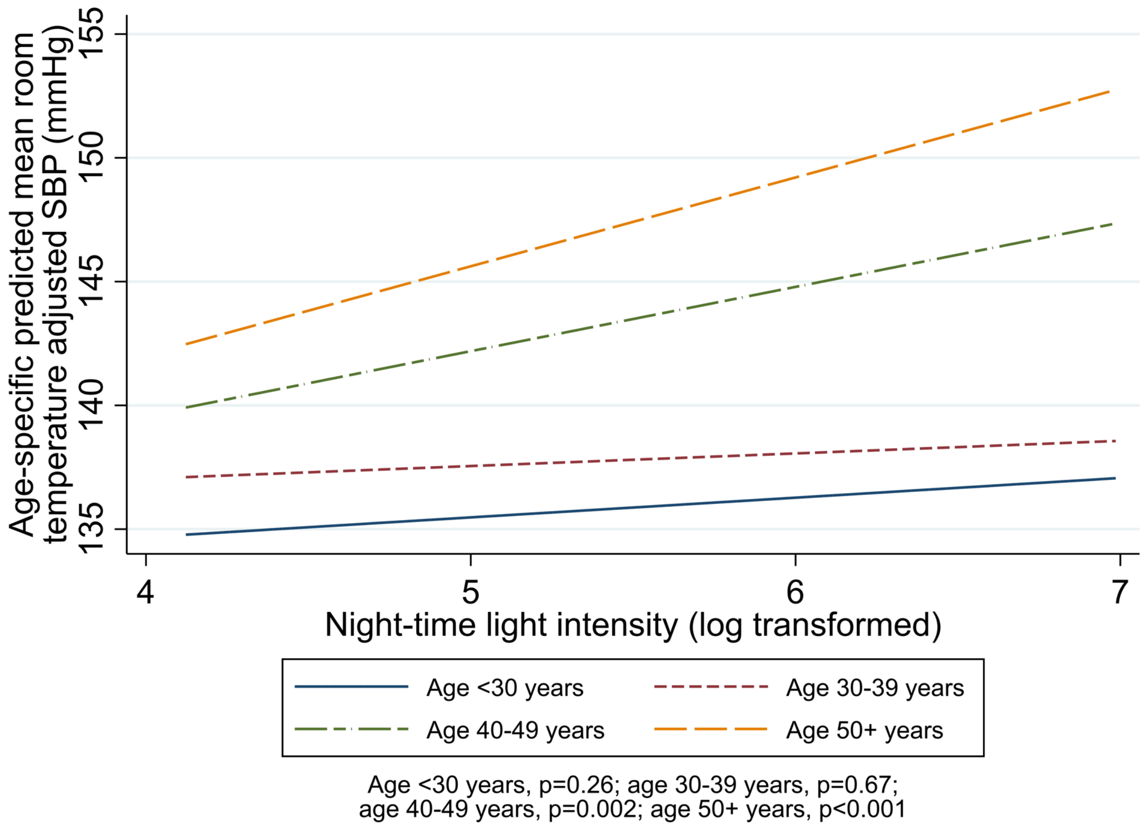

Supplementary Fig. 3 Age-specific room temperature adjusted association of NTLI and SBP among APCAPS adults, 2010-12 (n=3160)

APCAPS – Andhra Pradesh Children and Parents Study; NTLI – night-time light intensity; SBP – systolic blood pressure

The fitted lines (model predicted means) were derived from a crude multilevel linear regression models with clustering by household and village, using individual-level outcome data

Participants were excluded if medicated for hypertensions

For peer review only - http://bmjopen.bmj.com/site/about/guidelines.xhtml

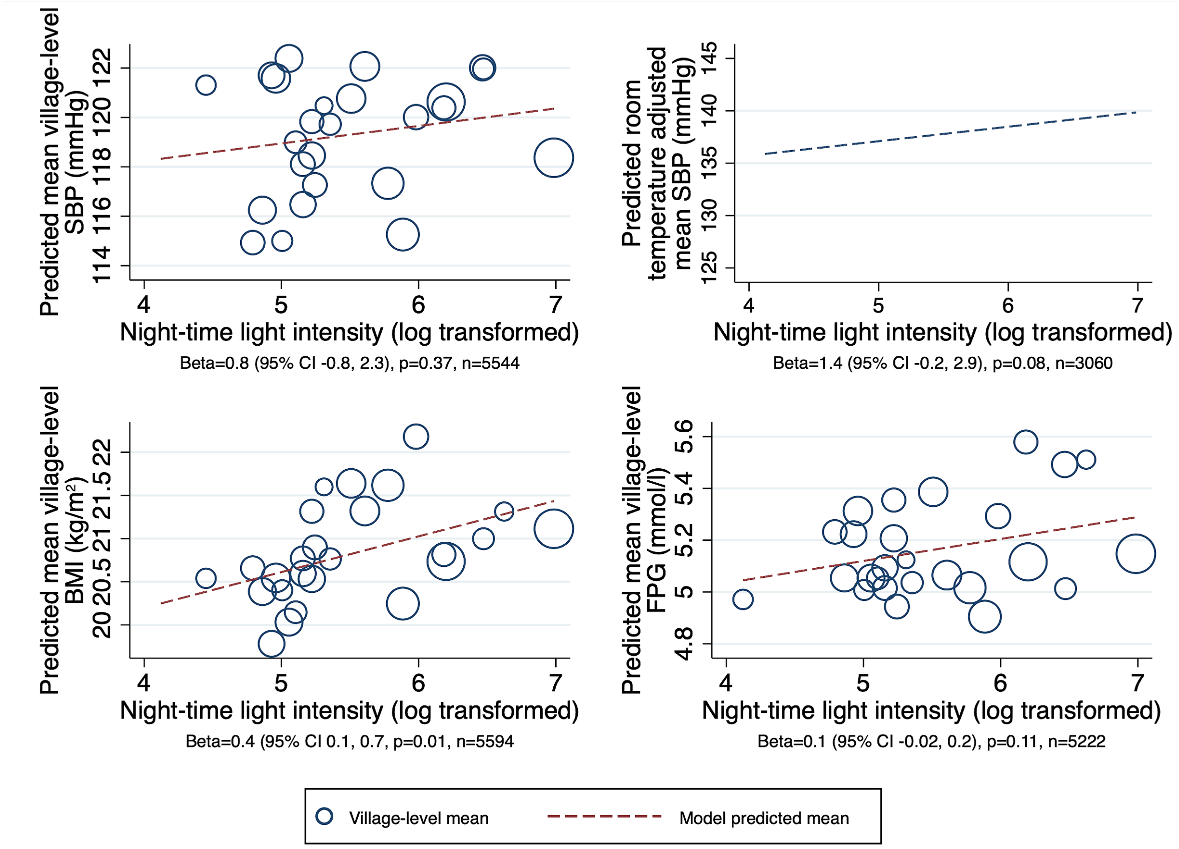

Supplementary Fig. 4 Sensitivity analysis: Crude association of NTLI with SBP, BMI and FPG among APCAPS adults, 2010-12

APCAPS – Andhra Pradesh Children and Parents Study; BMI – body mass index; FPG – fasting plasma glucose; NTLI – night-time light intensity; SBP – systolic blood pressure

Model predicted means were derived from crude and (for SBP) room temperature adjusted multilevel linear regression models with clustering by household and village, using individual-level outcome data

Marker size proportional to village size

Participants from borderline outlier villages were excluded

For peer review only - http://bmjopen.bmj.com/site/about/guidelines.xhtml

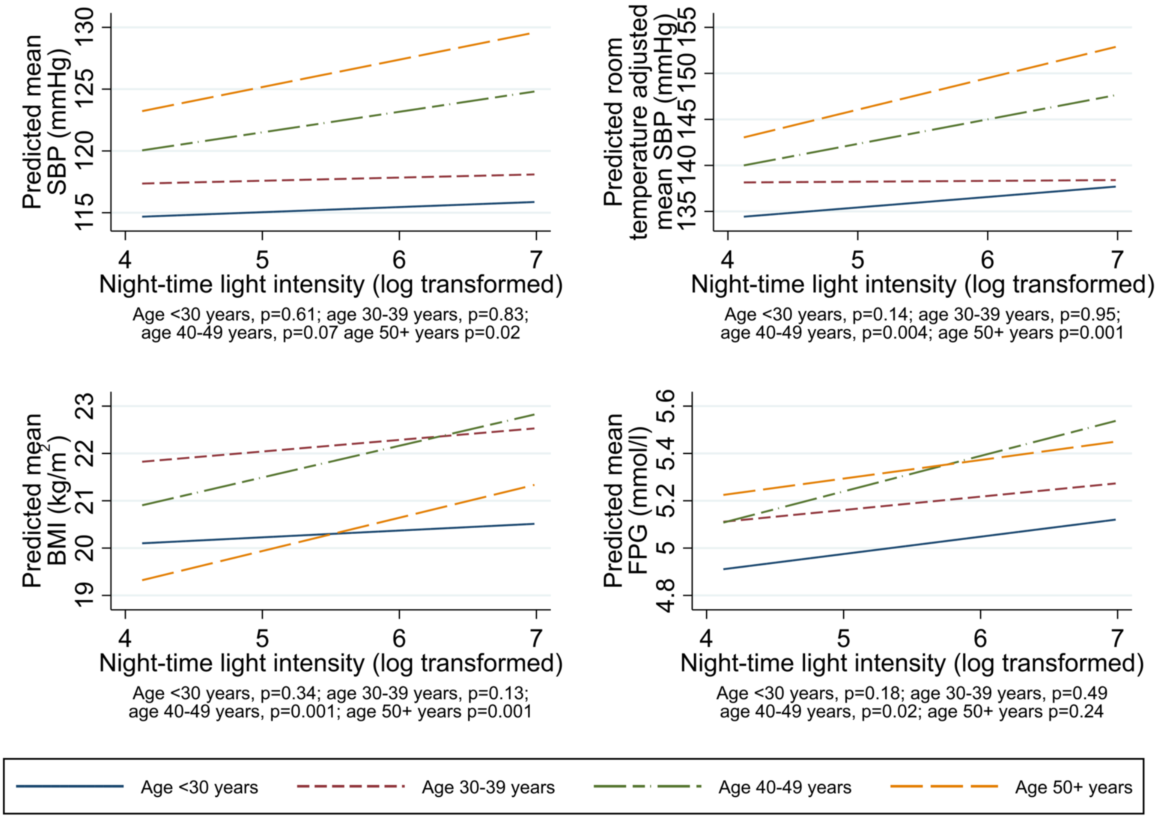

Supplementary Fig. 5 Sensitivity analysis: age-specific crude associations of NTLI with SBP, BMI and FPG

BMI – body mass index; FPG – fasting glucose; NTLI – night-time light intensity; SBP – systolic blood pressure

The fitted lines (model predicted means) were derived from a crude multilevel linear regression models with clustering by household and village, using individual-level outcome data

Participants from borderline outlier villages were excluded

Participants were excluded from analysis of SBP if medicated for hypertension and from analysis of FPG if medicated for diabetes

For peer review only - http://bmjopen.bmj.com/site/about/guidelines.xhtml

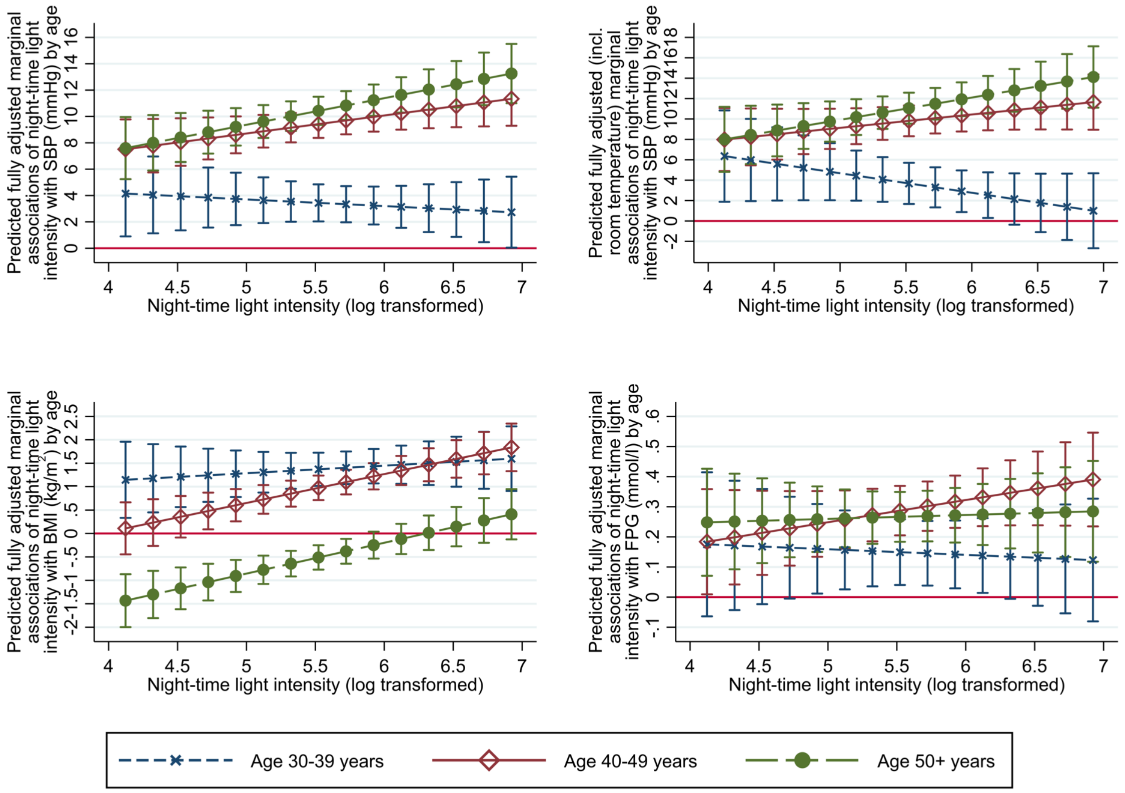

Supplementary Fig. 6 Sensitivity analysis: fully adjusted marginal associations of NTLI with SBP, BMI and FPG by age groups

BMI – body mass index; CI – Confidence Interval; FPG – fasting glucose; SBP – systolic blood pressure

Marginal associations (95% CIs) were obtained from contrasting age-specific results from multilevel linear regression models with clustering by household and village, and adjusted for gender, caste, religion, marital status and survey season, using individual level outcome data

Participants from borderline outlier villages were excluded

Participants were excluded from analysis of SBP if medicated for hypertension and from analysis of glucose if medicated for diabetes

For peer review only
For peer review only - http://bmjopen.bmj.com/site/about/guidelines.xhtml

Supplementary Tab. 1 Age-specific crude and adjusted associations of NTLI with mean BMI and SBP, APCAPS, 2010-12 (n=5937)

CVD risk factors	Age group	n (%)	Age-specific crude model predicted mean (95% CI) at the lowest NTLI (61.7 (4.1 on the log scale))	Age-specific crude model predicted mean (95% CI) at the highest NTLI (1081.1 (7.0 on the log scale))	p-value	Age-specific fully adjusted model predicted mean change with increasing NTLI[b] (adjusted for gender caste, religion, marital status and survey season)	
						β (95% CI)	p-value
SBP (mmHg)[a]	<30 years	2806 (48.8)	114.2 (111.9, 116.4)	116.6 (114.2, 119.0)	0.26	0.5 (-0.7, 1.6)	0.45
	30-39 years	544 (9.5)	115.6 (112.2, 119)	119.7 (116.5, 123.0)	0.17	0.7 (-1.2, 2.5)	0.47
	40-49 years	1306 (22.7)	119.0 (116.4, 121.6)	126.1 (123.4, 128.8)	0.004	2.2 (0.8, 3.6)	0.003
	≥50 years	1096 (19.1)	121.7 (118.9, 124.4)	131.3 (128.4, 134.2)	<0.001	3.1 (1.6, 4.6)	<0.001
BMI (kg/m^2)	<30 years	2804 (47.3)	20.2 (19.6, 20.7)	20.6 (20.1, 21.2)	0.34	0.04 (-0.3, 0.4)	0.84
	30-39 years	545 (9.2)	21.7 (20.8, 22.5)	22.8 (22.0, 23.6)	0.13	0.3 (-0.2, 0.8)	0.22
	40-49 years	1358 (22.9)	21.0 (20.4, 21.7)	22.9 (22.2, 23.5)	0.001	0.6 (0.2, 1.0)	0.01
	≥50 years	1217 (20.5)	19.4 (18.8, 20.0)	21.4 (20.7, 22.1)	0.001	0.7 (0.2, 1.1)	0.002

APCAPS – Andhra Pradesh Children and Parents Study; BMI – body mass index; CVD – cardiovascular disease; NTLI – night-time light intensity; SBP – systolic blood pressure; β – beta coefficient

Model predicted means (95% CIs), β-coefficients (95% CIs) and p-values were obtained from multilevel linear regression models with clustering by household and village, using individual-level outcome data

[a]Participants were excluded if medicated for hypertension

[b]Mean change per integer increase in log transformed NTLI

For peer review only

For peer review only - http://bmjopen.bmj.com/site/about/guidelines.xhtml

Supplementary Tab. 2 Room temperature adjusted association of NTLI and SBP among APCAPS adults, 2010-12 (n=3160)

	n	Model predicted room temperature adjusted mean (95% CI) at the lowest NTLI (61.7 (4.1 on the log scale))	Model predicted room temperature adjusted mean (95% CI) at the highest NTLI (1081.1 (7.0 on the log scale))	p-value	Model predicted age, gender and room temperature adjusted mean change (95% CI) with increasing NTLI[b]			Model predicted fully adjusted mean change with increasing NTLI[b] (adjusted for gender caste, religion, marital status, survey season and room temperature)		
					n	β (95% CI)	p-value	n	β (95% CI)	p-value
**SBP (mmHg)[a]**	3160	136.4 (129.0, 143.8)	140.7 (133.5, 148.0)	<0.001	3159	1.9 (0.7, 3.0)	0.002	3122	1.8 (0.6, 3.0)	0.002

APCAPS – Andhra Pradesh Children and Parents Study; CI – confidence interval; n – sample size; NTLI – night-time light intensity; SBP – systolic blood pressure; β – beta-coefficient

Model predicted means (95% CIs), β-coefficients (95% CIs) and p-values were obtained from multilevel linear regression with clustering by household and village, using individual-level outcome data

[a]Participants were excluded if medicated for hypertension

[b]Mean change per integer increase in log transformed NTLI

For peer review only

For peer review only - http://bmjopen.bmj.com/site/about/guidelines.xhtml

Supplementary Tab. 3 Age-specific room temperature adjusted association of NTLI and SBP among APCAPS adults, 2010-12 (n=3160)

SBP (mmHg)[a]	n (%)	Model predicted room temperature adjusted mean (95% CI) at the lowest NTLI (61.7 (4.1 on the log scale))	Model predicted room temperature adjusted mean (95% CI) at the highest NTLI (1081.1 (7.0 on the log scale))	p-value	Model predicted fully adjusted mean change with increasing NTLI[b] (adjusted for gender caste, religion, marital status, survey season and room temperature at clinical assessment)	
					β (95% CI)	p-value
<30 years	1347 (42.6)	134.8 (128.1, 141.4)	137.1 (130.4, 143.7)	0.26	0.8 (-0.5, 2.1)	0.24
30-39 years	305 (9.7)	137.1 (129.6, 144.6)	138.6 (131.3, 145.8)	0.67	-0.2 (-2.5, 2.0)	0.83
40-49 years	838 (26.5)	139.9 (133.0, 146.8)	147.3 (140.6, 154.1)	0.002	2.4 (0.8, 4.0)	0.003
≥50 years	670 (21.2)	142.5 (135.6, 149.4)	152.7 (145.8, 159.7)	<0.001	3.5 (1.8, 5.3)	<0.001

APCAPS – Andhra Pradesh Children and Parents Study; CI – confidence interval; n – sample size; NTLI – night-time light intensity; SBP – systolic blood pressure; β – beta coefficient

Model predicted means (95% CIs), β-coefficients (95% CIs) and p-values were obtained from multilevel linear regression with clustering by household and village, using individual-level outcome data

p<0.001 for effect modification by age group

[a]Participants were excluded if medicated for hypertension

[b]Mean change per integer increase in log transformed NTLI

For peer review only

For peer review only - http://bmjopen.bmj.com/site/about/guidelines.xhtml

Supplementary Tab. 4 Sensitivity analysis: crude and adjusted associations of NTLI with SBP, BMI and FPG excluding borderline outlier villages

	Model predicted crude mean change with increasing NTLI[c]			Model predicted age and gender adjusted mean change with increasing NTLI[c]			Model predicted fully adjusted mean change with increasing NTLI[c] (adjusted for age, gender, caste, religion, marital status and survey season)		
**CVD risk factors**	**n**	**β (95% CI)**	**p-value**	**n**	**β (95% CI)**	**p-value**	**n**	**β (95% CI)**	**p-value**
**SBP (mmHg)[a]**	5544	0.8 (-0.8, 2.3)	0.37	5539	1 .0 (-0.5, 2.5)	0.19	5306	0.8 (-0.4, 2.1)	0.18
**SBP (mmHg), room temperature adjusted[a]**	3060	1.4 (-0.2, 2.9)	0.08	3059	1.8 (0.5, 3.1)	0.01	3022	1.7 (0.4, 3.0)	0.01
**BMI (kg/m2)**	5594	0.4 (0.1, 0.7)	0.01	5590	0.4 (0.1, 0.7)	0.01	5352	0.3 (0.03, 0.7)	0.03
**Fasting glucose (mmol/l)[b]**	5222	0.1 (-0.02, 0.2)	0.11	5220	0.1 (-0.01, 0.2)	0.08	4988	0.1 (-0.06, 0.2)	0.37

BMI – body mass index; CI – confidence interval; CVD – cardiovascular disease; LDL – low-density lipoprotein; n – sample size; NTLI – night-time light intensity; SBP – systolic blood pressure; β – beta coefficient

β-coefficients (95% CIs) and p-values were obtained from multilevel linear regression with clustering by household and village, using individual-level outcome data

Participants from borderline outlier villages were excluded

Participants were excluded if medicated for hypertension[a] or diabetes[b]

[c]Mean change per integer increase in log transformed NTLI

For peer review only

Supplementary Tab. 5 Sensitivity analysis: age-specific crude and adjusted associations of NTLI with SBP, BMI and FPG excluding borderline outlier villages

CVD risk factors		n	Age-specific model predicted mean (95% CI) at lowest NTLI	Age-specific model predicted mean (95% CI) at highest NTLI	p-value	Age-specific fully adjusted model predicted mean change with increasing NTLI[c] (adjusted for gender caste, religion, marital status and survey season) n	β (95% CI)	p-value
**SBP (mmHg)[a]**	<30 years	2706	114.8 (112.8, 116.8)	115.9 (113.3, 118.4)	0.61	2615	0.2 (-1.1, 1.5)	0.79
	30-39 years	520	117.4 (114.4, 120.5)	118.1 (114.6, 121.5)	0.83	494	-0.3 (-2.4, 1.7)	0.76
	40-49 years	1252	120.6 (118.3, 122.9)	124.8 (121.9, 127.7)	0.07	1196	1.5 (0.02, 3.1)	0.05
	≥50 years	1066	123.9 (121.6, 126.3)	129.6 (126.5, 132.6)	0.02	1001	2.2 (0.5, 3.8)	0.01
**SBP (mmHg)[a] adjusted for room temperature at clinical assessment**	<30 years	1308	134.8 (127.9, 141.7)	137.7 (130.3, 145.1)	0.14	1294	1.0 (-0.5, 2.5)	0.20
	30-39 years	292	138.2 (130.6, 145.7)	138.4 (130.4, 146.4)	0.95	285	-0.8 (-3.4, 1.8)	0.54
	40-49 years	809	140.9 (133.8, 148.0)	147.6 (140.1, 155.2)	0.004	799	2.3 (0.6, 4.1)	0.01
	≥50 years	651	144.2 (137.1, 151.2)	152.9 (145.2, 160.6)	0.001	644	3.2 (1.3, 5.1)	0.001
**BMI (kg/m2)**	<30 years	2653	20.1 (19.7, 20.6)	20.5 (20.0, 21.1)	0.42	2563	0.02 (-0.3, 0.4)	0.92
	30-39 years	508	21.9 (21.2, 22.6)	22.5 (21.7, 23.3)	0.37	482	0.2 (-0.3, 0.7)	0.51
	40-49 years	1272	21.1 (20.6, 21.6)	22.8 (22.2, 23.5)	0.001	1213	0.6 (0.2, 1.0)	0.002
	≥50 years	1161	19.6 (19.0, 20.1)	21.3 (20.7, 22.0)	<0.001	1094	0.7 (0.3, 1.1)	0.001
**Fasting plasma glucose[b]**	<30 years	2537	4.9 (4.7, 5.1)	5.1 (4.9, 5.3)	0.20	2450	0.03 (-0.1, 0.2)	0.59
	30-39 years	491	5.1 (4.9, 5.4)	5.3 (5.0, 5.5)	0.49	465	0.01 (-0.2, 0.2)	0.88
	40-49 years	1170	5.1 (4.9, 5.3)	5.5 (5.3, 5.7)	0.02	1114	0.1 (-0.03, 0.2)	0.13
	≥50 years	1024	5.2 (5.0, 5.4)	5.4 (5.2, 5.7)	0.25	959	0.05 (-0.1, 0.2)	0.52

For peer review only

BMI – body mass index; CI - confidence interval; n – sample size; NTLI – night-time light intensity; SBP – systolic blood pressure; β – beta coefficient

Model predicted means (95% CIs), β-coefficients (95% CIs) and p-values were obtained from multilevel linear regression models with clustering by household and village, using individual-level outcome data

Participants from borderline outlier villages were excluded

Participants were excluded if medicated for hypertension[a] or diabetes[b]

[c]Mean change per integer increase in log transformed NTLI

For peer review only

For peer review only - http://bmjopen.bmj.com/site/about/guidelines.xhtml

STROBE 2007 (v4) checklist of items to be included in reports of observational studies in epidemiology*
Checklist for cohort, case-control, and cross-sectional studies (combined)

Section/Topic	Item #	Recommendation	Reported on page #
**Title and abstract**	1	(*a*) Indicate the study's design with a commonly used term in the title or the abstract	2
		(*b*) Provide in the abstract an informative and balanced summary of what was done and what was found	2
**Introduction**			
Background/rationale	2	Explain the scientific background and rationale for the investigation being reported	4-5
Objectives	3	State specific objectives, including any pre-specified hypotheses	5
**Methods**			
Study design	4	Present key elements of study design early in the paper	5+
Setting	5	Describe the setting, locations, and relevant dates, including periods of recruitment, exposure, follow-up, and data collection	
Participants	6	(*a*) *Cohort study*—Give the eligibility criteria, and the sources and methods of selection of participants. Describe methods of follow-up<br>*Case-control study*—Give the eligibility criteria, and the sources and methods of case ascertainment and control selection. Give the rationale for the choice of cases and controls<br>*Cross-sectional study*—Give the eligibility criteria, and the sources and methods of selection of participants	5+
		(*b*) *Cohort study*—For matched studies, give matching criteria and number of exposed and unexposed<br>*Case-control study*—For matched studies, give matching criteria and the number of controls per case	n/a
Variables	7	Clearly define all outcomes, exposures, predictors, potential confounders, and effect modifiers. Give diagnostic criteria, if applicable	6-7
Data sources/ measurement	8*	For each variable of interest, give sources of data and details of methods of assessment (measurement). Describe comparability of assessment methods if there is more than one group	6-7
Bias	9	Describe any efforts to address potential sources of bias	8, 14-15
Study size	10	Explain how the study size was arrived at	9, flow diagram
Quantitative variables	11	Explain how quantitative variables were handled in the analyses. If applicable, describe which groupings were chosen and why	8
Statistical methods	12	(*a*) Describe all statistical methods, including those used to control for confounding	8
		(*b*) Describe any methods used to examine subgroups and interactions	8
		(*c*) Explain how missing data were addressed	9, Flow diagram
		(*d*) *Cohort study*—If applicable, explain how loss to follow-up was addressed<br>*Case-control study*—If applicable, explain how matching of cases and controls was addressed	8

For peer review only

		Cross-sectional study—If applicable, describe analytical methods taking account of sampling strategy	
		(*e*) Describe any sensitivity analyses	8
**Results**			
Participants	13*	(a) Report numbers of individuals at each stage of study—eg numbers potentially eligible, examined for eligibility, confirmed eligible, included in the study, completing follow-up, and analysed	9, flow chart
		(b) Give reasons for non-participation at each stage	Flow diagram
		(c) Consider use of a flow diagram	Flow diagram
Descriptive data	14*	(a) Give characteristics of study participants (eg demographic, clinical, social) and information on exposures and potential confounders	9-10
		(b) Indicate number of participants with missing data for each variable of interest	Flow diagram
		(c) *Cohort study*—Summarise follow-up time (eg, average and total amount)	
Outcome data	15*	*Cohort study*—Report numbers of outcome events or summary measures over time	
		Case-control study—Report numbers in each exposure category, or summary measures of exposure	
		Cross-sectional study—Report numbers of outcome events or summary measures	11
Main results	16	(*a*) Give unadjusted estimates and, if applicable, confounder-adjusted estimates and their precision (eg, 95% confidence interval). Make clear which confounders were adjusted for and why they were included	11
		(*b*) Report category boundaries when continuous variables were categorized	8
		(*c*) If relevant, consider translating estimates of relative risk into absolute risk for a meaningful time period	10-12
Other analyses	17	Report other analyses done—eg analyses of subgroups and interactions, and sensitivity analyses	12
**Discussion**			
Key results	18	Summarise key results with reference to study objectives	12-13
Limitations	19	Discuss limitations of the study, taking into account sources of potential bias or imprecision. Discuss both direction and magnitude of any potential bias	14-15
Interpretation	20	Give a cautious overall interpretation of results considering objectives, limitations, multiplicity of analyses, results from similar studies, and other relevant evidence	3, 14
Generalisability	21	Discuss the generalisability (external validity) of the study results	14
**Other information**			
Funding	22	Give the source of funding and the role of the funders for the present study and, if applicable, for the original study on which the present article is based	17

*Give information separately for cases and controls in case-control studies and, if applicable, for exposed and unexposed groups in cohort and cross-sectional studies.

Note: An Explanation and Elaboration article discusses each checklist item and gives methodological background and published examples of transparent reporting. The STROBE checklist is best used in conjunction with this article (freely available on the Web sites of PLoS Medicine at http://www.plosmedicine.org/, Annals of Internal Medicine at http://www.annals.org/, and Epidemiology at http://www.epidem.com/). Information on the STROBE Initiative is available at www.strobe-statement.org.

For peer review only
